# Supplementary material for: Photoactivated nanomotors via aggregation induced emission for enhanced phototherapy
Source: Nat Commun. 2021 Apr 6;12:2077. doi: 10.1038/s41467-021-22279-w (PMC8024279; doi:10.1038/s41467-021-22279-w)
Supplement: Supplementary file 1 — Supplementary information [file 41467_2021_22279_MOESM1_ESM.pdf]

Supporting Information

Photoactivated Nanomotors *via* Aggregation Induced Emission for Enhanced Phototherapy

Shoupeng Cao[1,4], Jingxin Shao[1,4], Hanglong Wu[1], Shidong Song[1], Maria Teresa De Martino[1], Imke A. B. Pijpers[1], Heiner Friedrich[2], Loai K. E. A. Abdelmohsen[1]*, David S. Williams[3]*, and Jan C. M. van Hest[1]*

[1]Bio-Organic Chemistry, Institute for Complex Molecular Systems, Eindhoven University of Technology, P.O. Box 513 (STO 3.41), 5600 MB Eindhoven, the Netherlands

[2]Center for Multiscale Electron Microscopy (CMEM) and Department of Chemical Engineering and Chemistry, Physical Chemistry, Institute for Complex Molecular Systems (ICMS), Eindhoven University of Technology, 5600 MB Eindhoven, The Netherlands

[3]Department of Chemistry, College of Science, Swansea University, Swansea, UK

[4]These authors contributed equally to this work: Shoupeng Cao, Jingxin Shao.

*Corresponding should be address to: l.k.e.a.abdelmohsen@tue.nl; d.s.williams@swansea.ac.uk; j.c.m.v.hest@tue.nl

Supplementary Notes:

Nuclear Magnetic Resonance Spectroscopy (NMR): Routine proton nuclear magnetic resonance (^{1}H NMR) and carbon nuclear magnetic resonance (^{13}C NMR) measurements were conducted on a Bruker Avance 400 MHz Ultrashield^{TM} spectrometer equipped with a Bruker SampleCase auto-sampler, using $CDCl_3$ or DMSO-d_6 as the solvent and TMS as the internal standard.

Size-exclusion chromatography (SEC): The molecular weights and dispersity of the block copolymer were characterized by using a Prominence-I GPC system (Shimadzu) with a PL gel 5 μm mixed D (Polymer Laboratories) and equipped with an RID-20A differential refractive index detector. Polystyrene standards were used for calibration. THF was used as an eluent with a flow rate of 1 mL per minute.

Dynamic Light Scattering (DLS): The hydrodynamic size of the polymersomes was measured by using a Malvern Instruments Zetasizer (model Nano ZSP) equipped with a 633 nm He-Ne laser and an avalanche photodiode detector. Zetasizer software was used to process and analyze the data.

Scanning Electron Microscopy (SEM): Morphology of both polymersomes and Janus structures was characterized by SEM (FEI Quanta 200 3D FEG).

Transmission Electron Microscopy (TEM): TEM images were recorded by a FEI Tecnai 20 (type Sphera) at 200 kV. Measurements were performed by dropping 10 μL samples in MilliQ onto a carbon-coated copper grid. After removing the excess solution by blotting paper, the samples were dried at ambient conditions. For staining, a drop of phosphotungstic acid (2 %) solution was placed on the grid for 30 s.

Cryogenic transmission electron microscopy (cryo-TEM) and cryo-electron tomography (cryo-ET):

Cryo-TEM and cryo-ET were carried out on the TU/e CryoTitan equipped with a field-mission gun operating at 300 kV, a post-column Gatan energy filter and an autoloader station. Samples for cryo-TEM were prepared by treating the grids (Cryo-TEM: Lacey carbon coated, R2/2, Cu, EM sciences. Cryo-ET: R2/2, Cu, Quantifoil Jena grids, Quantifoil Micro Tools GmbH) in a Cressington 208 carbon coater for 40 seconds. Then, 3 μl of the nanoparticle solution was pipetted on the grid and blotted in a Vitrobot MARK IV at 100% humidity. Note that 20 nm gold nanoparticles were added into an aliquot of the dispersion and are used as fiducial markers for cryo-ET. The grid was blotted for 3.5 seconds (blotting force -1) and directly plunged and frozen in liquid ethane. Images were acquired using a post-GIF 2k Gatan CCD (charge-coupled device) camera.

Cryo-TEM tomography tilt-series acquisition was performed using Inspect 3D software (FEI company). Alignment of the tilt-series and tomographic reconstruction were carried out in IMOD using the simultaneous iterative reconstruction technique (SIRT, 30 iterations). The cryo-ET acquisition conditions are shown below:

Angular sampling: –66° to 66° at 3° increments;

Magnification: 15000 ×; Defocus: –2.5 μm;

Total image number: 45; Total electron dose: ~ 1.8 $e^{-}.Å^{-2}$/frame;

Two Photon-Confocal Laser Scanning Microscopy (TP-CLSM): Fluorescent images were observed and recorded by using CLSM (Leica TCS SP5X) equipped with two-photon laser source (Chameleon Vision, Coherent, USA), each two-photon scan represents 4 s laser irradiation.

Microplate Reader: Cell viability was evaluated via a microplate reader (Safire2, TECAN). The reaction progress was monitored via the fluorescent signal from fluorescein on the Spark ® 10M microplate reader (TECAN).

UV-vis spectroscopy: UV-vis spectra were characterized by using UV-vis spectroscopy (V-650, JASCO).

NanoSight Tracking Analysis (NTA): Nanosight Tracking Analysis was carried out on a Nanosight NS300 equipped with different laser channels (405 nm and 488 nm) and sCMOS camera. In order to measure particle autonomous motion at 660 nm, the instrument was equipped with an Electron Multiplication Charge Coupled Device and external laser source (660 nm, BeamQ Lasers).

Supplementary Methods:

Synthesis route towards amino group functionalized AIE photosensitizer:

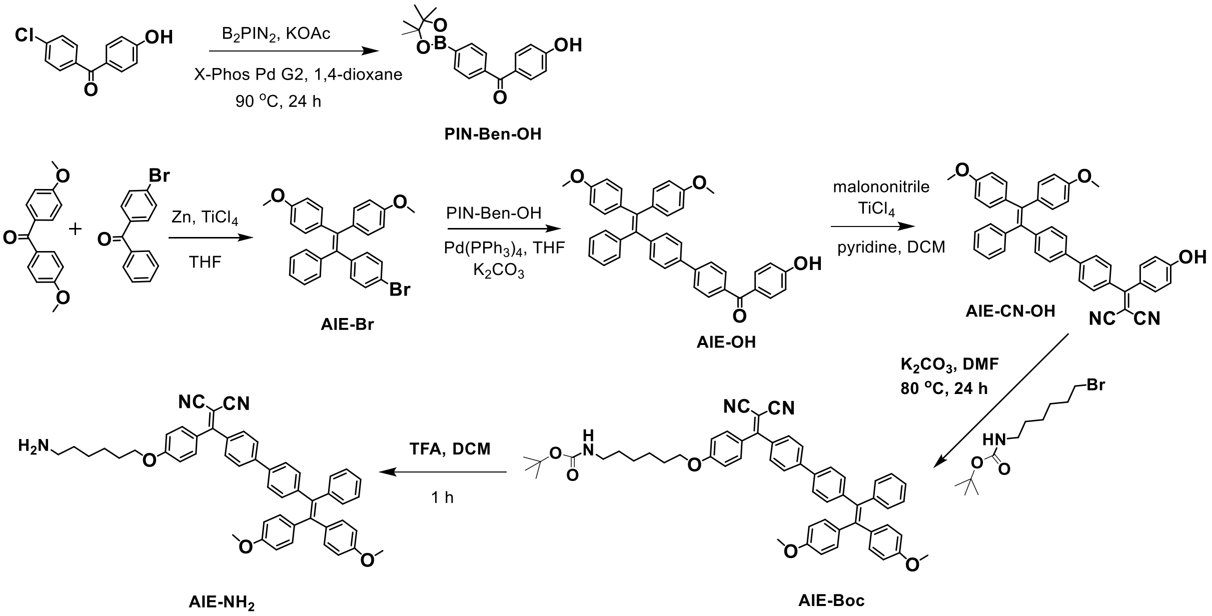

Compound **PIN-Ben-OH**: This compound was synthesized according to a previously reported procedure with slight modifications.[1] Under nitrogen environment at room temperature, X-Phos Pd G2 precatalyst (190 mg, 0.24 mmol, 0.05 equiv), bis(pinacolato)diboron (B_2Pin_2) (7.32 g, 28.8 mmol, 6.00 equiv), potassium acetate (KOAc, 2.83 g, 28.8 mmol, 6.00 equiv), and 4-chloro-4-hydroxybenzophenone (1.11 g, 4.8 mmol, 1.00 equiv) were added into a 500 mL round-bottom flask vial equipped with a stir bar. Thereafter, anhydrous 1,4-dioxane (250 mL) was added, and the vial was sealed with a screwcap and placed in a mantle heated to 90 °C. After being stirred (500 rpm) for 24 h, the formed golden-brown mixture was allowed to cool to room temperature, diluted with EtOAc (100

mL), suction-filtered through a short plug of silica gel, after which the plug was rinsed with EtOAc. This resulted in a pale yellow filtrate solution which was concentrated and separated via silica gel chromatography. The crude product was used for the synthesis of **AIE-OH** without further purification.

Compound **AIE-Br**: This compound was synthesized according to a previously reported literature procedure.[2] Briefly, bis(4-methoxyphenyl) methanone (9.73 g, 40.0 mmol), 4-bromo-benzophenone (12.54 g, 48.0 mmol), and Zn powder (13 g, 200 mmol) were dissolved in dry THF pre-cooled on an ice-bath (500 mL) under argon atmosphere. While stirring, titanium tetrachloride ($TiCl_4$, 17.6 mL, 160 mmol) was slowly added into the reaction mixture. Subsequently, the mixture was heated to 80 °C. After 24 h, the reaction mixture was left to cool down to room temperature. Afterwards, the reaction was quenched by the addition of 250 mL 10% aq K_2CO_3, and the mixture was filtered to remove insoluble materials and washed with CH_2Cl_2. The organic layer was dried with anhydrous $MgSO_4$ and filtered. The CH_2Cl_2 solution was removed by evaporation. The crude product was purified by column chromatography to give 9.62 g (20.4 mmol) of **AIE-Br** as a light yellow solid in 51% yield.^{1}H NMR (400 MHz, $CDCl_3$) *δ* (ppm): 7.21 (d, J = 8.3 Hz, 2H), 7.16 – 7.04 (m, 3H), 7.05 – 6.96 (m, 2H), 7.01 – 6.85 (m, 6H), 6.70 – 6.59 (m, 4H), 3.75 (d, J = 10.9 Hz, 6H).

Compound **AIE-OH**: This compound was synthesized according to a previously reported procedure with slight modifications.[3] Compound **PIN-Ben-OH** (972 mg, 3.0 mmol), **AIE-Br** (1550 mg, 3.3 mmol), potassium carbonate (4140 mg, 30 mmol), THF (90 mL)/water (30 mL), and $Pd(PPh_3)_4$ (3 %), were degassed and charged with nitrogen. The reaction mixture was then stirred at 60 ^{o}C for 12 h. After cooling down the reaction mixture to ambient temperature, it was extracted with dichloromethane and washed with water. The dichloromethane layer was separated and dried over $MgSO_4$. After dichloromethane evaporation by rotavap, the crude product was purified by column chromatography on silica gel using n-hexane/dichloromethane (1/1, v/v) as the eluent to afford **AIE-OH** as a yellow solid (1234 mg, 71 % yield). ^{1}H NMR (400 MHz, $CDCl_3$) *δ* (ppm): 7.86 – 7.76 (m, 4H), 7.69 – 7.62 (m, 2H), 7.43 – 7.37 (m, 2H), 7.19 – 7.06 (m, 7H), 7.01 – 6.89 (m, 6H), 6.72 – 6.61 (m, 4H), 3.74 (s, 6H). ^{13}C NMR (100 MHz, $CDCl_3$) δ 195.40, 159.89, 158.22, 158.13, 144.40, 144.14, 140.68, 138.57, 137.21, 136.55, 136.26, 132.81, 132.63, 132.60, 132.00, 131.44, 130.43, 130.36, 127.79, 126.54, 126.41, 126.23, 115.20, 113.16, 113.03, 77.34, 77.02, 76.70, 55.13, 55.11. MALDI TOF-MS: m/z calculated for $[M]^+$ $C_{41}H_{32}O_4$, 588.23, found 588.24.

Compound **AIE-CN-OH**: This compound was synthesized according to a previously reported procedure with slight modifications.[3] To the solution of compound **AIE-OH** (588 mg, 1.0 mmol) and malononitrile (198 mg, 3.0 mmol) in dichloromethane (100 mL) was added titanium tetrachloride (0.4 mL, 3.5 mmol) slowly under ice-bath. After the reaction mixture was stirred for 30 min, pyridine (0.3 mL, 3.5 mmol) was injected and stirred for another 30 min. Then the mixture was heated at 40 ^{o}C for 4 h. After the mixture was cooled down to room temperature, the reaction was quenched using water (30

mL) and the mixture was extracted with dichloromethane. The collected organic layer was washed with brine, dried over Na_2SO_4 and concentrated under reduced pressure. The residue was purified by column chromatography using n-hexane/dichloromethane (1/1 ~ 1/2, v/v) as eluent to give the desired product **AIE-CN-OH** as a red solid (534 mg, yield 84 %). ^{1}H NMR (400 MHz, $CDCl_3$) δ (ppm): 7.72 – 7.63 (m, 2H), 7.51 – 7.45 (m, 2H), 7.45 – 7.37 (m, 4H), 7.17 – 7.02 (m, 7H), 7.01 – 6.86 (m, 6H), 6.71 – 6.62 (m, 4H), 3.75 (s, 6H). ^{13}C NMR (100 MHz, $CDCl_3$) δ (ppm): 174.19, 160.27, 158.26, 158.18, 145.06, 144.90, 144.04, 140.89, 138.44, 136.53, 136.18, 136.16, 134.74, 133.27, 132.71, 132.63, 132.60, 132.10, 131.42, 131.26, 128.18, 127.81, 126.97, 126.37, 126.28, 115.89, 113.16, 113.04, 78.24, 77.34, 77.02, 76.70, 55.14, 55.11. MALDI TOF-MS: m/z calculated for $[M]^+$ $C_{44}H_{32}N_2O_3$, 636.24, found 636.26.

Compound **AIE-Boc**: This compound was synthesized according to a previously reported procedure with slight modifications.[4] A mixture of **AIE-CN-OH** (1.75 g, 2.76 mmol), 6-bromo-1-hexanol (500 mg, 2.76 mmol), and K_2CO_3 (950 mg, 6.90 mmol) in DMF (100 mL) was stirred at 80 °C overnight under nitrogen atmosphere. The reaction mixture was cooled to room temperature and filtered through a pad of celite, washed with CH_2Cl_2. The CH_2Cl_2 solution was then dried with $MgSO_4$ and filtered. The solvent was removed by evaporation. The crude mixture was purified using column chromatography on silica gel using n-hexane/dichloromethane (4/1 ~ 2/1, v/v). The product was obtained as a red solid (2.00 g, 89% yield). ^{1}H NMR (400 MHz, $CDCl_3$) δ (ppm): 7.71 – 7.63 (m, 2H), 7.49 – 7.43 (m, 4H), 7.43 – 7.37 (m, 2H), 7.12 (m, 5H), 7.05 (m, 2H), 7.00 – 6.90 (m, 6H), 6.70 – 6.61 (m, 4H), 4.51 (s, 1H), 4.03 (t, J = 6.4 Hz, 2H), 3.75 (d, J = 1.3 Hz, 6H), 3.13 (q, J = 6.6 Hz, 2H), 1.82 (m, 2H), 1.50 (m, 4H), 1.44 (s, 9H), 1.26 (m, 2H). ^{13}C NMR (100 MHz, $CDCl_3$) δ ppm: 173.94, 163.08, 158.26, 158.17, 155.99, 144.92, 144.85, 144.04, 140.86, 138.43, 136.57, 136.16, 134.86, 133.03, 132.62, 132.59, 132.08, 131.42, 131.24, 127.80, 126.92, 126.36, 114.68, 113.14, 113.02, 78.23, 77.33, 77.01, 76.70, 68.27, 55.12, 55.10, 40.47, 30.05, 28.97, 28.43, 26.49, 25.70. MALDI TOF-MS: m/z calculated for $[M]^+$ $C_{55}H_{53}N_3O_5$, 835.40, found 835.42.

Compound **AIE-NH$_2$**: **AIE-Boc** (1.67 g, 2 mmol) was dissolved in DCM (10 mL) and immersed in an ice bath. Then, 5 mL trifluoroacetic acid was added dropwise under nitrogen atmosphere. The reaction mixture was allowed to warm to room temperature and reacted for another 1 h. The solvent was removed, and the crude mixture was dissolved in CH_2Cl_2, washed with water, and dried with $MgSO_4$. Then the CH_2Cl_2 solvent was removed via evaporation and the product was obtained as a red solid (1.38 g, 94% yield). ^{1}H NMR (400 MHz, $CDCl_3$) δ (ppm): 7.65 (d, J = 8.1 Hz, 2H), 7.49 – 7.36 (m, 6H), 7.15 – 7.02 (m, 7H), 7.00 – 6.87 (m, 6H), 6.65 (m, 4H), 4.03 (t, J = 6.2 Hz, 2H), 3.74 (d, J = 1.9 Hz, 6H), 3.10 – 3.00 (m, 2H), 1.81 (m, 2H), 1.50 – 1.38 (m, 4H), 1.27 (m, 2H). ^{13}C NMR (100 MHz, $CDCl_3$) δ (ppm): 174.54, 163.08, 158.23, 158.15, 145.15, 144.93, 144.02, 140.90, 138.47, 136.51, 136.23, 136.19, 134.68, 133.05, 132.64, 132.60, 132.11, 131.42, 131.30, 128.01, 127.82, 126.96, 126.38, 126.29, 114.72, 113.19, 113.06, 77.85, 77.34, 77.02, 76.70, 67.91, 55.14, 40.53, 28.56, 27.60, 27.25, 25.28. MALDI TOF-MS: m/z calculated for $[M]^+$ $C_{50}H_{45}N_3O_3$, 735.35, found 735.34.

Synthesis route towards AIE photosensitizer functionalized block-co-polymers:

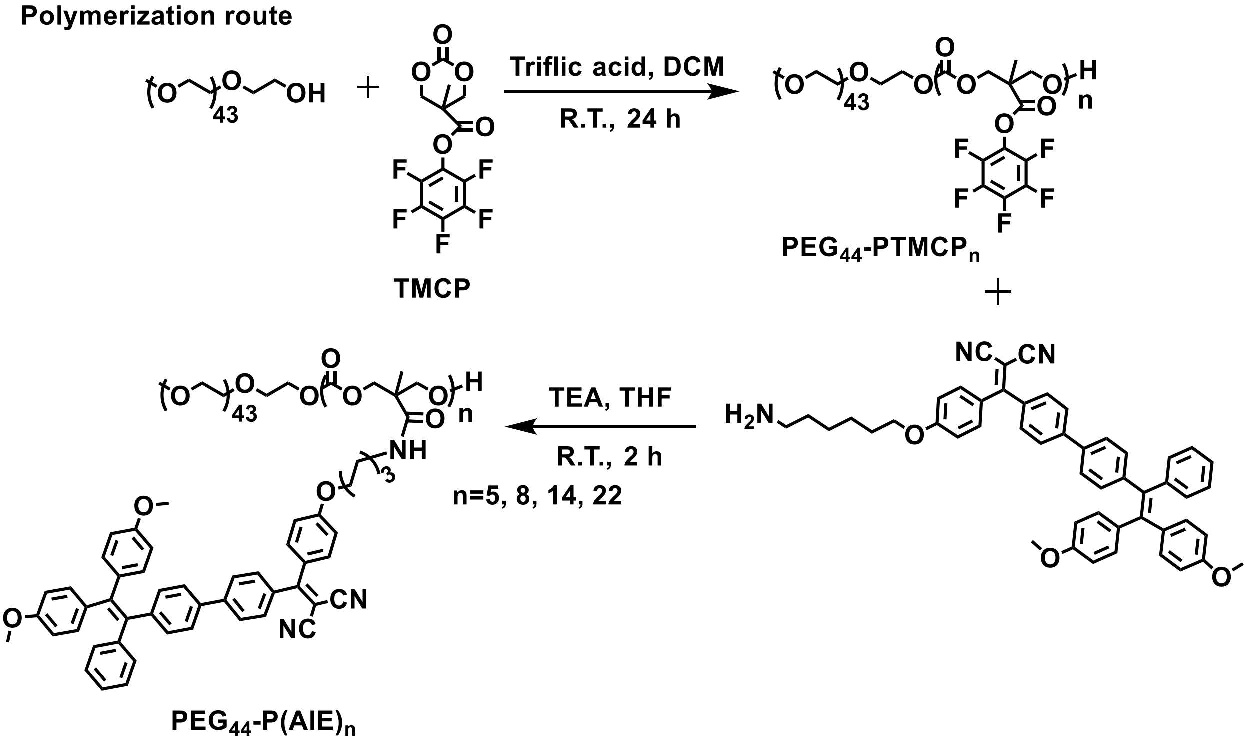

Synthesis of **TMCP**: This compound was synthesized according to a previously reported procedure.[5] A 250 mL round bottom flask was charged with 2,2-bis(hydroxymethyl)propionic acid (bis-MPA) (3.00 g, 22 mmol), bis-(pentafluorophenyl)carbonate (21.70 g, 55 mmol, 2.5 eq.), CsF (0.7 g, 4.6 mmol, 0.2 eq.), and 120 mL of anhydrous tetrahydrofuran (THF). After 21 h, the solvent THF was removed in vacuum. The residue was re-dissolved in methylene chloride and, after 10 min, a byproduct precipitated and was removed by filtration. The filtrate was extracted with sodium bicarbonate and water, and then was dried with $MgSO_4$. The solvent was evaporated in vacuum and the product was recrystallized from ethyl acetate/hexane mixture to give TMC-OPh_5 (TMCP) as a white crystalline powder. Yield: 5.13 g (70 % yield). ^1H NMR (400 MHz, $CDCl_3$), δ ppm: 4.85 (d, J = 10.8 Hz, 2H,), 4.36 (d, J = 10.8 Hz, 2H,), 1.55 (s, 3H). ^{19}F NMR (376 MHz, $CDCl_3$), δ ppm: 154.0~154.1 (m, 2F), 157.3 (t, 1F), 162.4~162.5 (m, 2F).

Ring-opening polymerization (ROP) of **TMCP** to obtain **PEG-PTMCP**: Taking PEG_{44}-$PTMCP_5$ as an example, the synthesis of PEG-PTMCP was performed according to a reported procedure.[6] Monomethyl-PEG-OH macro-initiator 2 kDa (984 mg, 0.5 mmol, 1.0 equiv) and dry TMCP (978 mg, 3 mmol, 6 equiv) were added into a round bottom flask. Under argon flow, 3 mL dichloromethane (DCM) (1 M with respect to TMCP) was added into the flask. The TMCP only partially dissolves at this concentration. Triflic acid (75 μL, 1 mmol, 2 equiv) was added to the stirring solution. As the reaction proceeded, the undissolved TMCP slowly went into solution. The reaction was monitored by ^1H NMR. Once the reaction was complete, the polymer was precipitated into hexanes. The crude

polymer was then re-dissolved in minimal amount of DCM and precipitated into diethyl ether, isolated, and freeze dried to obtain the product, and kept in the freezer until being used. Copolymer composition was calculated by using the protons of PEG (3.65-3.7 ppm), the terminal methyl unit (singlet at 3.38 ppm), TMCP CH_2 (singlet, 4.44-4.48), and TMCP CH_3 (singlet, 1.48-1.50). GPC (RI): M_n (PDI) = 3.88 kDa (1.08). PEG_{44}-$PTMCP_8$, PEG_{44}-$PTMCP_{14}$, and PEG_{44}-$PTMCP_{22}$ were synthesized using the same synthetic method, GPC (RI) for PEG_{44}-$PTMCP_8$: M_n (PDI) = 4.38 kDa (1.10); GPC (RI) for PEG_{44}-$PTMCP_{14}$: M_n (PDI) = 6.31 kDa (1.08); GPC (RI) for PEG_{44}-$PTMCP_{22}$: M_n (PDI) = 7.72 kDa (1.13), the details are shown in Table S1.

Synthesis of **PEG-P(AIE)** block-co-polymers: Taking PEG_{44}-$P(AIE)_5$ as an example, freeze dried PEG_{44}-$PTMCP_5$ (72 mg, 0.02 mmol, 1 equiv) was dissolved in 1 ml dry THF and cooled on an ice-bath. Next, a 0.5 mL THF solution containing **AIE-NH₂** (117 mg, 0.12 mmol, 1.15 equiv. with respect to the pentafluorophenyl ester) and trimethylamine (TEA, 22.3 μL, 0.12 mmol, 1.15 equiv. with respect to the pentafluorophenyl ester) were dropwise added. The ice bath was removed and the mixture was allowed to stir for an additional 90 minutes. The reaction was monitored by ^{1}HNMR and ^{19}F NMR. After the complete conversion of pentafluorophenyl ester, the reaction solution was precipitated into diethyl ether three times; the absence of free **AIE-NH₂** was confirmed by GPC. Then the yellow solid was isolated and freeze dried to obtain PEG_{44}-$P(AIE)_5$. Copolymer composition was calculated by using the protons of PEG (3.61-3.68 ppm), terminal methyl unit (singlet at 3.38 ppm), TMCAIE CH_2 (m, 3.70-3.76 ppm) and TMCAIE CH_2 (m, 4.22-4.32 ppm), and TMCAIE CH_2 (m, 6.60-6.66 ppm). GPC (RI): M_n (PDI) = 8.29 kDa (1.11). PEG_{44}-$P(AIE)_8$, PEG_{44}-$P(AIE)_{14}$, and PEG_{44}-$P(AIE)_{22}$ were synthesized via similar procedures. GPC (RI) for PEG_{44}-$P(AIE)_8$: M_n (PDI) = 9.37 kDa (1.10); GPC (RI) for PEG_{44}-$P(AIE)_{14}$: M_n (PDI) = 11.02 kDa (1.09); GPC (RI) for PEG_{44}-$P(AIE)_{22}$: M_n (PDI) = 14.98 kDa (1.11), the details are shown in Table S1.

Preparation of AIE-polymersomes. Taking PEG_{44}-$P(AIE)_5$ as an example, in a 4 mL vial, PEG_{44}-$P(AIE)_5$ (1 mg) was dissolved in 0.5 mL of THF and the vial was sealed with a rubber septum. The solution was stirred at 700 rpm for a minimum of 10 minutes prior to the addition of Milli-Q (0.5 mL, 0.25 mL h^{-1}) via a syringe pump. A needle was inserted into the septum to release pressure. The resulting cloudy suspension was transferred into a prehydrated dialysis bag (SpectraPor, MWCO: 12-14 kDa, 2 mL cm^{-1}). Dialysis was performed against Milli-Q water at room temperature for 24 hours with a water change after 1 hour. The physicochemical properties of AIE-polymersomes were characterized by dynamic light scatting, scanning electron microscopy, transmission electron microscopy, cryo transmission electron microscopy and confocal laser scanning microscopy.

Preparation of Janus AIE/Au nanomotors. In order to construct AIE/Au nanomotors, a droplet of the AIE-polymersome solution (2 mg ml^{-1}) was dropped on a hydrophilic silica slide to form a monolayer of nanoparticles. After evaporation in air, a turbo sputter coater (Quorum Technologies, K575X) was used to coat one side of the polymeric particles with a thin gold layer (65 mV, 30 s). Ultrasound treatment was used to re-disperse the Janus polymeric particles into aqueous solution.[7] The size and morphology of AIE/Au nanomotors (AIE/Au NM) were characterized using dynamic light scatting, scanning electron microscopy, transmission electron microscopy and confocal laser scanning microscopy. EDX elemental mapping analysis of AIE/Au nanomotors was performed using SEM (Phenom ProX, The Netherlands).

Preparation of AIE/Au nanomotors loaded with cargoes. In order to test the integrity of the AIE/Au nanomotors, different cargoes (i.e., 10 kDa dextran-TMR and Cy7) loaded AIE-polymersomes were first prepared via a similar procedure compared with AIE-polymersomes; the dextran-TMR was dissolved in Milli-Q water and Cy7 was co-dissolved with the AIE-polymer. After dialysis purification, a droplet of the cargo-loaded AIE-polymersome solution (2 mg ml^{-1}) was dropped on a hydrophilic silica slide to form a monolayer of nanoparticles. After evaporation in air, a turbo sputter coater (Quorum Technologies, K575X) was used to coat one side of the polymeric particles with a thin gold layer (65 mV, 30 s). Ultrasound treatment was used to re-disperse the Janus polymeric particles into aqueous solution.[7] The fluorescent emission behavior of cargo loaded AIE/Au nanomotors (AIE/Au NM) was characterized using microplate reader.

Integrity studies of Janus AIE/Au nanomotors: the nanomotors integrity was checked by the leakage of the hydrophilic and hydrophobic cargoes. In detail, in the case of hydrophilic dextran-TMR loaded AIE/Au motors, the fresh prepared cargo loaded AIE/Au nanomotor (after ultrasound treatment and re-dispersed in aqueous medium) was divided into aliquots and incubated for different times. After certain time (30 min, 1 h, 2 h, 3 h, 4 h, and 5 h), the aliquots were centrifuged and the dextran-TMR emission intensity in the supernatant was measured using a micro-plate reader (with three replications) to determine the leakage of dextran-TMR cargo. In the case of hydrophobic Cy7 loaded AIE/Au motors, the freshly prepared cargo loaded AIE/Au nanomotors (after ultrasound treatment) were dispersed in aqueous solution. The emission intensity of Cy7 in this cargo loaded AIE/Au

nanomotor was monitored by a microplate reader (with three replications) to determine the leakage of Cy7 cargo.

Preparation of non-AIE nanomotors: non-AIE polymersomes were prepared from PEG_{44}-$PDLLA_{115}$. The polymersomes were loaded with Cy5 in the hydrophobic domain of the bilayer, following our previously published procedure.[8] In order to construct non-AIE nanomotors, a droplet of the PEG_{44}-$PDLLA_{115}$ polymersome solution (2 mg ml^{-1}) was dropped on a hydrophilic silica slide to form a monolayer of nanoparticles. After evaporation in air, a turbo sputter coater (Quorum Technologies, K575X) was used to coat one side of the polymeric particles with a thin gold layer (65 mV, 30 s). Ultrasound treatment was used to re-disperse the Janus polymeric particles into aqueous solution. The size of the non-AIE nanomotors was characterized by dynamic light scattering.

Two-Photon (TP) Near-infrared (NIR)-activated motility: The autonomous motion of AIE/Au nanomotors was observed and recorded by a TP-CLSM (Leica TCS SP5X) equipped with a ×40 water immersion microscope objective. AIE/Au nanomotors were detected by the intrinsic AIE fluorescent signal. Movement trajectories were tracked and analyzed by using Image J and Origin software. The TP NIR-infrared activated motility was determined according to previously published procedures.[9] Based on the extracted trajectories, the velocity of NIR propelled AIE/Au nanomotors (V) was calculated following the formula: V = D/t after measuring both the travelled distance (D) and duration time (t). The diffusion coefficient (D) is defined as $D=MSD/i\cdot\Delta t$, where MSD is the mean square displacement (MSD), Δt is the time interval, and *i* is the dimensional index. Here, for the case of two-dimensional analysis from the recorded videos, i is equal to 4. Corresponding mean square displacements (MSD) were then calculated following the reported equation: $MSD= (x(\Delta t)-x(0))^2+(y(\Delta t)-y(0))^2$. [9] The translational diffusion coefficient and rotational diffusion coefficient were calculated with the following equation (1):

$$D_T = \frac{k_B T}{6\pi\eta R} \quad \& \quad D_R = \tau_R^{-1} = \frac{k_B T}{8\pi\eta R^3} \qquad (1)$$

(where η is the viscosity and R the hydrodynamic radius of nanomotor). The theoretical values for particles with an average diameter ~400 nm are $D_T = 1.07$ um^2 s^{-1}, $D_R = 20$ s^{-1}, $\tau_R = 0.05$ s.

Nanosight tracking analysis of motility: Nanoparticle tracking analysis (NTA) was used to analyze the motion behavior of AIE/Au nanomotors, AIE polymersomes (AIE-Ps), and gold shells by using NanoSight S300. Samples were suspended in Milli-Q water to yield an approximate concentration of 10^7 and 10^8 particles per mL. For a typical experiment, 1 mL of sample (ca. 5 µg mL^{-1}) was loaded in the NTA chamber using a syringe. Then, the motion of AIE/Au nanomotors, AIE-polymersomes, and gold nanoshells was recorded for 30 s in triple. A 660 nm DPSS Red Diode Laser was utilized as external laser source to propel the particles. Different laser intensities were used during the experiments, including 0 W (laser off) and 1W (laser on). The same measurement was performed 3 times to ensure reproducibility. The NTA 2.2 software allows the extraction and analysis of the trajectories of single particles. For each group, 30 nanoparticles were tracked for 30 seconds. Their

mean squared displacements (MSD) were calculated following previously published procedures.[10, 11, 12] MSD curves were extracted from the NTA recorded trajectories using the following equation (2):

$$MSD = [\Delta r^2(t)] = [\frac{1}{N}\sum_{i=0}^{N}\left(r_i(t) - r_i(0)\right)^2] \qquad (2)$$

Where r = radius and t = sampling time and MSD (t) = 2dD, where D = diffusion coefficient and d = dimensionality (NTA measurements have dimension d = 2). The equation MSD = (4D)Δt +(v^2)(Δt^2) was used to fit the MSD curves. From the fitting of the MSD curves, the average particle velocity was extracted. According to the particle diffusion coefficient, as described by Golestanian's diffusiophoretic model, a particle undergoing Brownian motion will display a linear MSD over time with the slope determined by the diffusion coefficient $D = K_B\ T/(6\pi\eta R)$. From this model, if the particles are in Brownian motion, the linear component of the MSD, according to the equation MSD = (4D)Δt, can be extracted. Indeed, in the absence of NIR light or 405 nm light irradiation, a linear relation between MSD and time was observed (Figure 3e and 3h in main text, and Supplementary Fig. 41). The same linear relationship was also observed when control particles were exposed to light irradiation (Supplementary Fig. 42, 43, 46 and 47). In the presence of NIR light or 405 nm light, the gold shell coated asymmetric AIE/Au nanomotors displayed observable autonomous motion(In main text Figure 3e and 3h, and Supplementary Fig. 41), the MSD curves (in the presence of light Δt > r) displayed a parabolic fit.

Toxicity studies: HeLa cells were cultured in DMEM medium containing 10 % FBS, 1 % penicillin/streptomycin (complete DMEM) in 5 % CO_2 at 37 °C. The relative cell viability was evaluated *in vitro* by an MTT assay. The cells were seeded in 96-well plates at a density of 5 × 10^3 cells per well in 100 μL complete DMEM medium and cultured for 24 h at 37 °C. Subsequently, the cells were incubated with the corresponding nanoparticles (AIE-polymersomes or AIE/Au nanomotors) at different concentrations for 24 h. The cells were washed and fresh medium containing MTT was added into each plate. The cells were incubated for another 4 h. After removing the medium containing MTT, dimethyl sulfoxide (100 μL) was added to each well to dissolve the formazan crystals. Finally, the plate was gently shaken for 5 min and the absorbance at 490 nm was recorded with a micro-plate reader.

Internalization of AIE-polymersomes within cells: HeLa cells were cultured in DMEM medium containing 10 % FBS, 1 % penicillin/streptomycin (complete DMEM) in 5 % CO_2 at 37 °C. HeLa cells were seeded in a μ-slide 8 well plate for 24 h, and then the medium was refreshed. Next the cells were incubated with AIE-polymersomes (200 μg ml^{-1}) for 2, 6, and 24 h, washed and stained with Hoechst 33342 for 10 min. Then, the fluorescence images of the cells were captured using a Leica TCS 264 SP5X system.

ROS production of AIE-polymersomes within cells: HeLa cells were cultured in DMEM medium containing 10 % FBS, 1 % penicillin/streptomycin (complete DMEM) in 5 % CO_2 at 37 °C. HeLa cells were seeded in a μ-slide 8 well plate for 24 h, and then the medium was refreshed. Next, the cells

were incubated with AIE-polymersomes (200 µg ml^{-1}) for 24 h and washed. The cells were subsequently loaded with CM-H2DCFDA for 0.5 h and stained with Hoechst 33342 for 10 minutes. Then the cells were washed with PBS for 3 times. After that, the cells were subjected to a two-photon confocal NIR laser and the fluorescence images of the cells were captured using a Leica TCS 264 SP5X system.

Cell membrane interaction of AIE/Au nanomotors: HeLa cells were cultured in DMEM medium containing 10 % FBS, 1 % penicillin/streptomycin (complete DMEM) in 5 % CO_2 at 37 °C. HeLa cells were seeded in a µ-slide 8 well plate for 24 h, and then the medium was refreshed. The cells were stained with wheat germ agglutinin Alexa Fluor-TM 488 conjugate and Hoechst 33342 to show the cell membrane, as well as with propidium iodide (PI) to show the real-time enhanced permeability of cell membranes. The cells were washed with PBS twice. Thereafter, the cells were treated with AIE/Au nanomotors (25 µg ml^{-1}). Immediately, cells were subjected to a two-photon confocal NIR laser and the fluorescence images of the cells were captured using a Leica TCS 264 SP5X system.

ROS production of AIE/Au nanomotors within cells: HeLa cells were cultured in DMEM medium containing 10 % FBS, 1 % penicillin/streptomycin (complete DMEM) in 5 % CO_2 at 37 °C. HeLa cells were seeded in a µ-slide 8 well plate for 24 h, and then the medium was refreshed. The cells were subsequently loaded with CM-H2DCF for 0.5 h and stained with Hoechst 33342 for 10 minutes. Then the cells were washed and treated with AIE/Au nanomotors (25 µg ml^{-1}). Immediately, the cells were subjected to a two-photon confocal NIR laser and the fluorescence images of the cells were captured using a Leica TCS 264 SP5X system.

Cell necrosis induced by AIE/Au nanomotors: HeLa cells were cultured in DMEM medium containing 10 % FBS, 1 % penicillin/streptomycin (complete DMEM) in 5 % CO_2 at 37 °C. HeLa cells were seeded in a µ-slide 8 well plate for 24 h, and then the medium was refreshed. Then, the cells were stained with Hoechst 33342 for 10 minutes. Thereafter, the cells were washed and incubated with calcein for live cell staining, and PI for dead cell staining for 10 min. Next, the cells were treated with AIE/Au nanomotors (25 µg ml^{-1}). Immediately, the cells were subjected to a two-photon confocal NIR laser and the fluorescence images of the cells were captured using a Leica TCS 264 SP5X system.

Supplementary Figures and Tables

Supplementary Figure 1. ^{1}H NMR spectrum of **AIE-Br** in $CDCl_3$.

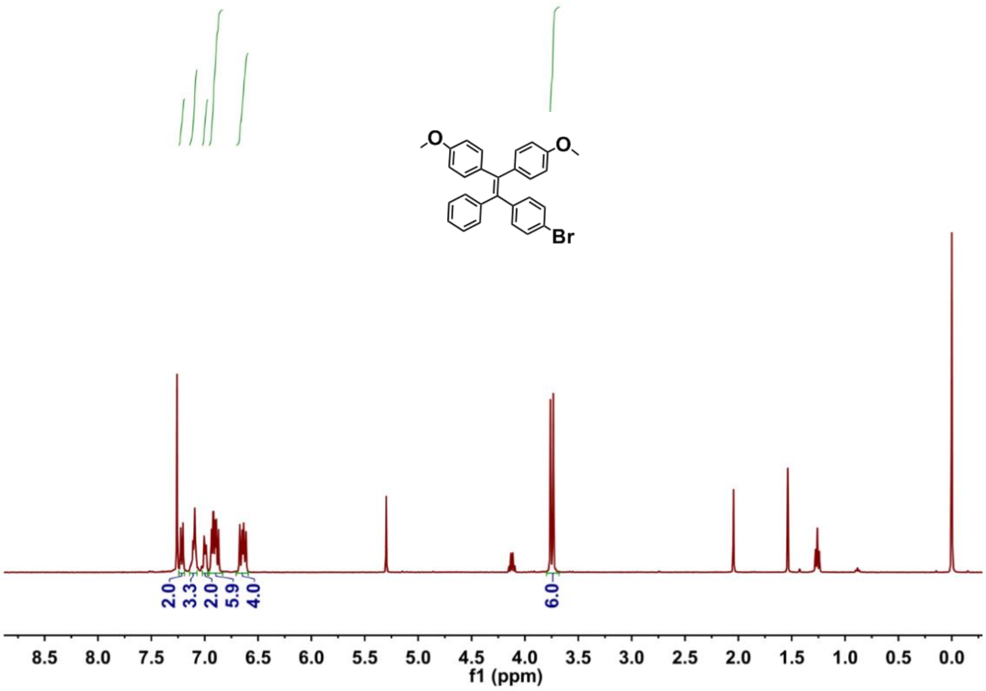

Supplementary Figure 2. ^{1}H NMR spectrum of **AIE-OH** in $CDCl_3$.

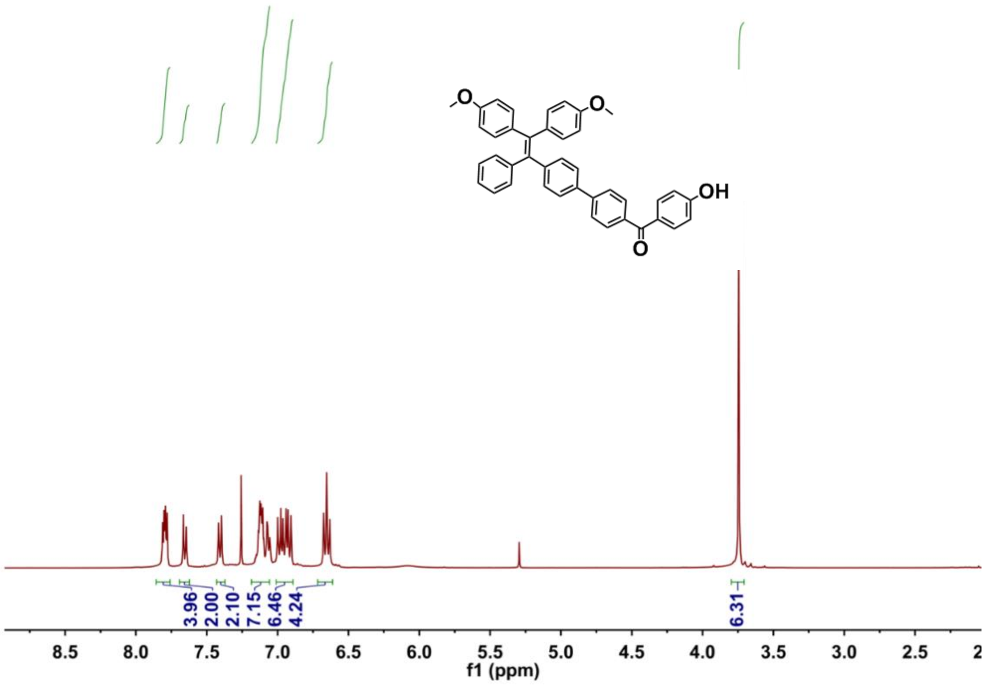

Supplementary Figure 3. ^{13}C NMR spectrum of **AIE-OH** in $CDCl_3$.

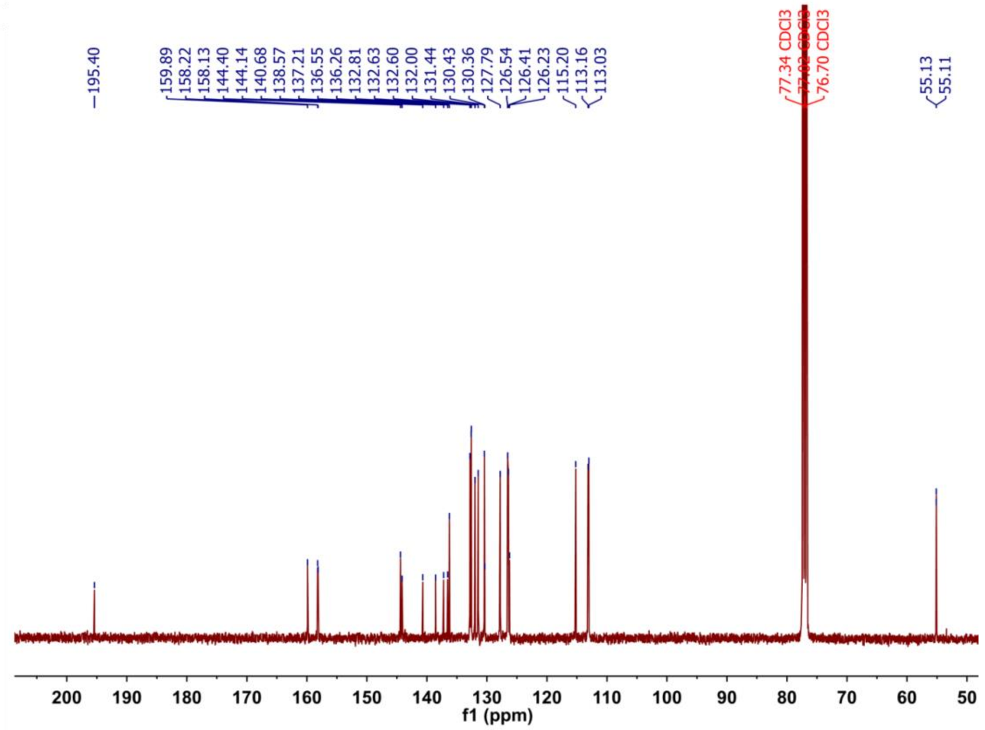

Supplementary Figure 4. MALDI TOF spectrum of **AIE-OH** in THF.

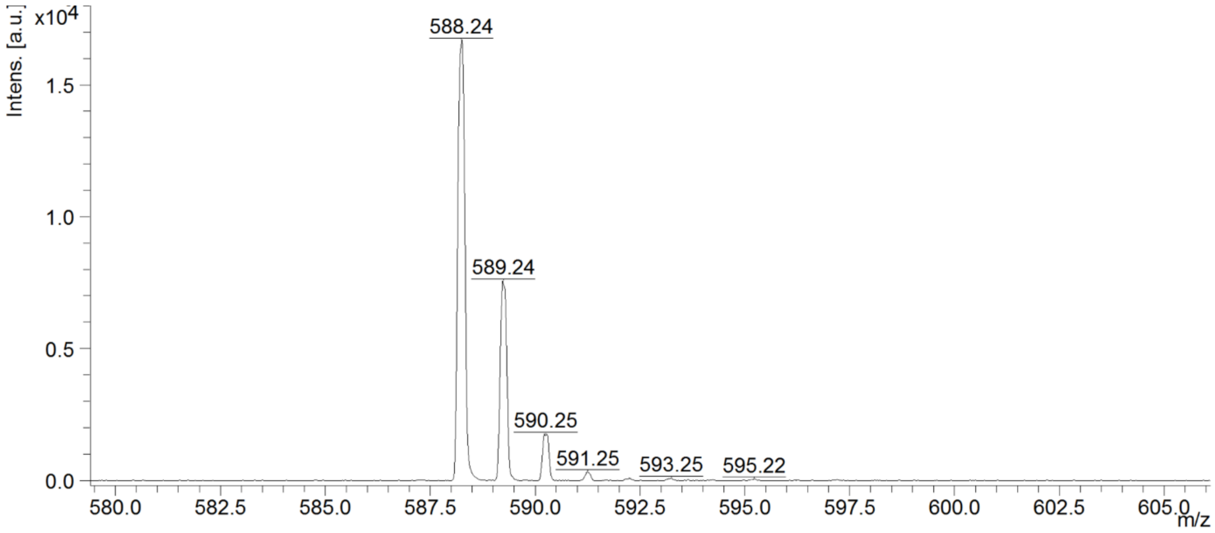

Supplementary Figure 5. ^{1}H NMR spectrum of **AIE-CN-OH** in $CDCl_3$.

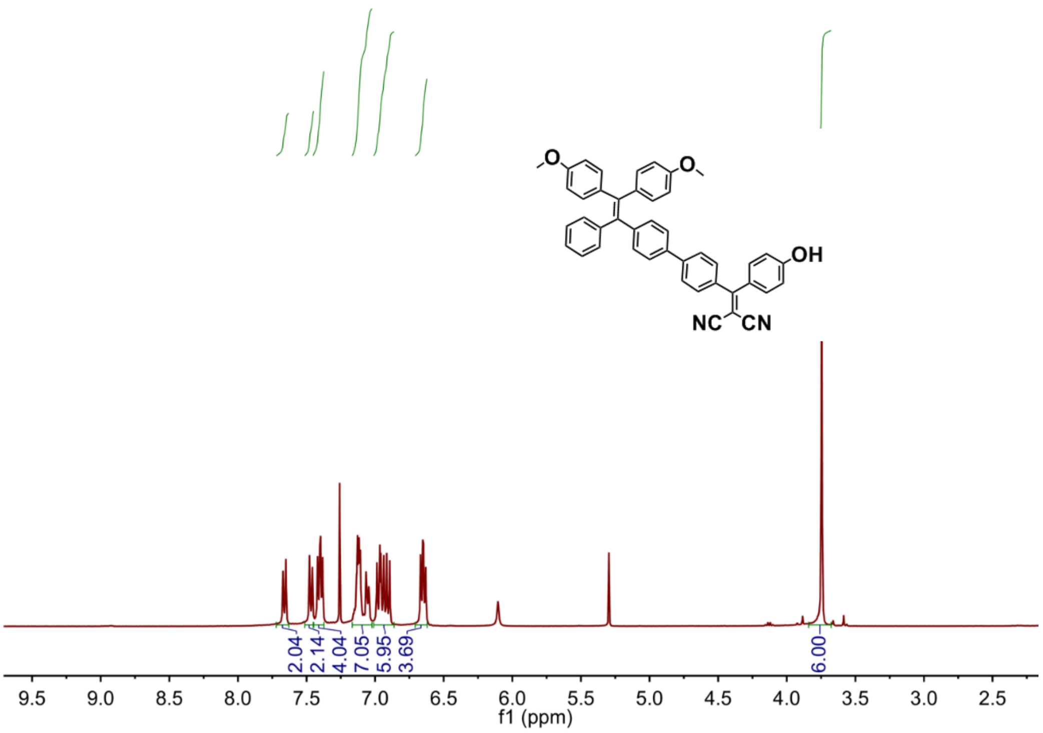

Supplementary Figure 6. ^{13}C NMR spectrum of **AIE-CN-OH** in $CDCl_3$

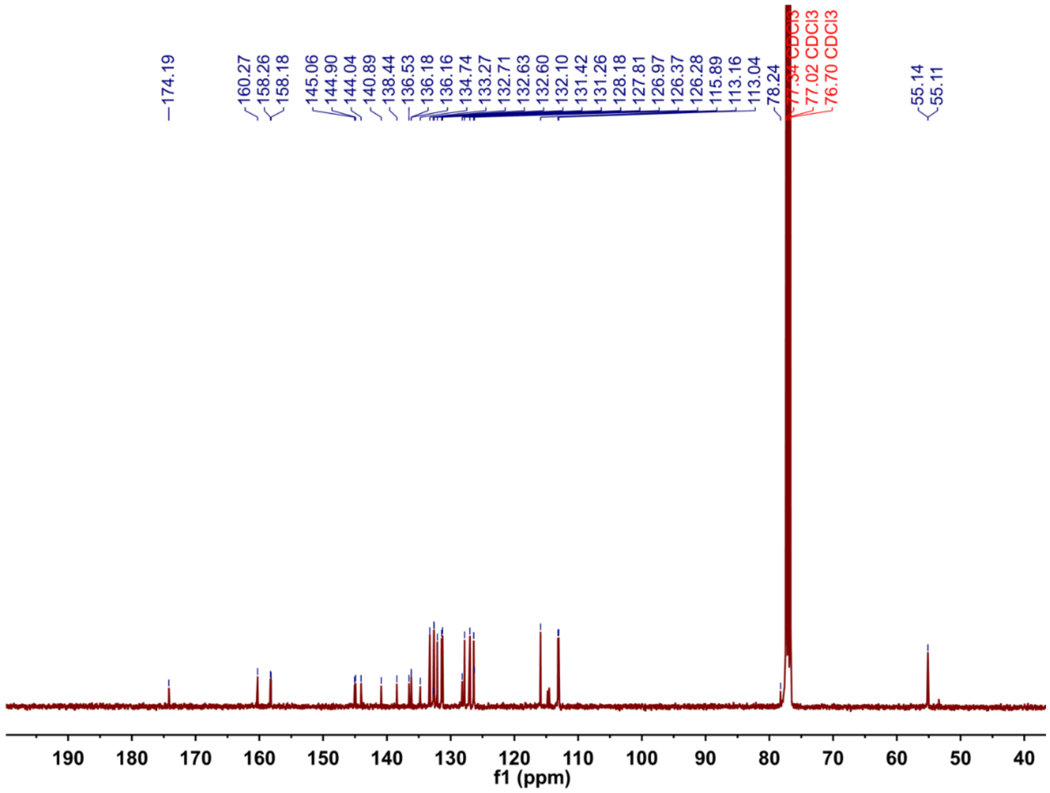

Supplementary Figure 7. MALDI TOF spectrum of **AIE-CN-OH** in THF.

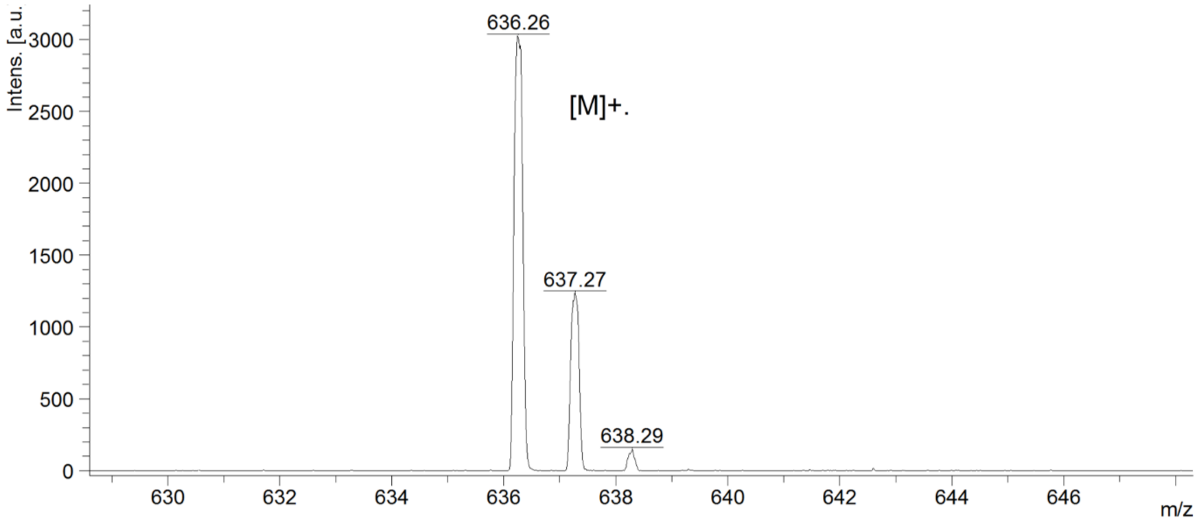

Supplementary Figure 8. ^{1}H NMR spectrum of **AIE-Boc** in $CDCl_3$.

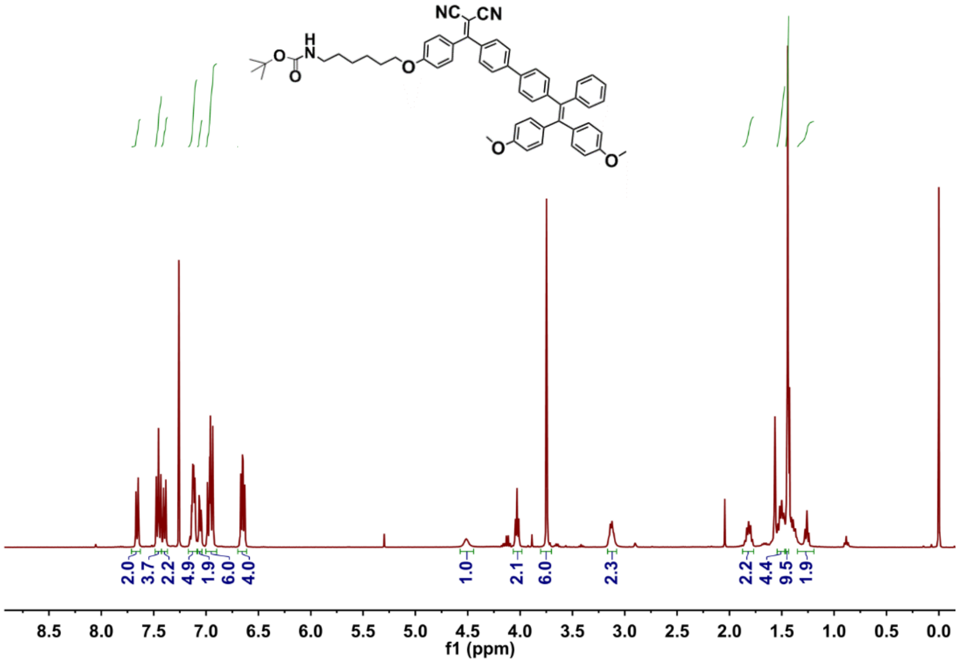

Supplementary Figure 9. ^{13}C NMR spectrum of **AIE-Boc** in $CDCl_3$.

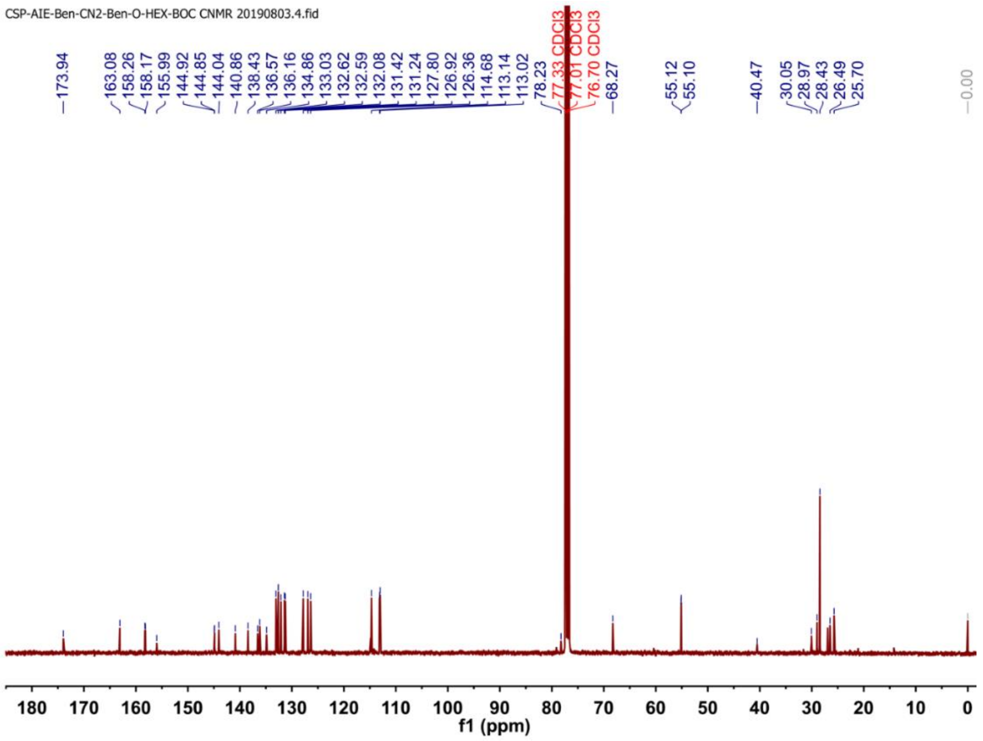

Supplementary Figure 10. MALDI TOF spectrum of **AIE-Boc** in THF.

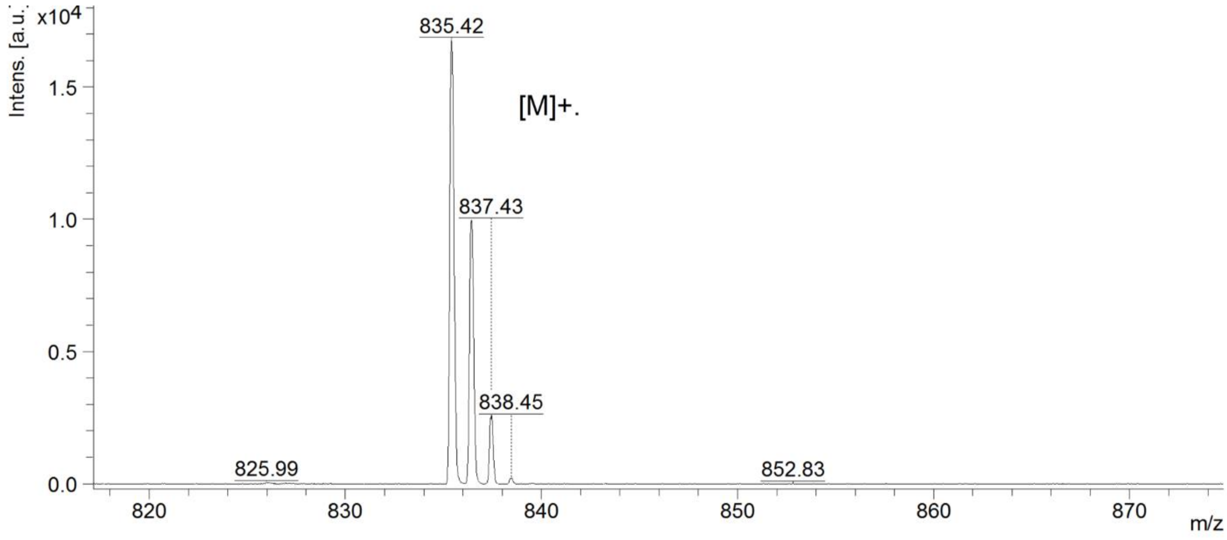

Supplementary Figure 11. ^{1}H NMR spectrum of **AIE-NH2** in $CDCl_3$.

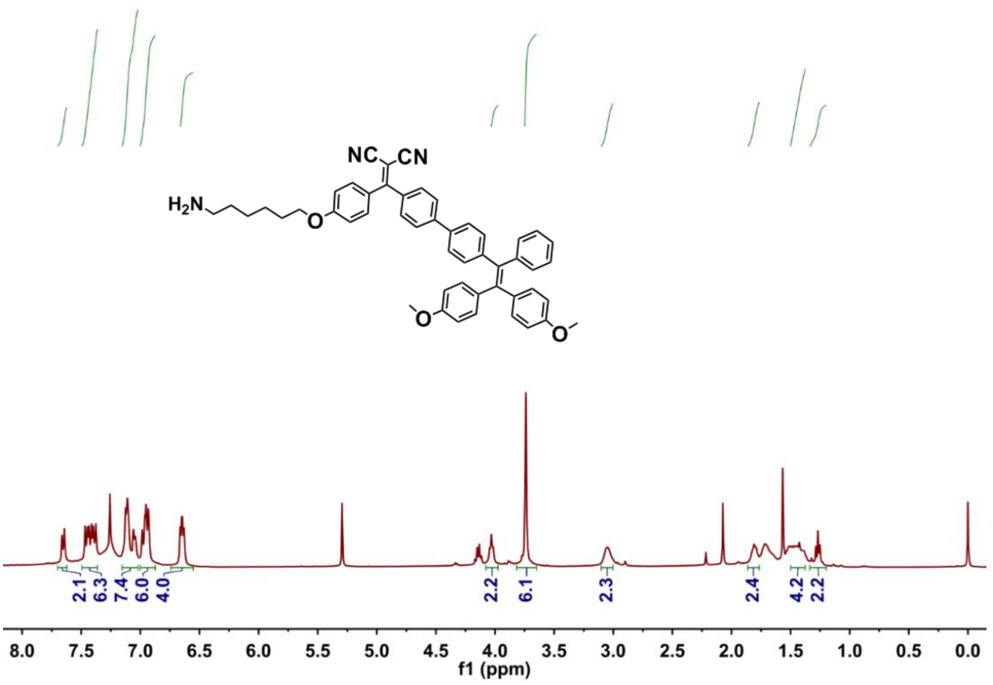

Supplementary Figure 12. ^{13}C NMR spectrum of **AIE-NH2** in $CDCl_3$.

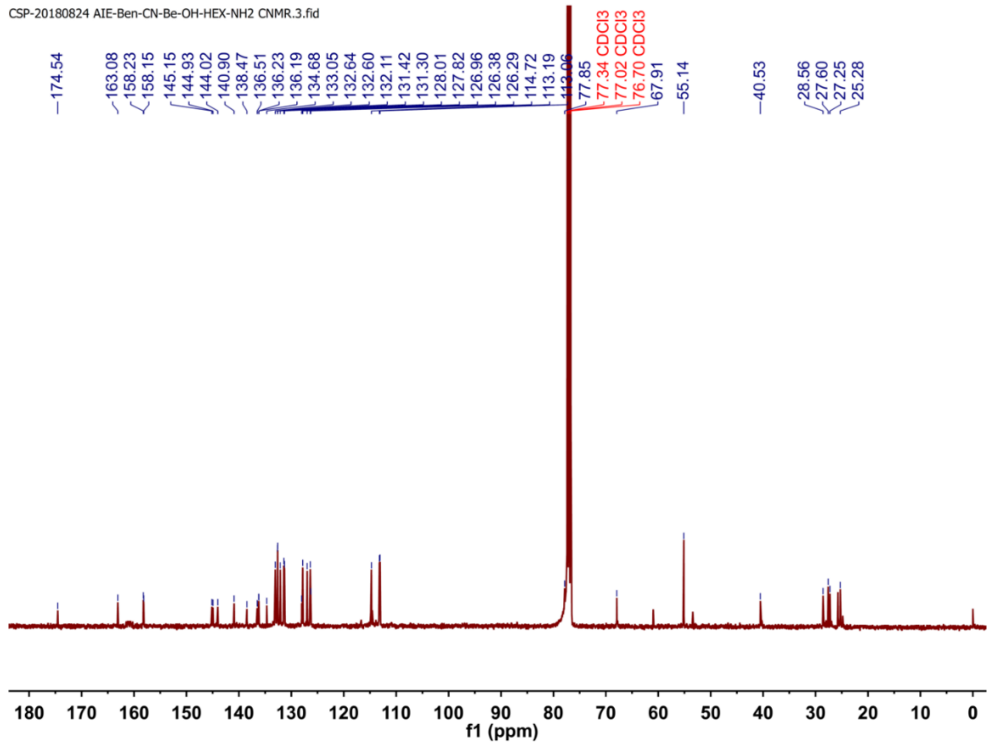

Supplementary Figure 13. MALDI TOF spectrum of **AIE-NH_2** in THF.

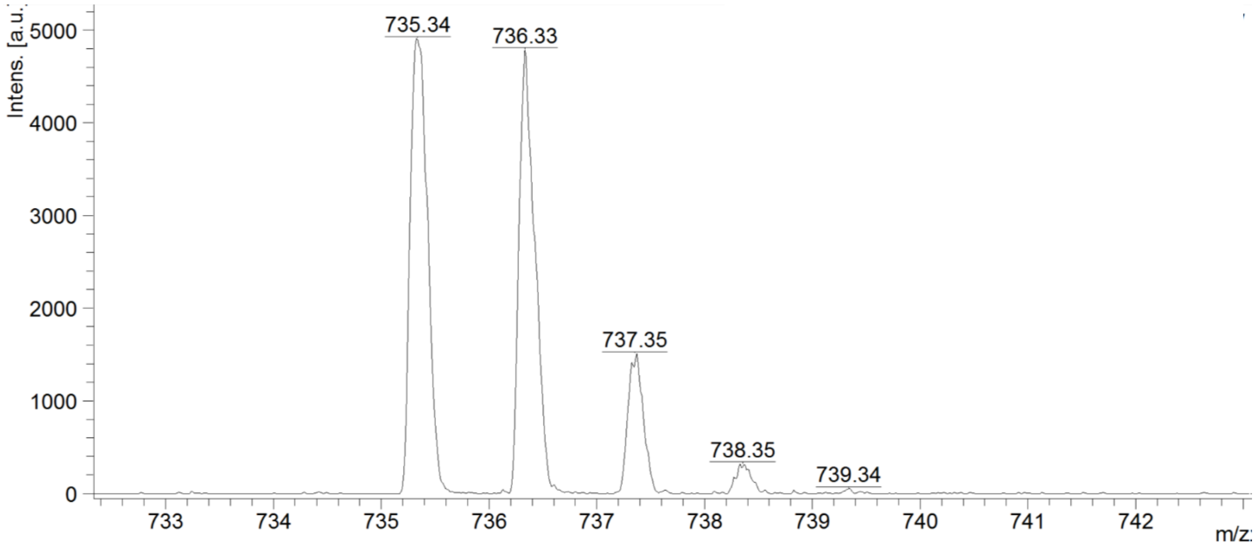

Supplementary Figure 14. ^{1}H NMR spectrum of **PEG_{44}-$PTMCP_5$**

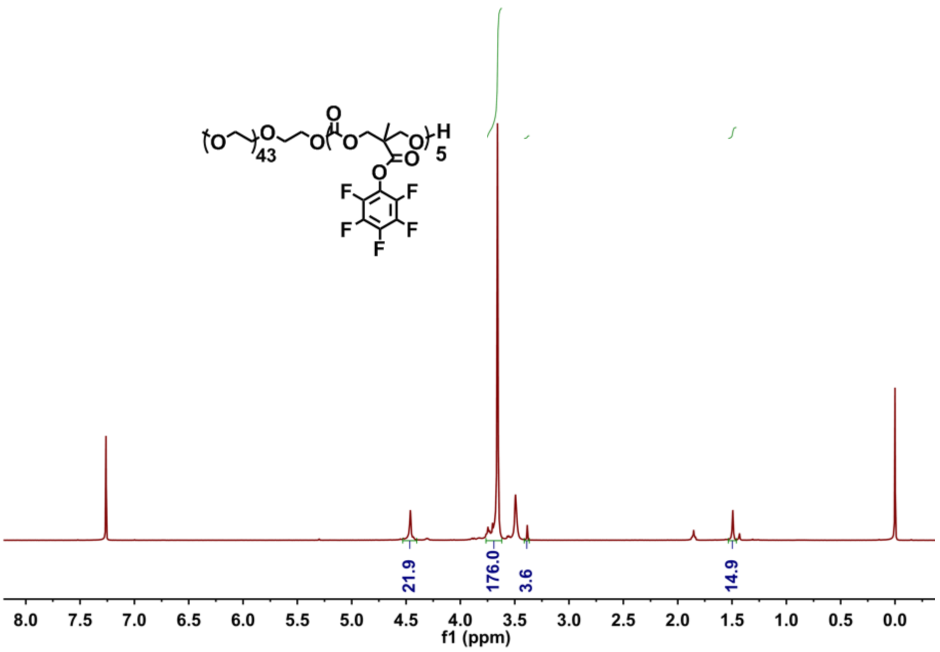

Supplementary Figure 15. ^{1}H NMR spectrum of **PEG44-PTMCP8**

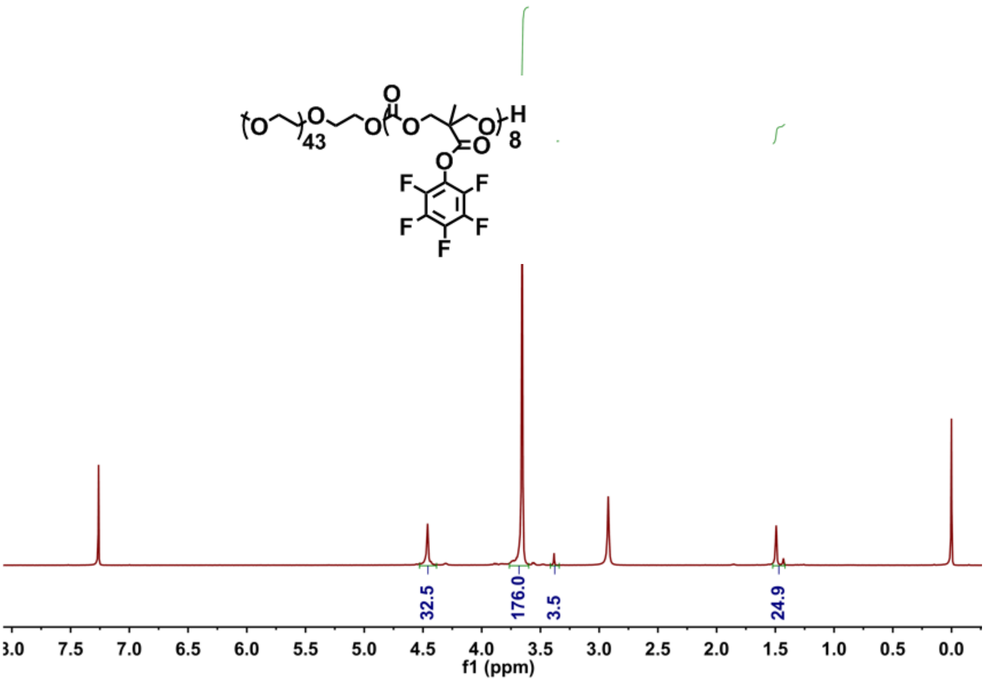

Supplementary Figure 16. ^{1}H NMR spectrum of **PEG44-PTMCP14**

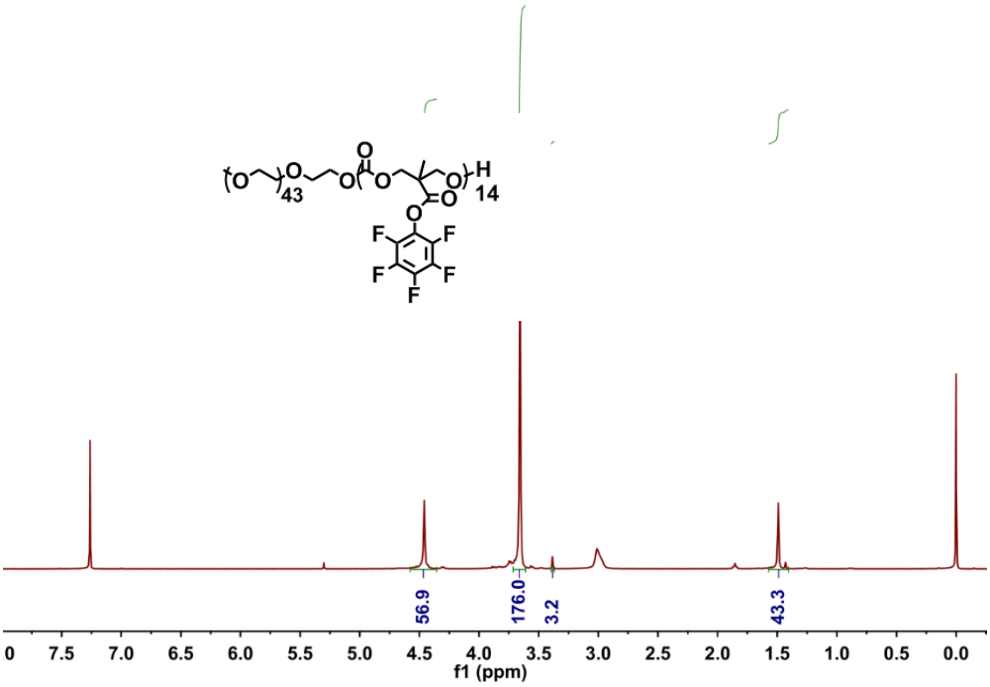

Supplementary Figure 17. ^{1}H NMR spectrum of **PEG$_{44}$-PTMCP$_{22}$**

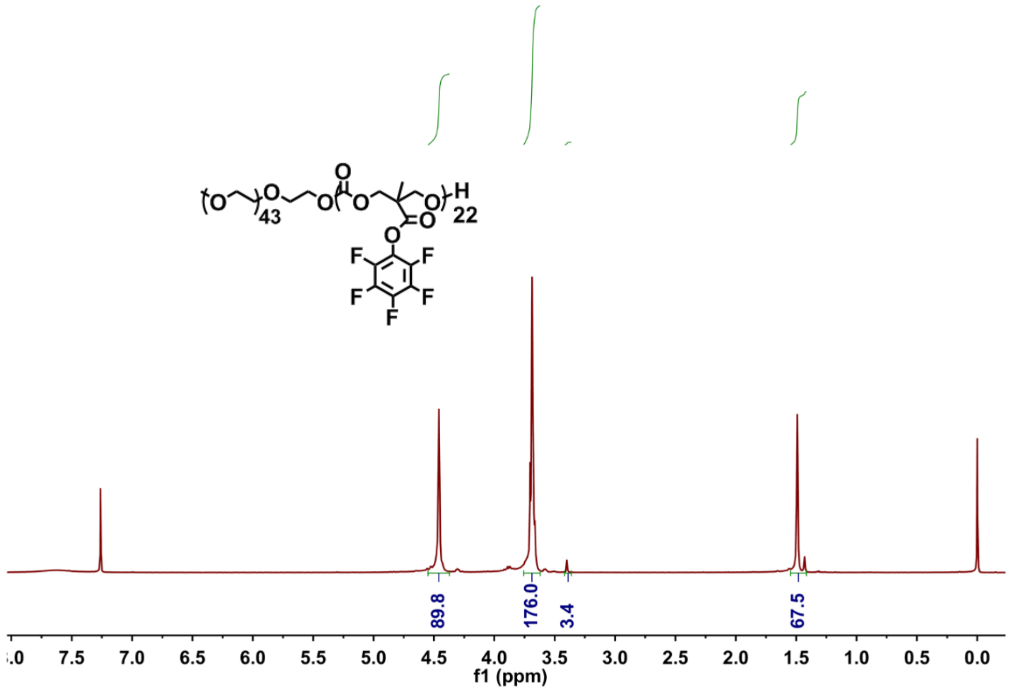

Supplementary Figure 18. Magnified GPC traces for **PEG$_{44}$-PTMCP$_5$** (black), **PEG$_{44}$-PTMCP$_8$** (red), **PEG$_{44}$-PTMCP$_{14}$** (blue), and **PEG$_{44}$-PTMCP$_{22}$** (green).

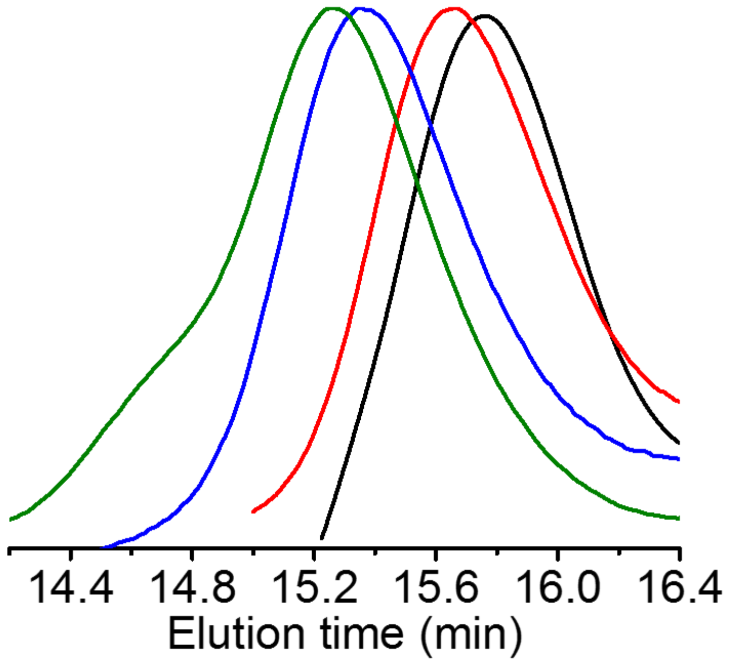

Supplementary Figure 19. ^{1}H NMR spectrum and GPC trace of **PEG$_{44}$-P(AIE)$_{5}$**.

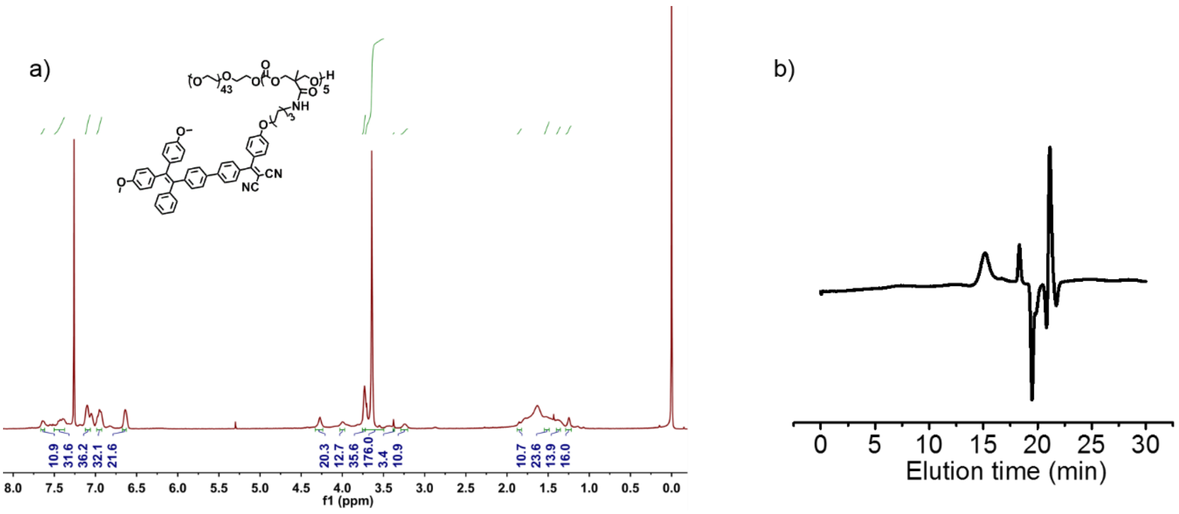

Supplementary Figure 20. ^{1}H NMR spectrum and GPC trace of **PEG$_{44}$-P(AIE)$_{8}$**.

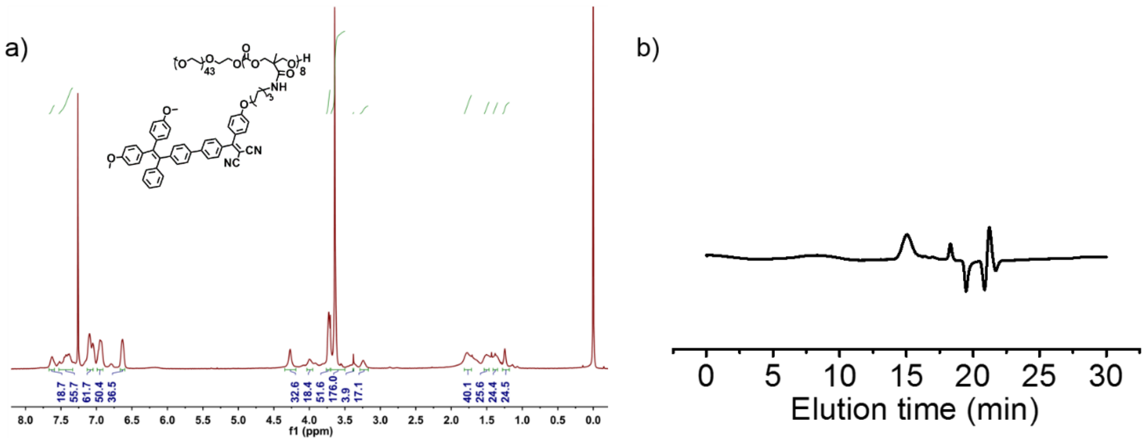

Supplementary Figure 21. ^{1}H NMR spectrum and GPC trace of **PEG44-P(AIE)14**.

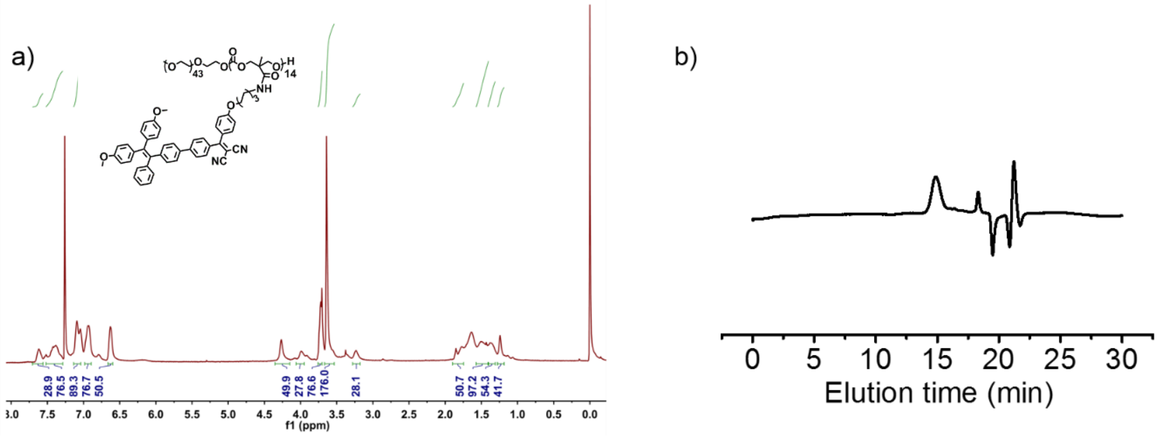

Supplementary Figure 22. ^{1}H NMR spectrum and GPC trace of **PEG44-P(AIE)22**.

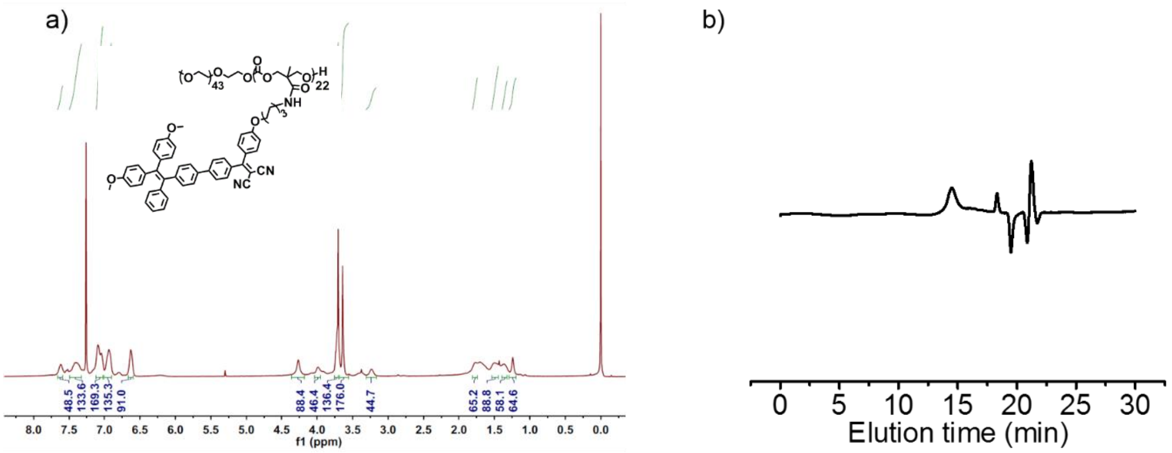

Supplementary Figure 23. Magnified GPC traces for **PEG_{44}-P(AIE)$_5$** (black curve), **PEG_{44}-P(AIE)$_8$** (red curve), **PEG_{44}-P(AIE)$_{14}$** (blue curve), and **PEG_{44}-P(AIE)$_{22}$** (green curve).

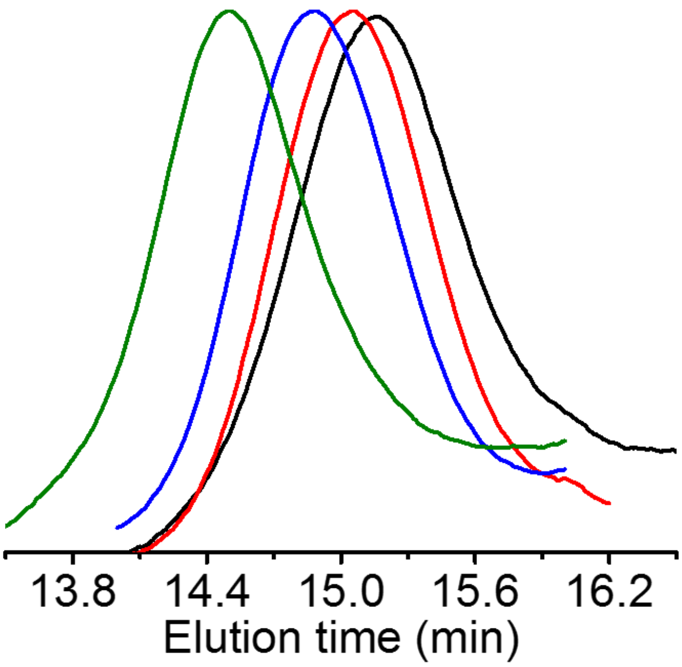

Supplementary Figure 24. Representative ^{19}F NMR of purified **PEG_{44}-$PTMCP_5$** (top) and **PEG_{44}-P(AIE)$_5$** (down), indicating full conversion of the TMCP group after reaction with **AIE-NH_2**.

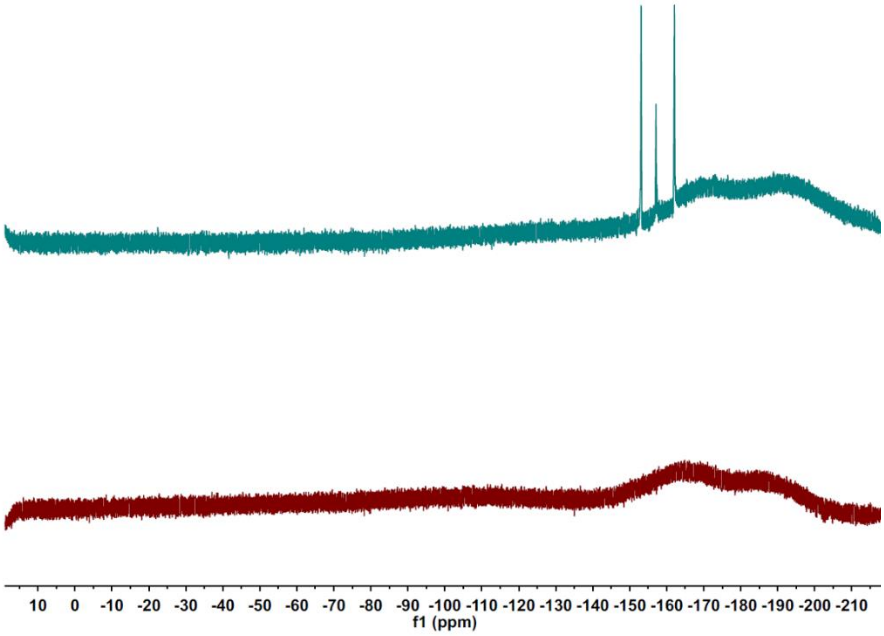

Supplementary Table 1. GPC analysis of all block copolymers synthesized and used in this work.

Composition	M_n / kDa	GPC
		Đ
PEG_{44}-$PTMCP_5$	3.8	1.08
PEG_{44}-$PTMCP_8$	4.3	1.10
PEG_{44}-$PTMCP_{14}$	6.3	1.08
PEG_{44}-$PTMCP_{22}$	7.7	1.13
PEG_{44}-$P(AIE)_5$	8.2	1.11
PEG_{44}-$P(AIE)_8$	9.4	1.10
PEG_{44}-$P(AIE)_{14}$	11.0	1.09
PEG_{44}-$P(AIE)_{22}$	14.9	1.11
PEG_{44}-$PDLLA_{115}$*	17.8	1.08

* This block co-polymer was used as the non-AIE polymer control.

Supplementary Figure 25. (a) The chemical structure of the PEG_{44}-$P(AIE)_n$ block copolymers and their self-assembly via a typical solvent switch method, whereby a solution of block copolymer in organic solvent (THF) was diluted by water (up to 50% v/v at 0.25mL h^{-1}). (b) Representative cryo-electron micrographs of polymersomes formed by the solvent switch method (THF and water) from PEG_{44}-b-$P(AIE)_5$. The 'zoom in' displays the schematic molecular organization of the polymersome membrane.

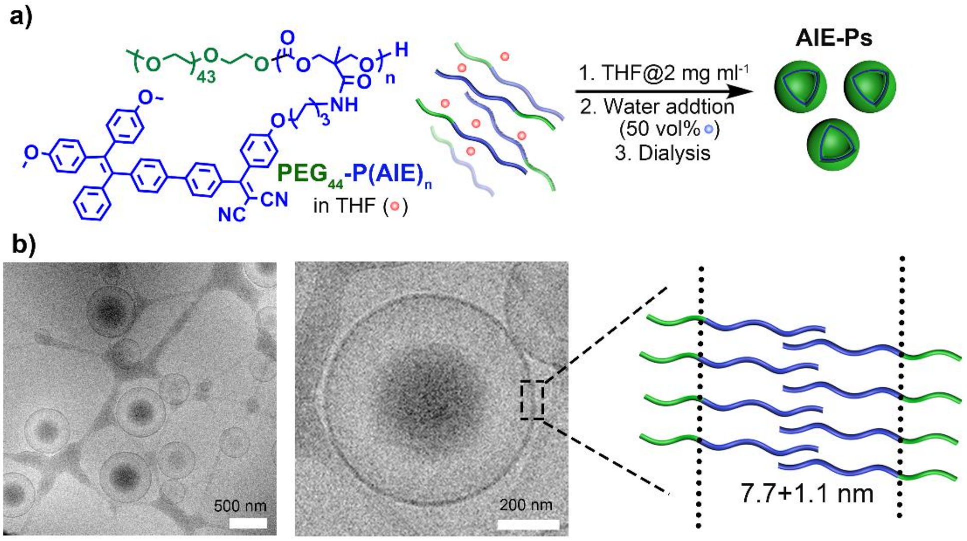

Supplementary Figure 26. Self-assembly of PEG_{44}-$P(AIE)_5$ copolymers following the solvent switch method, whereby a solution of block copolymer in organic solvent (THF) was diluted by water (up to 50% v/v at 0.25 mL h^{-1} (as described in the methods section) with all measurements performed after extensive dialysis against water. (a) DLS intensity plot (with correlation coefficient as inset). (b) SEM image. (c) TEM image. (d) cryo-TEM image.

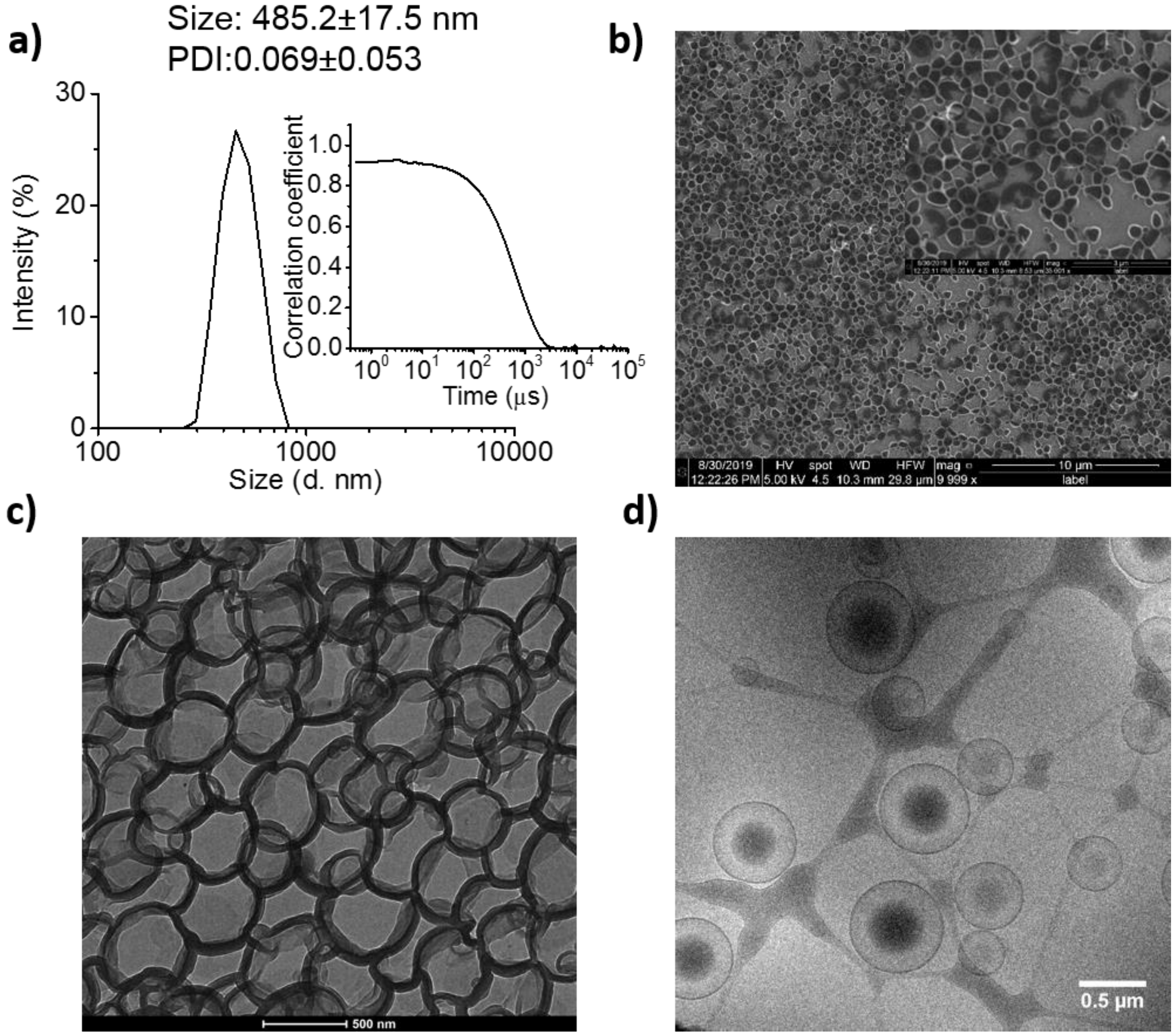

Supplementary Figure 27. Self-assembly of PEG_{44}-$P(AIE)_8$ copolymers following the solvent switch method (as described in the method section) with all measurements performed after extensive dialysis against water. (a) DLS curve (with correlation coefficient as inset). (b) SEM image. (c) TEM image. (d) cryo-TEM image.

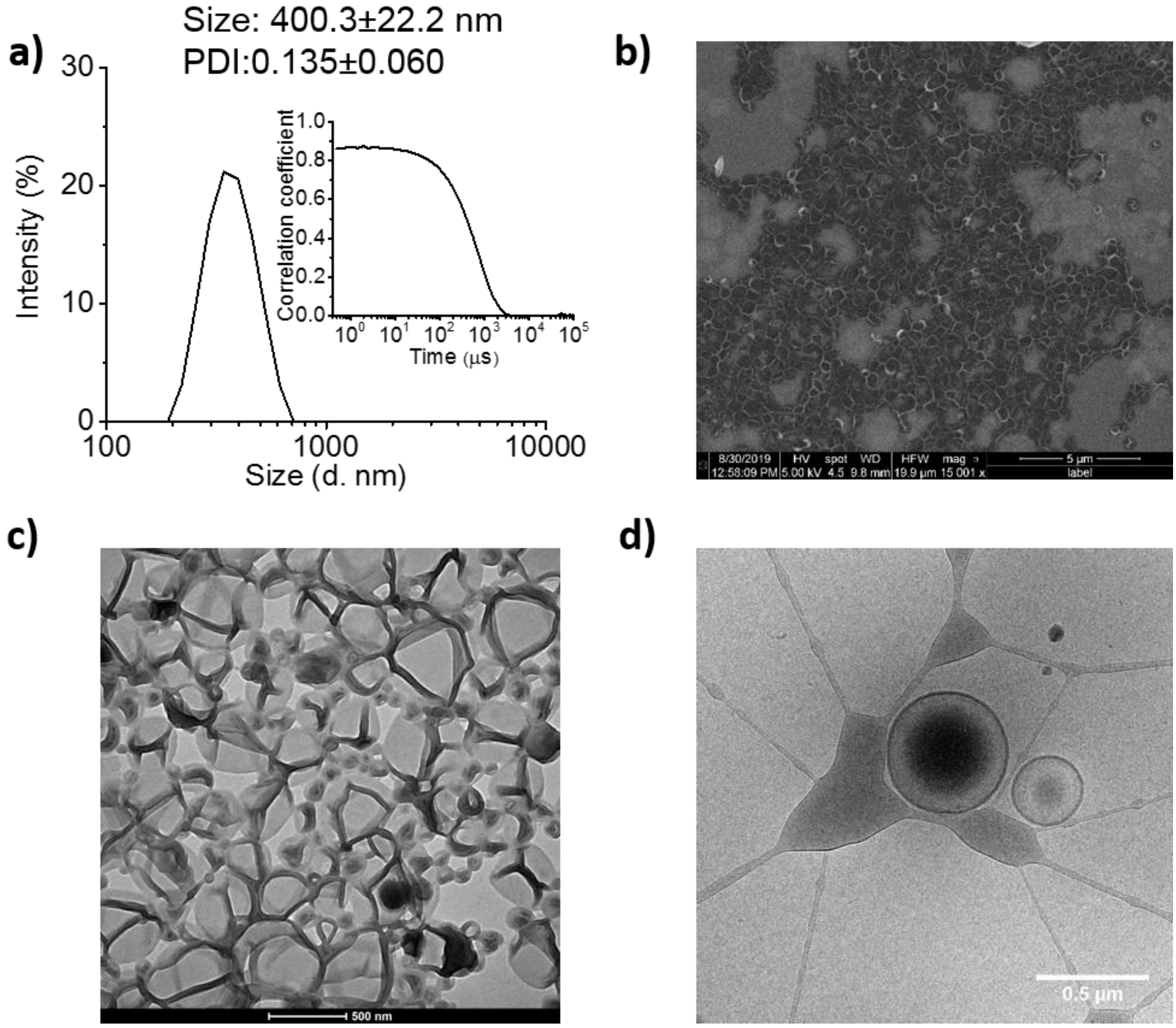

Supplementary Figure 28. Self-assembly of PEG_{44}-$P(AIE)_{22}$ copolymers following a fast nanoprecipitation method (the THF solution was added instantaneously to water instead of dropwise) with all measurements performed after extensive dialysis against water. (a) DLS curve (with correlation coefficient as inset). (b) SEM image. (c) TEM image. (d) cryo-TEM image.

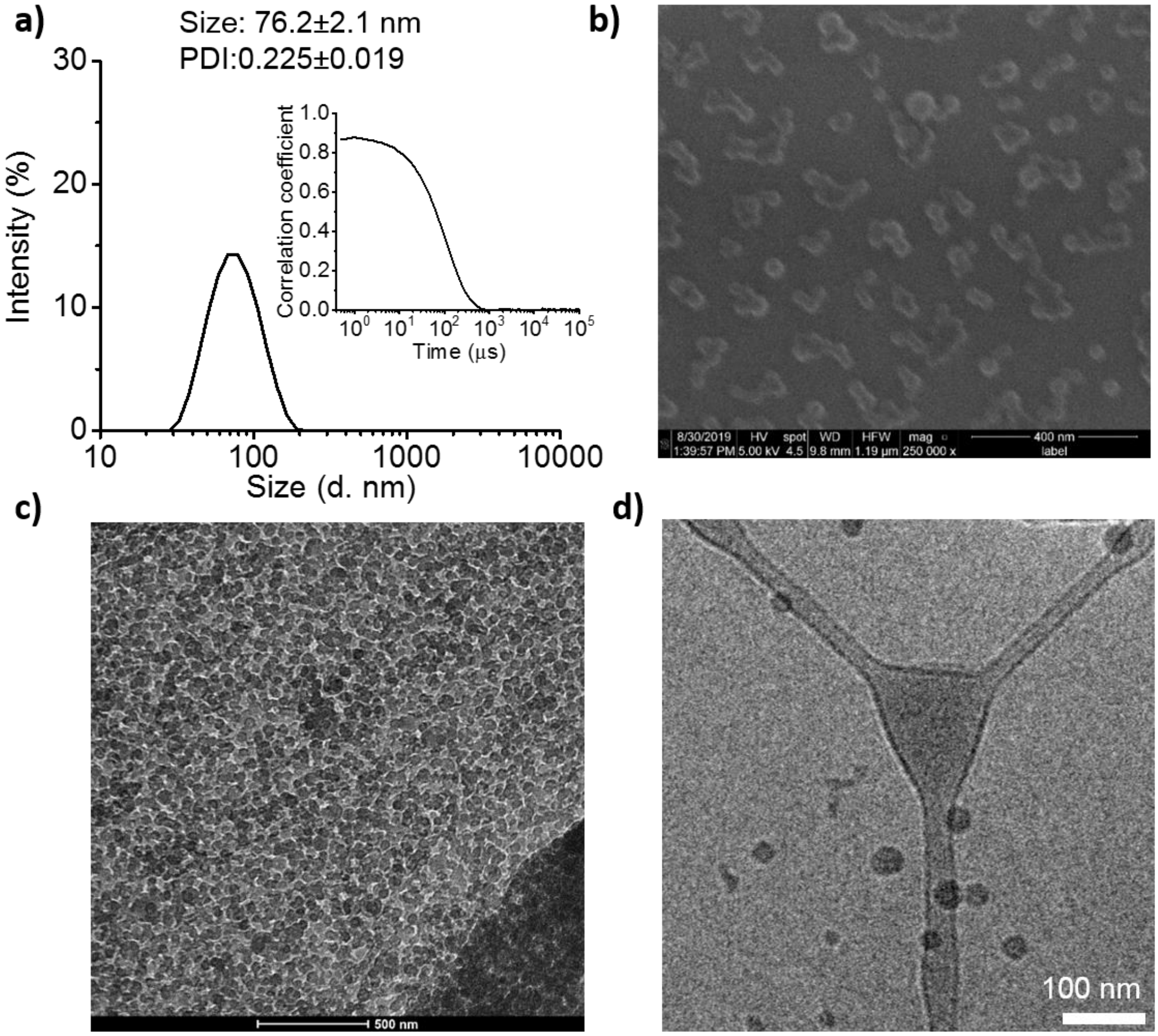

Supplementary Table 2. Characterization of PEG_{44}-$P(AIE)_n$ diblock copolymers and their respective assemblies.

[*]*: membrane thickness as determined from analysis of cryo-TEM images.*

[**]*: nanoparticles from* PEG_{44}-$P(AIE)_{22}$ were formulated by a fast nanoprecipitation method

Block co-polymer	PEG_{44}-$P(AIE)_5$	PEG_{44}-$P(AIE)_8$	PEG_{44}-$P(AIE)_{14}$	PEG_{44}-$P(AIE)_{22}$
DP of AIE block	5	8	14	22
f_{PEG} (wt%)	31	22	14	9
f_{AIE} (wt%)	50	56	62	65
D_h (nm)	505 nm	422 nm	346 nm	76 nm[**]
M (nm)[*]	7.7±1.1 nm	10.7±2.3 nm	13.8±1.8 nm	-

Supplementary Figure 29. (a) UV-Vis spectra of PEG_{44}-$P(AIE)_{14}$ copolymers in a mixture of DMSO and water. (b) Fluorescence spectra of PEG_{44}-$P(AIE)_{14}$ in different mixtures of DMSO and water (Excitation wavelength = 373 nm). (c) Photographs of PEG_{44}-$P(AIE)_{14}$ solutions at different water content (water and DMSO solution) illuminated by a lab UV-lamp.

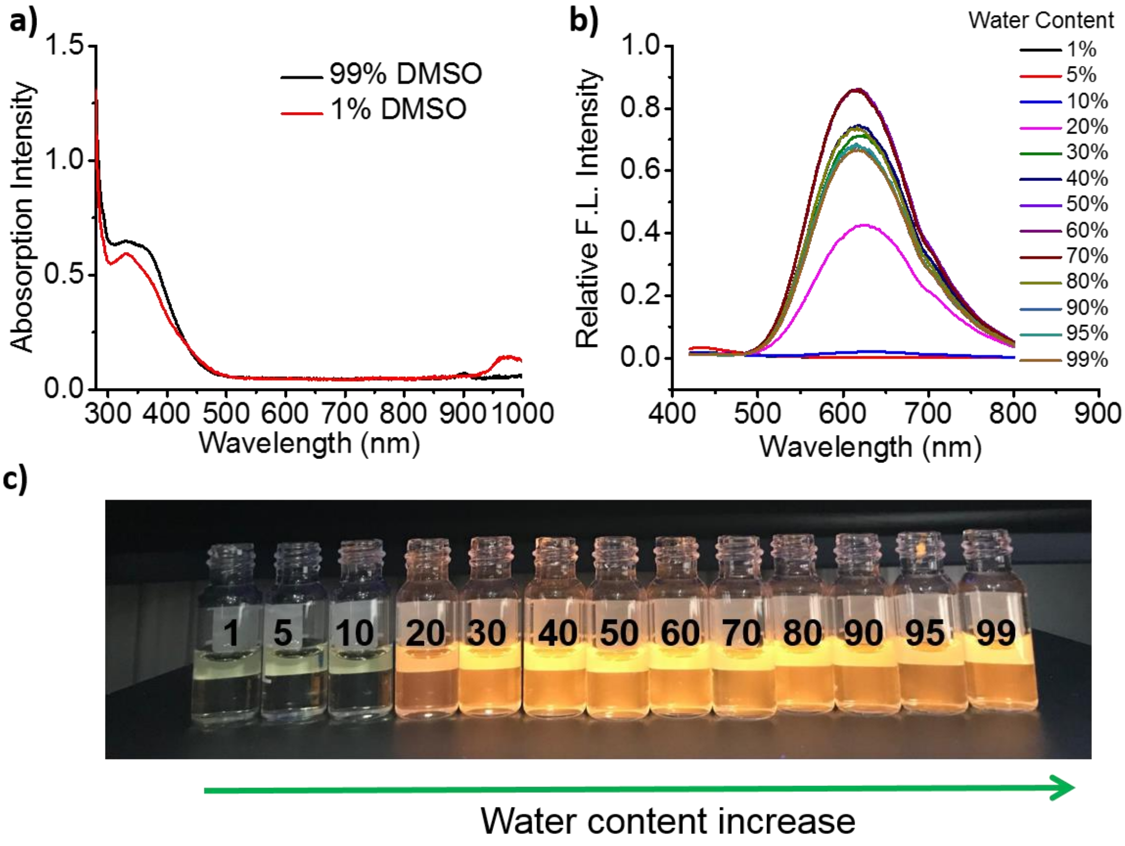

Supplementary Figure 30. (a) DLS curve and (b) correlation curve of AIE-polymersomes and AIE/Au nanomotors

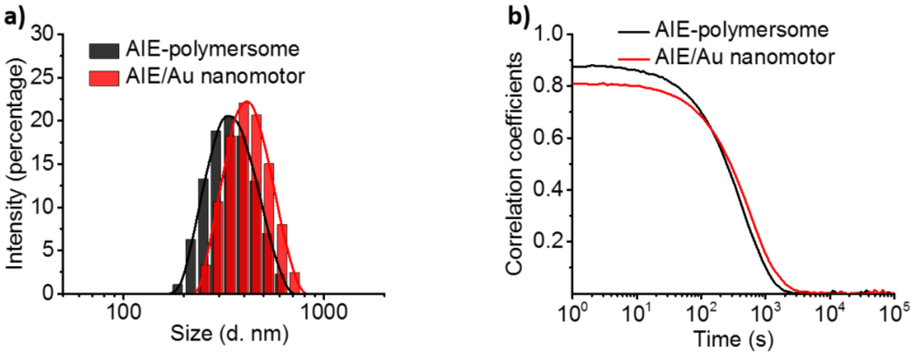

Supplementary Figure 31. Comparison between AIE-polymersome and AIE/Au nanomotor morphologies. (a) Representative scanning electron microscopy (SEM) image of uniform AIE-polymersomes (with a typical single particle as inset, scale bar=200 nm); (b) Intensity profile of a single particle in a. (c) Transmission electron microscopy (TEM) image of AIE-polymersomes; (d) Representative scanning electron microscopy (SEM) image of uniform AIE/Au nanomotors (with single particles as inset), (e) Intensity profile of a single particle in d. (f) Transmission electron microscopy (TEM) image of AIE/Au nanomotors.

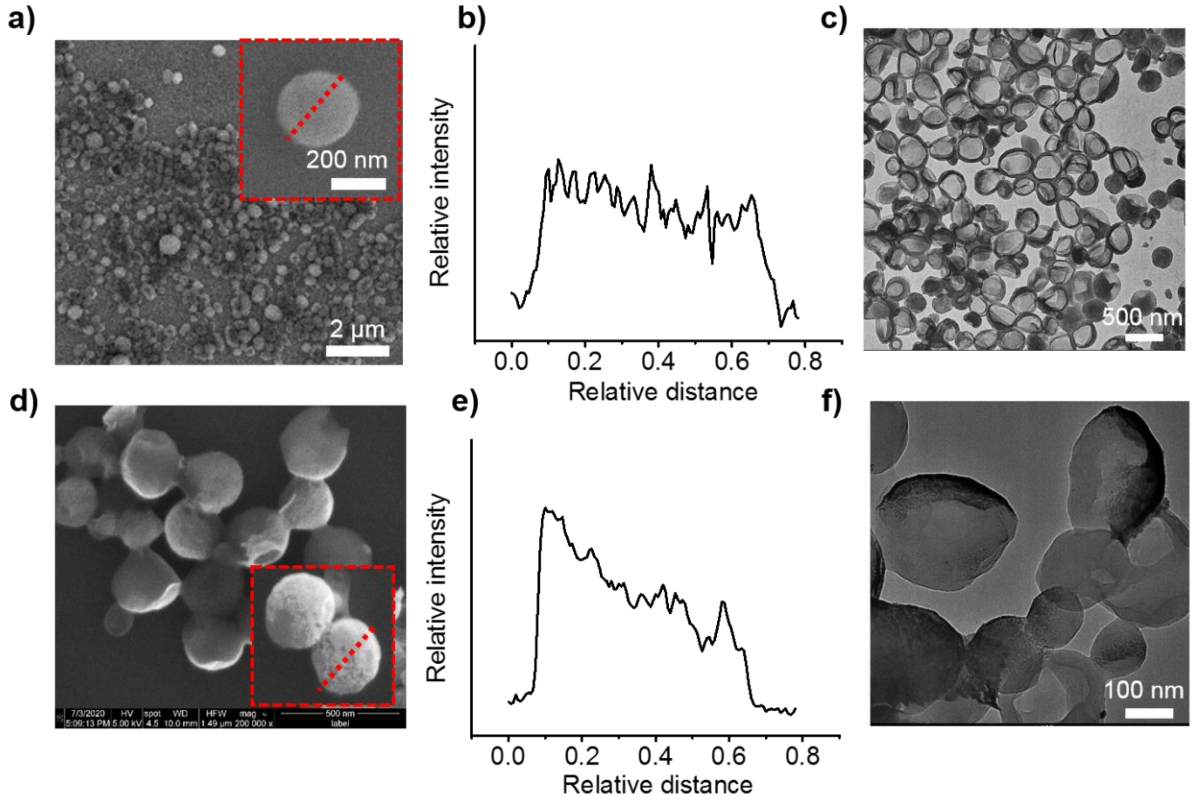

Supplementary Figure 32. Transmission electron microscopy (TEM) images of AIE/Au nanomotors, demonstrating uniformity after re-dispersing into Milli-Q water.

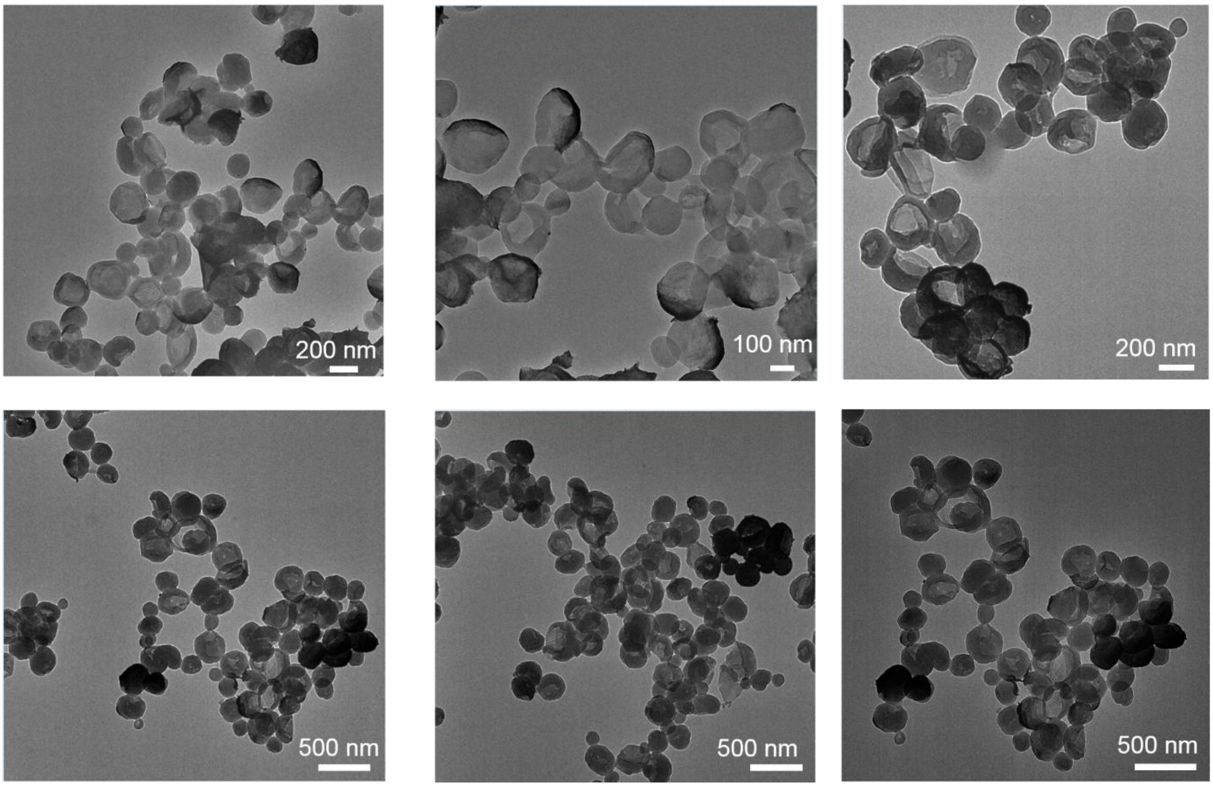

Supplementary Figure 33. Energy dispersive X-ray analysis (EDX) analysis of AIE/Au nanomotors confirming successful gold shell coating.

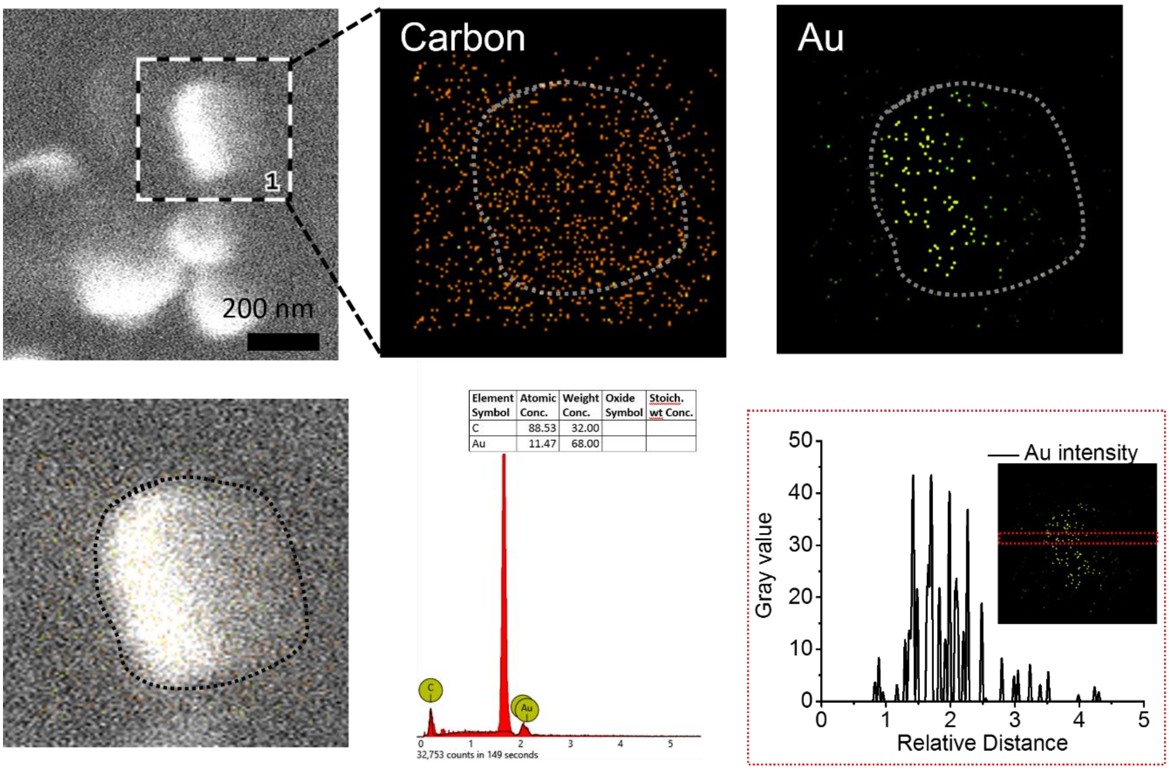

Supplementary Figure 34. Shape recovery of the dried AIE/Au nanomotors after being dispersed in water for different period of time. (a) Size measurement by DLS after rehydration for 3 days; (b) cryo-TEM images of the dried nanomotors after being redispersed in MQ water for 18 h; (c) cryo-TEM images of these nanomotors after rehydration for 3 days. Nearly spherical polymersomes from PEG_{44}-$PAIE_{14}$ were observed, indicating the robustness of the vesicular polymersomes structure. Scale bar=200 nm

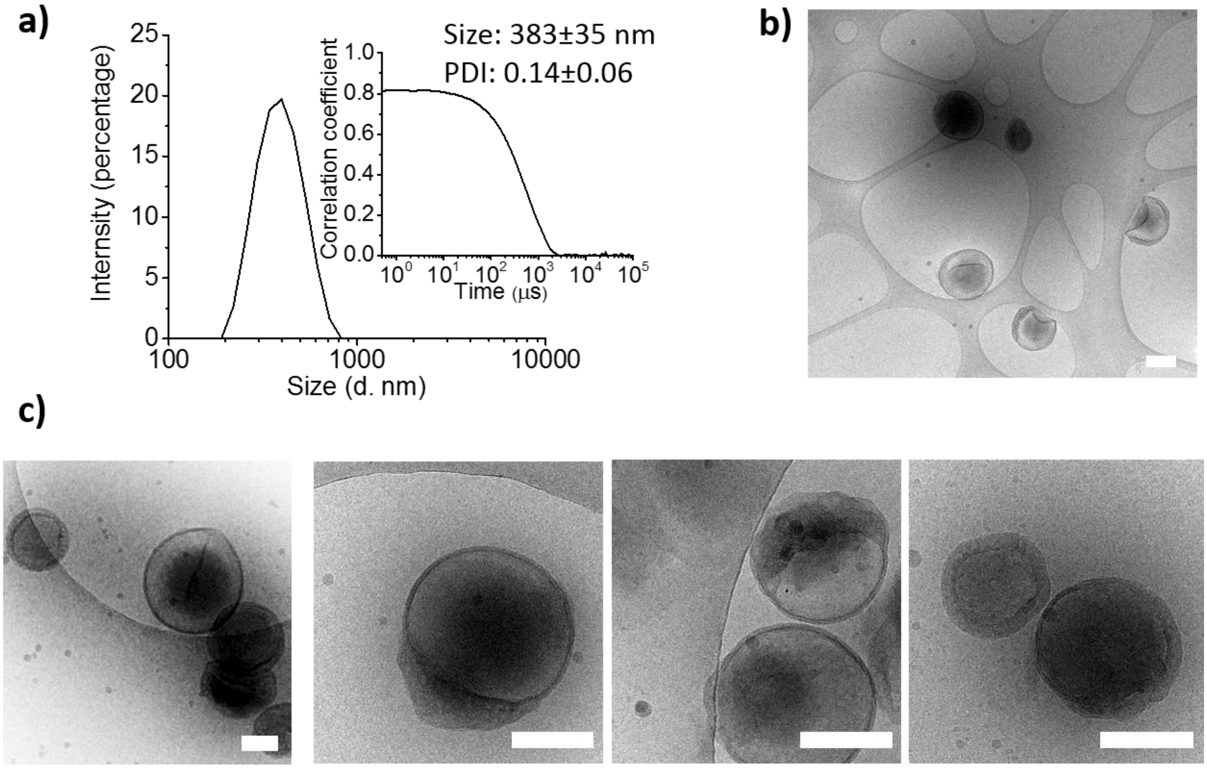

Supplementary Figure 35. Characterization of AIE/Au nanomotors fabricated from PEG_{44}-$PAIE_{14}$ polymersomes. (a) size measurement by DLS; (b) dry TEM imaging of AIE/Au nanomotors; (c) low-resolution of cryo-TEM images of AIE/Au nanomotor; (d) high-resolution cryo-TEM imaging techniques with higher defocus setup; (e) the corresponding inverse-contrast image by subtracting the background in (d). From (e), the membrane structure of AIE/Au nanomotor are clearly observed. Scale bar=100 nm.

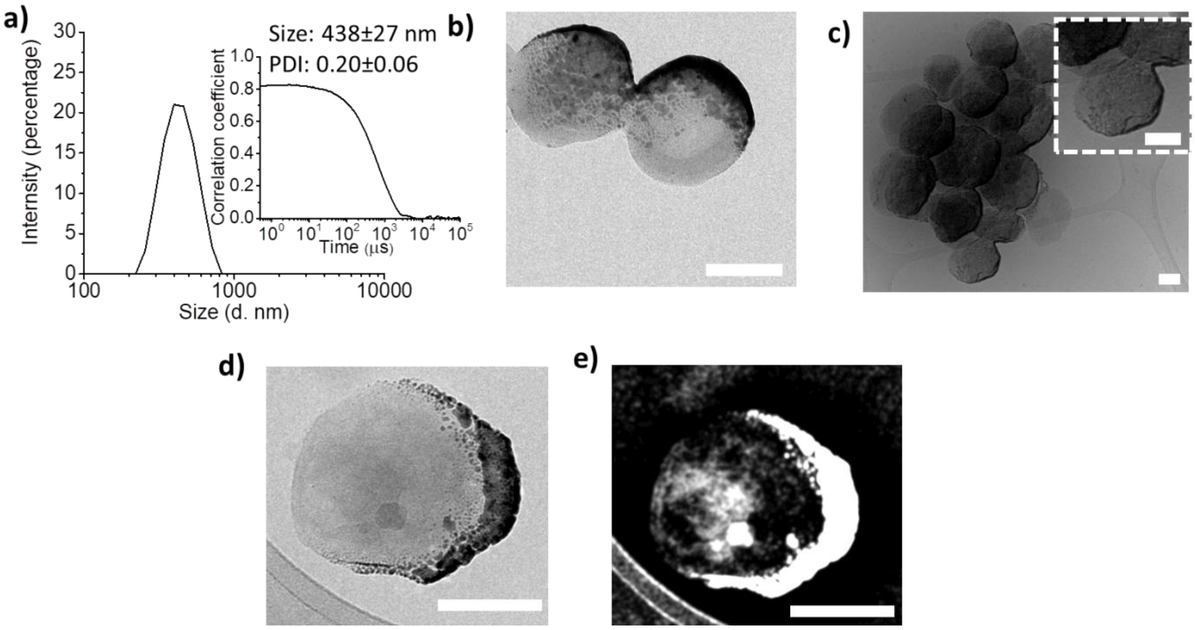

Supplementary Figure 36. Visualizing the 3D structure of an AIE/Au nanomotor using cryo-ET. (a) Represented gallery of *z*-slices showing different cross sections through a 3D reconstruction of an AIE/Au nanomotor shown in Supplementary Movie S2. In these slices, the polymersome membrane, the coated layer distribution as well as the interior structure of the AIE/Au motor are visible. The dashed orange squares highlight the fiducial gold nanoparticles. (b) Schematic showing where the cross sections shown in (c) from different orthogonal planes (XY, XZ and XZ) were taken. (c) Overview of cross-sections from three different orthogonal planes showing the 3D interior structure. The white arrow in XZ plane points out the presence of the polymersome membrane.

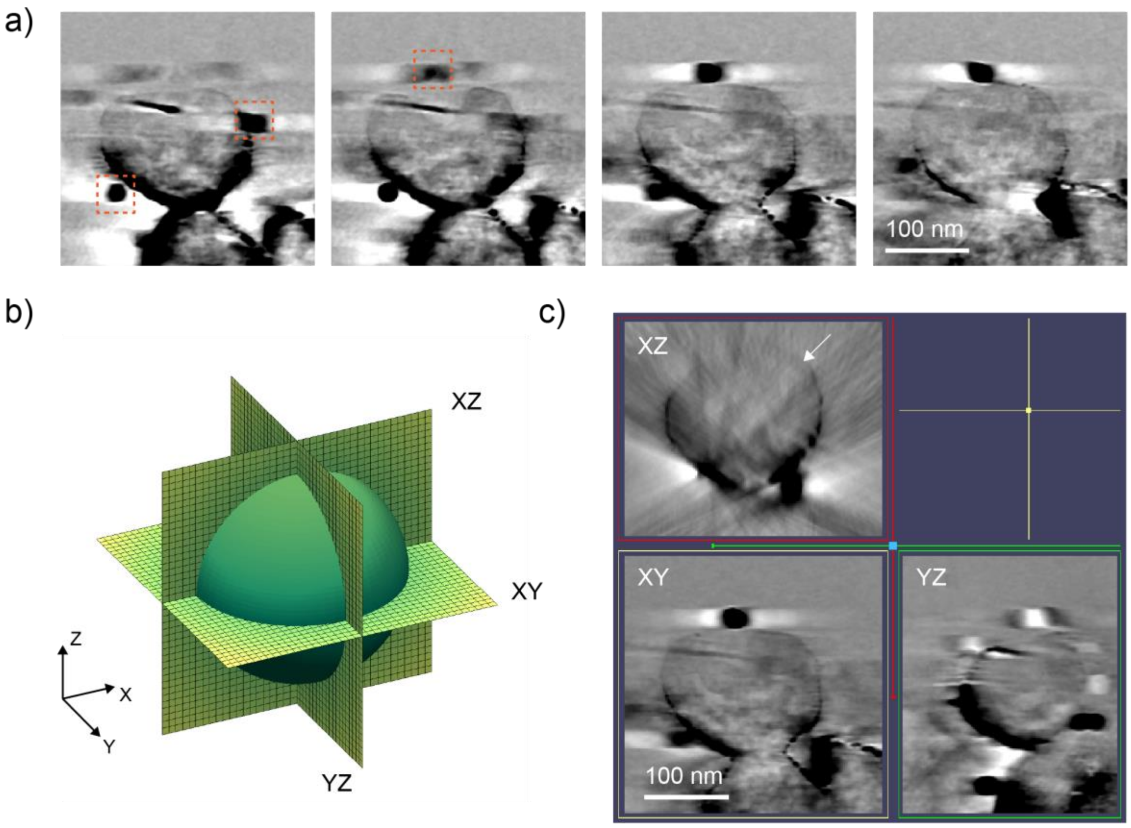

Supplementary Figure 37. Cryo-TEM images of an AIE/Au nanomotor at different tilting angle, where 20 nm gold fiducial nanoparticles are present. We note that due to the significant contrast difference between the coated Au layer and the polymersome membrane, the membrane structure can be only seen when a large defocus value is applied. Scale bar=100 nm.

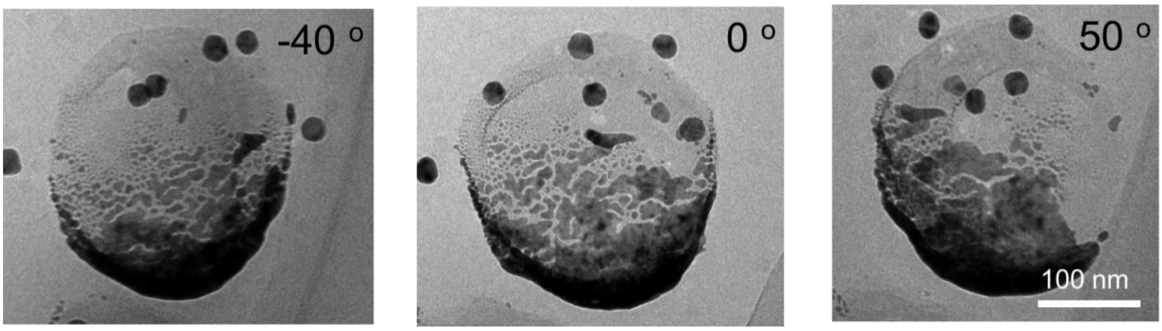

Supplementary Figure 38. Hybrid AIE/Au nanomotors retain the ability to encapsulate hydrophobic and hydrophilic cargo in the form of fluorescent Cy7 and dextran-TMR, respectively. (a) Fluorescent emission intensity curve of blank AIE/Au nanomotor and dextran-TMR loaded AIE/Au nanomotor (after centrifugation and re-dispersion into aqueous medium), λ_{ex}=535 nm. (b) Relative release of dextran-TMR after re-dispersing into aqueous medium. (c) Fluorescent emission intensity curve of blank AIE/Au nanomotor and Cy7 loaded AIE/Au nanomotor (after centrifugation and re-dispersion into aqueous medium), λ_{ex}=720 nm. (d) Emission intensity of Cy7 loaded AIE/Au nanomotor after re-dispersing into aqueous medium, whereby retention of the cargo is indicated by a very slow decrease in the fluorescence as Cy7 is released into the surrounding solution (where it is non-fluorescent). Error bars = Standard Deviation (n=5).

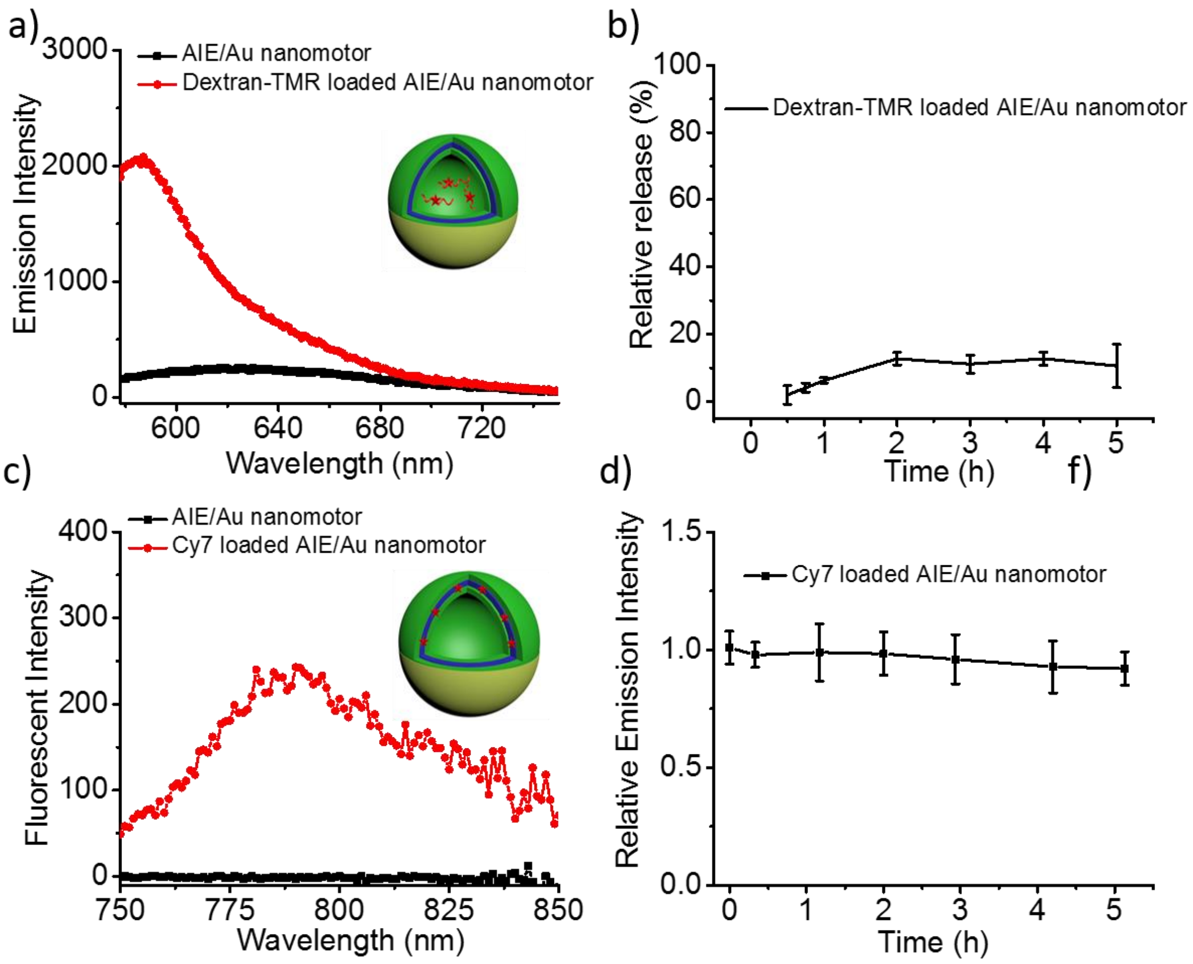

Supplementary Figure 39. (a) UV-Vis spectra of AIE/Au nanomotors. (b) Photographs of AIE-polymersomes (AIE-Ps) and AIE/Au nanomotors (AIE/Au NM) illuminated with visible light (left) and with a lab UV-lamp (right). (c) Representative confocal images of AIE-polymersomes and AIE/Au nanomotors (λ_{ex}=405 nm, λ_{em}=650~700 nm).

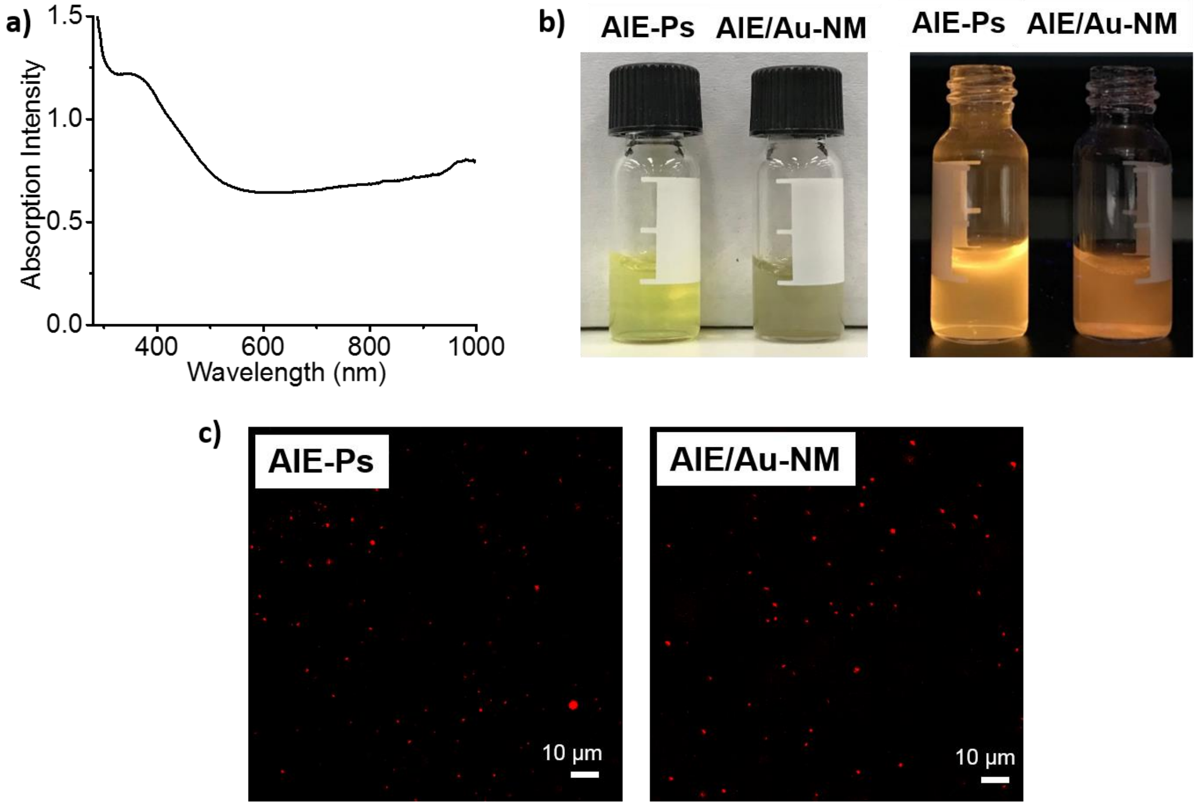

Supplementary Figure 40. Time lapse images of AIE/Au nanomotors (particle concentration: 12.5 μg ml^{-1}) in the absence (top) and presence (bottom) of TP-NIR laser irradiation (laser out power: 0.1 J cm^{-2}) scale bar = 20 μm. The dashed lines indicate the position of particles in the current frame and previous frames. (Fluorescent images were taken with λ_{ex}=405 nm and λ_{em}=650~700 nm; TP-NIR laser wavelength was set at 760 nm).

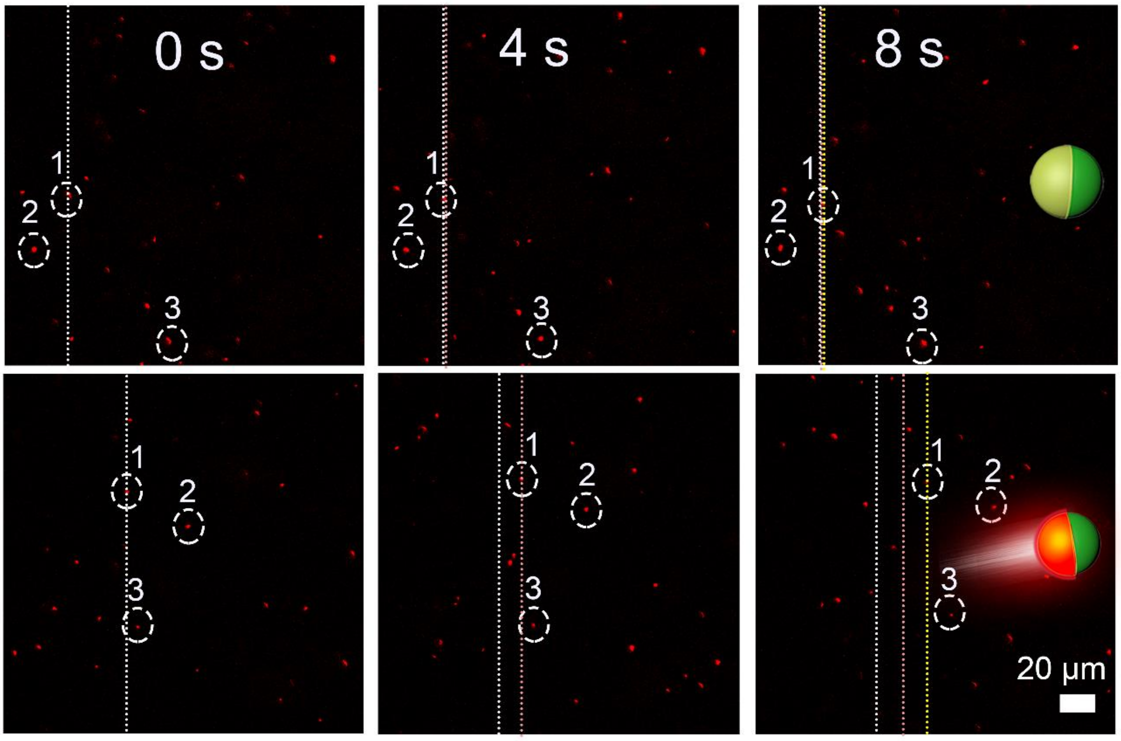

Supplementary Figure 41. Trajectories of AIE/Au nanomotors (particle concentration: 12.5 μg ml^{-1}) in the presence of incident TP-NIR laser power at different laser intensities. (Fluorescent images were taken with λ_{ex}=405 nm and λ_{em}=650~700 nm; TP-NIR laser wavelength was set at 760 nm).

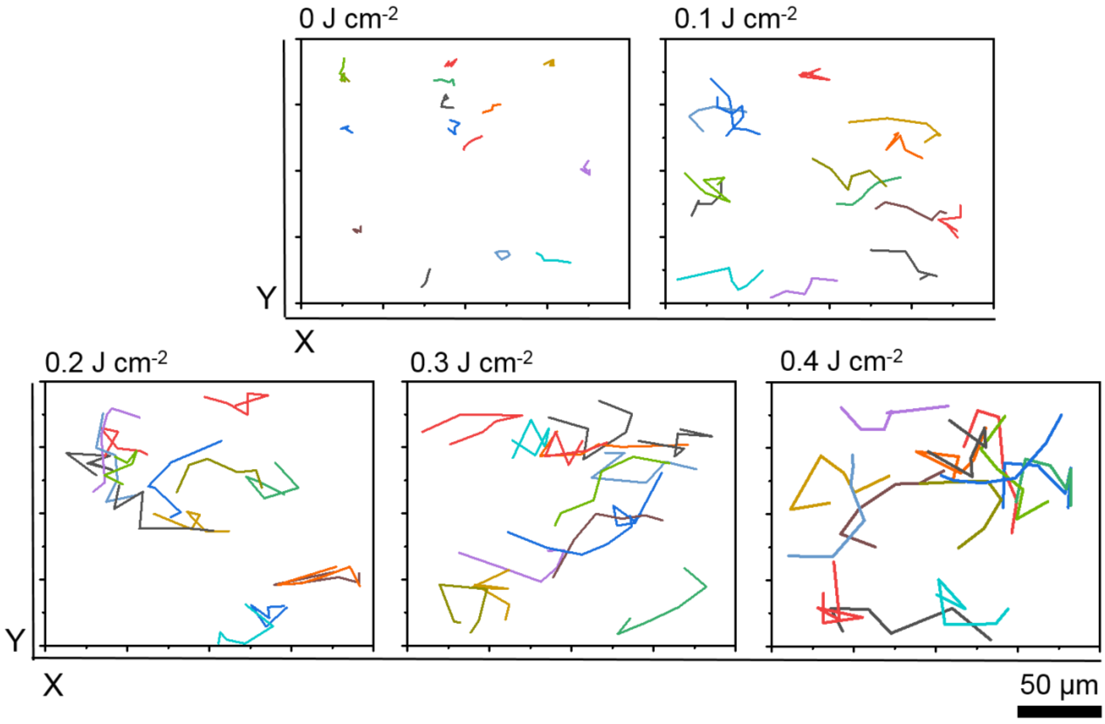

Supplementary Figure 42. (a) Non-AIE polymersome formulation comprising PEG_{44}-$PDLLA_{115}$ block copolymers, followed by sputter gold coating to give the non-AIE nanomotors as control, via an identical method to AIE/Au nanomotors. (b) DLS intensity plots of PEG-PDLLA polymersomes (non-AIE polymersomes) and non-AIE nanomotors. (c) Trajectories of the control non-AIE nanomotors in the presence of incident TP-NIR laser power at different intensities (particle concentration: 12.5 μg ml^{-1}).

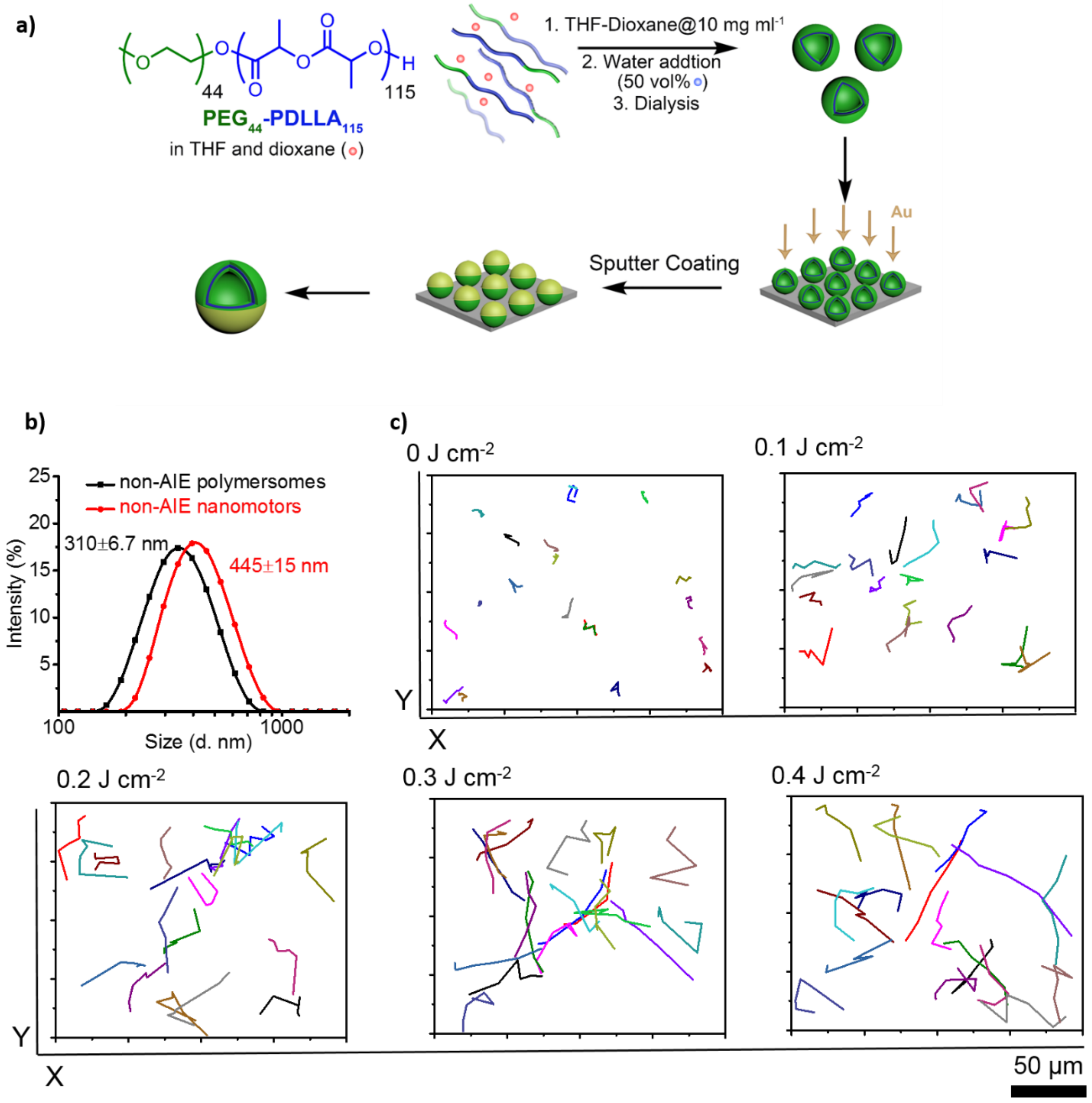

Supplementary Figure 43. Comparison between velocities of non-AIE nanomotors (fabricated from PEG-PDLLA polymersomes) and AIE/Au nanomotors (fabricated from AIE-polymersomes) exposed to different TP-NIR laser intensities. Error bars = Standard Deviation (n=5).

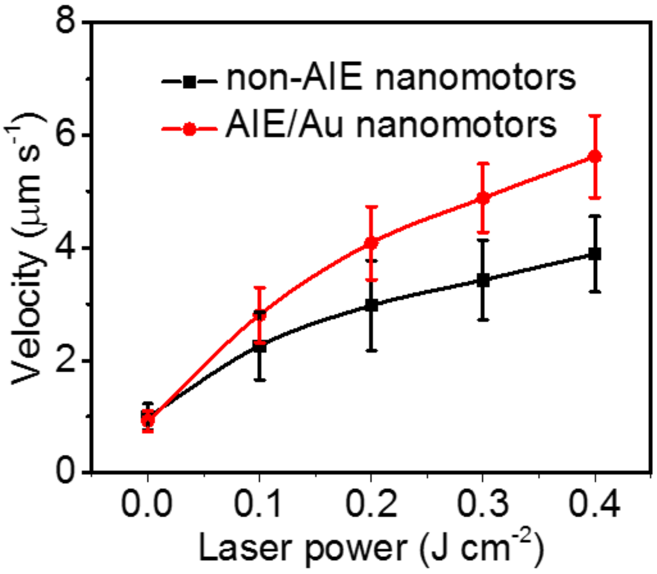

Supplementary Figure 44. Normalized trajectory of AIE/Au nanomotors under (a) 405 nm light irradiation and (b) 488 nm light irradiation (Laser output: 50 mW cm^{-2}). Average MSD curves and standard deviation of AIE/Au nanomotors under (c) 405 nm light irradiation and (d) 488 light irradiation. (e) Comparison of diffusion coefficients of AIE/Au nanomotor under 405 nm and 488 nm light irradiation. (f) Hydrodynamic size (as determined from nanoparticle tracking analysis (NTA)) of AIE/Au nanomotors under 405 nm and 488 nm light irradiation. Error bars = Standard Deviation (n=3).

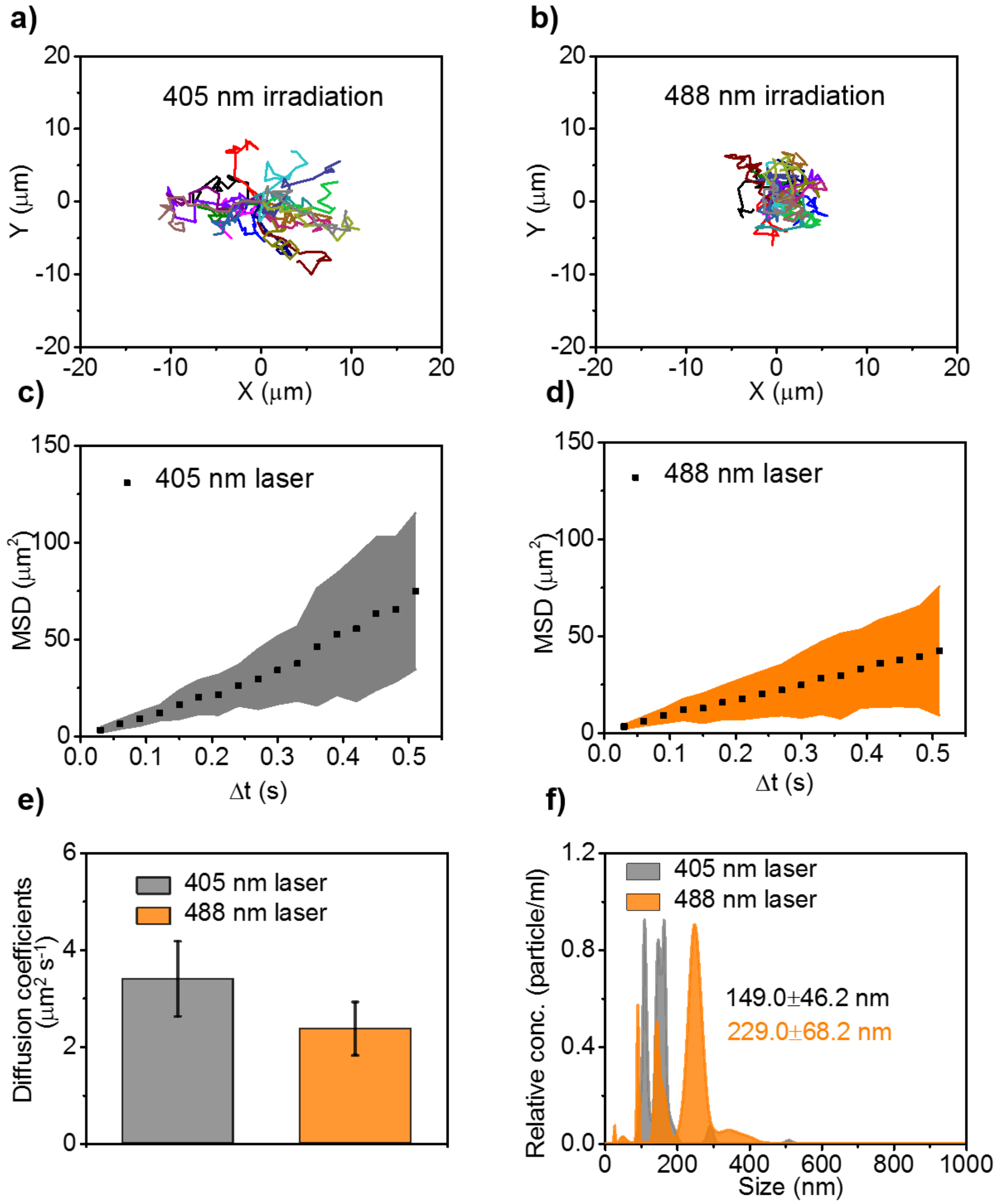

Supplementary Figure 45. Normalized trajectory of AIE-polymersomes under (a) 405 nm light irradiation and (b) 488 nm light irradiation (Laser output: 50 mW cm^{-2}). (c) MSD fitting curve of AIE-polymersomes under 405 nm and 408 nm light irradiation. Average MSD curves and standard deviation of AIE-polymersomes under (d) 405 nm light irradiation and (e) 488 light irradiation. Comparison of (f) diffusion coefficients and (g) size from nanoparticle tracking analysis (NTA) of AIE-Ps under 405 nm and 488 nm light irradiation. Error bars = Standard Deviation (n=3).

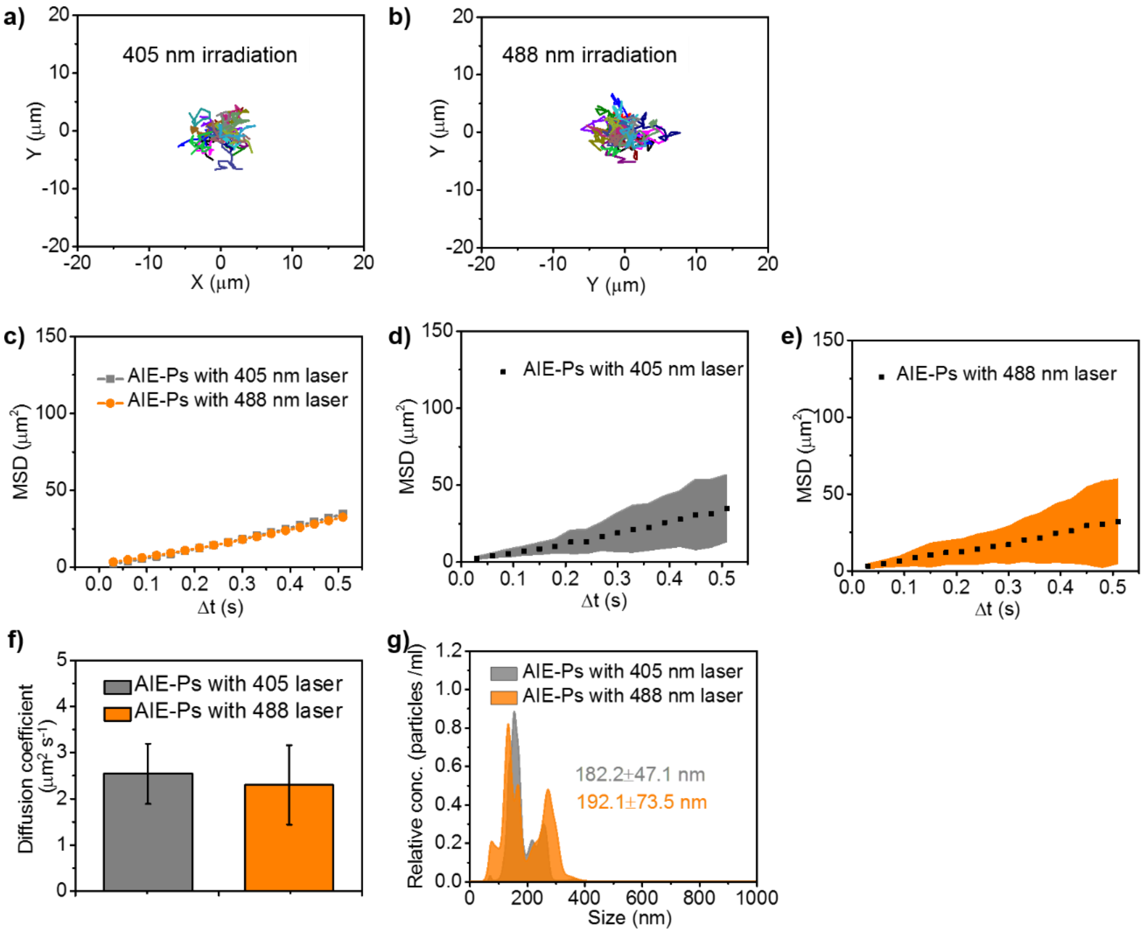

Supplementary Figure 46. Normalized trajectory of non-AIE nanomotors (from PEG-PDLLA polymersomes) under (a) 405 nm light irradiation and (b) 488 nm light irradiation (Laser output: 50 mw cm^{-2}). (c) MSD fitting curve of non-AIE nanomotors under 405 nm and 408 nm light irradiation. Average MSD curves and standard deviation of non-AIE nanomotors under (d) 405 nm light irradiation and (e) 488 nm light irradiation. Comparison of (f) diffusion coefficients and (g) size from nanotracking analysis (NTA) of non-AIE nanomotors under 405 nm and 488 nm light irradiation. Error bars = Standard Deviation (n=3).

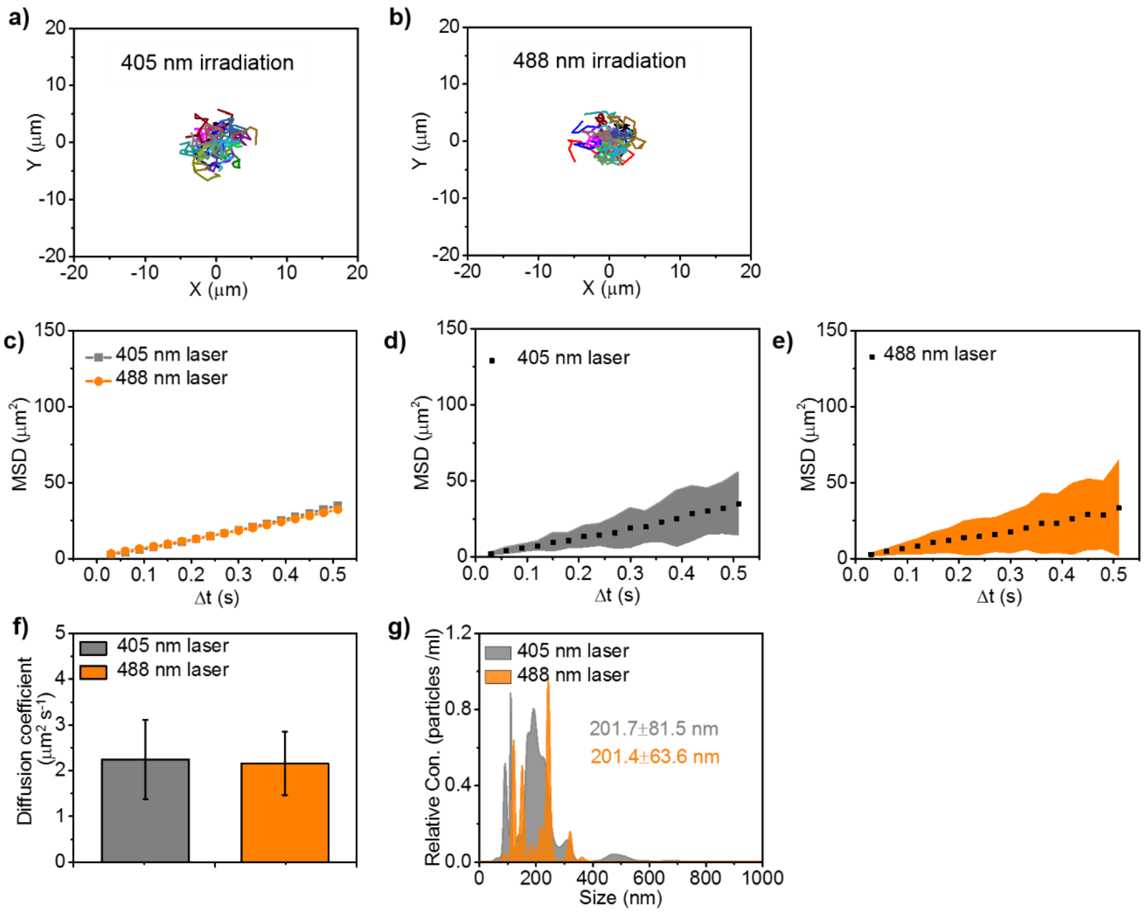

Supplementary Figure 47. Negative phototaxis behavior and normalized trajectory of AIE/Au nanomotors under near-infrared 660 nm (out-put power: 1 W) light irradiation. The motion direction of AIE/Au nanomotors is always opposite to the direction of the incident laser light (because of the negative phototaxis behavior).

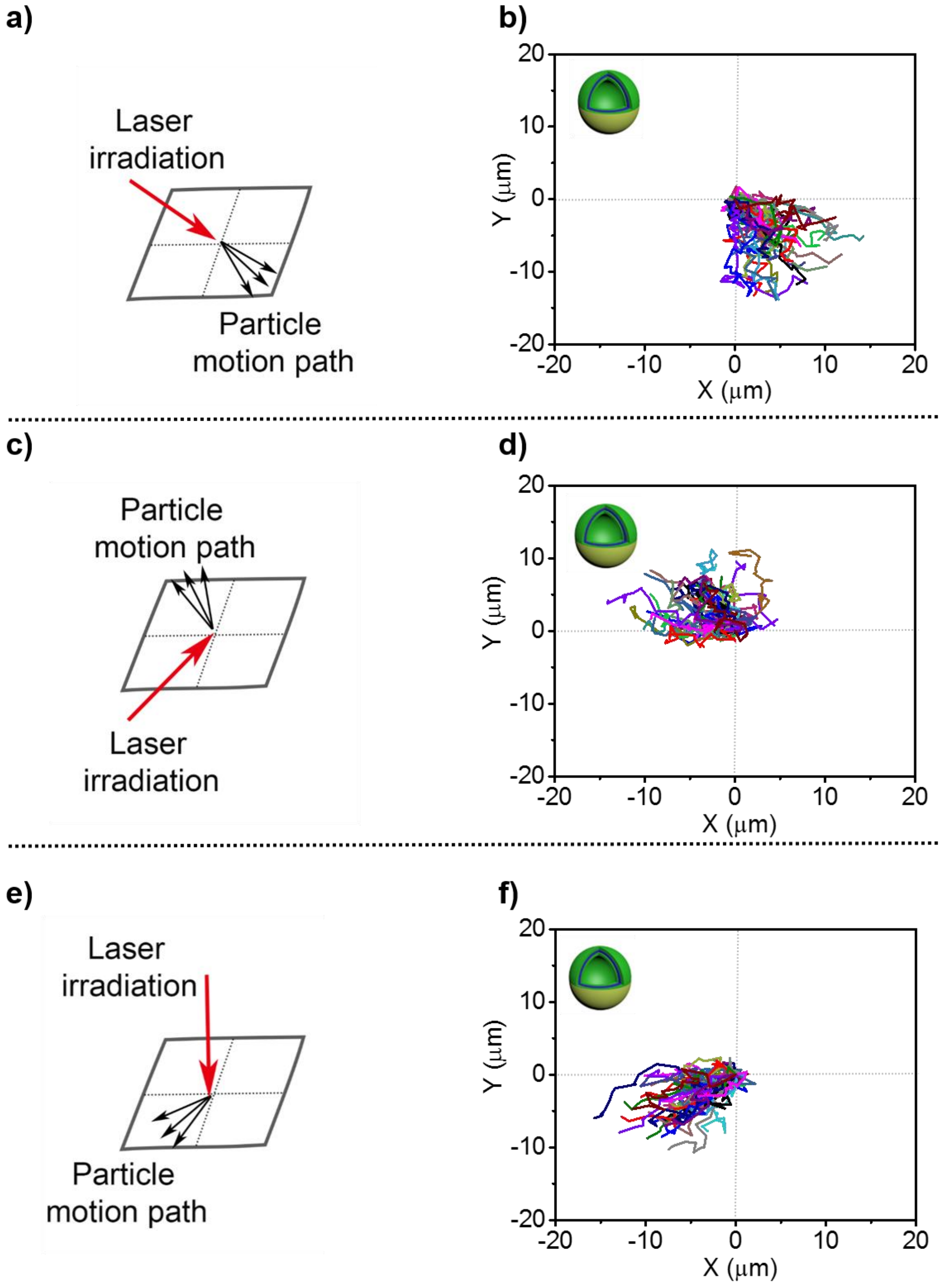

Supplementary Figure 48. Motion behavior of AIE/Au nanomotors in pure cell culture medium. (a) MSD fitting curve of AIE/Au nanomotors with or without NIR 660 light irradiation (out-put: 1W) in pure cell culture medium. (b) Trajectory of AIE/Au nanomotors without light irradiation; (c) trajectory of AIE/Au nanomotors under near-infrared 660 nm (out-put power: 1 W) light irradiation.

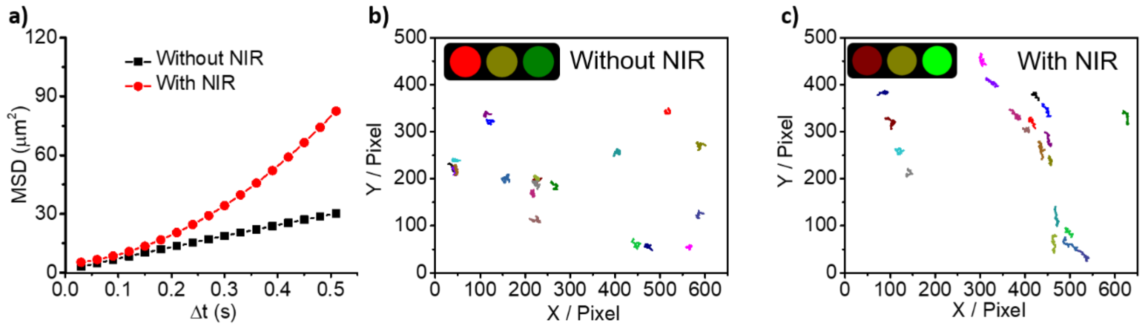

Supplementary Figure 49. Gold-nanoshells as symmetric (plasmonic) control particles. (a) Size of gold-nanoshells measured via Nanosight; (b) Set-up of light-irradiation in the Nanosight. (c) MSD analysis data of gold-nanoshells with or without NIR (660 nm infrared laser, output: 1 W) light irradiation. (d) Normalized trajectory of gold-nanoshells in the absence of NIR irradiation. (e) Normalized trajectory of gold-nanoshells under NIR irradiation.

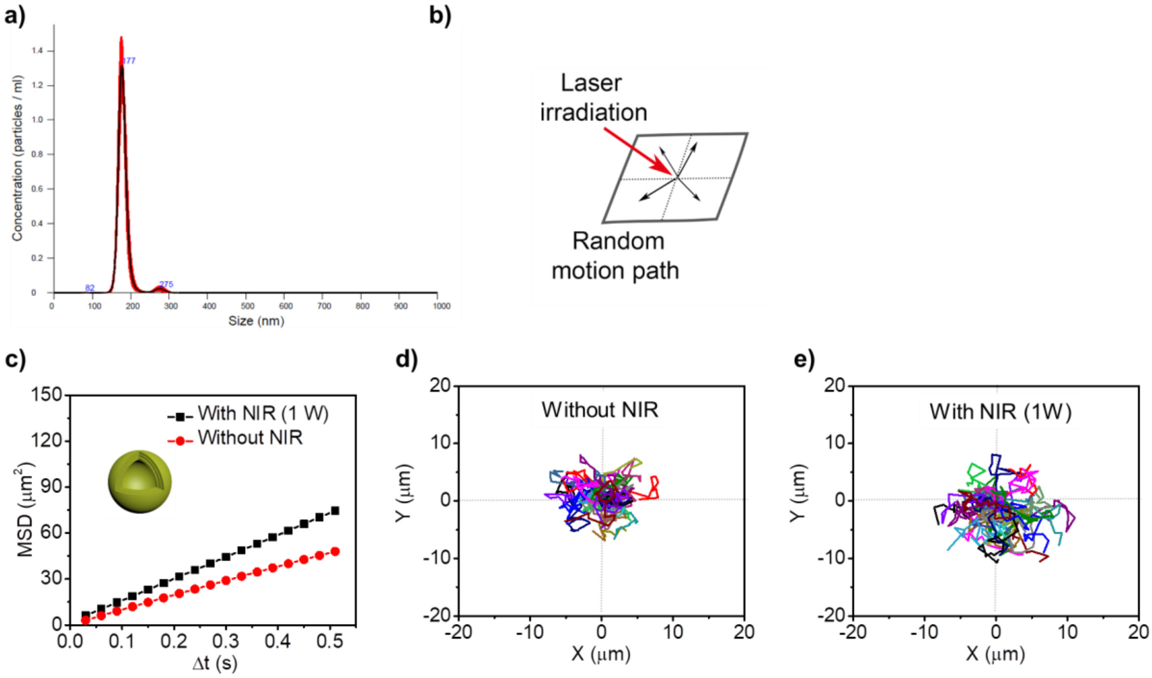

Supplementary Figure 50. AIE-polymersomes as symmetric (non-plasmonic) control particles. (a) Set-up of the light-irradiation in the Nanosight. (b) MSD analysis data of AIE-polymersomes in the absence and presence of NIR (660 nm infrared laser, output: 1W) light irradiation. (c) Normalized trajectory of AIE- polymersomes in the absence of NIR irradiation. (d) Normalized trajectory of AIE- polymersomes under NIR irradiation.

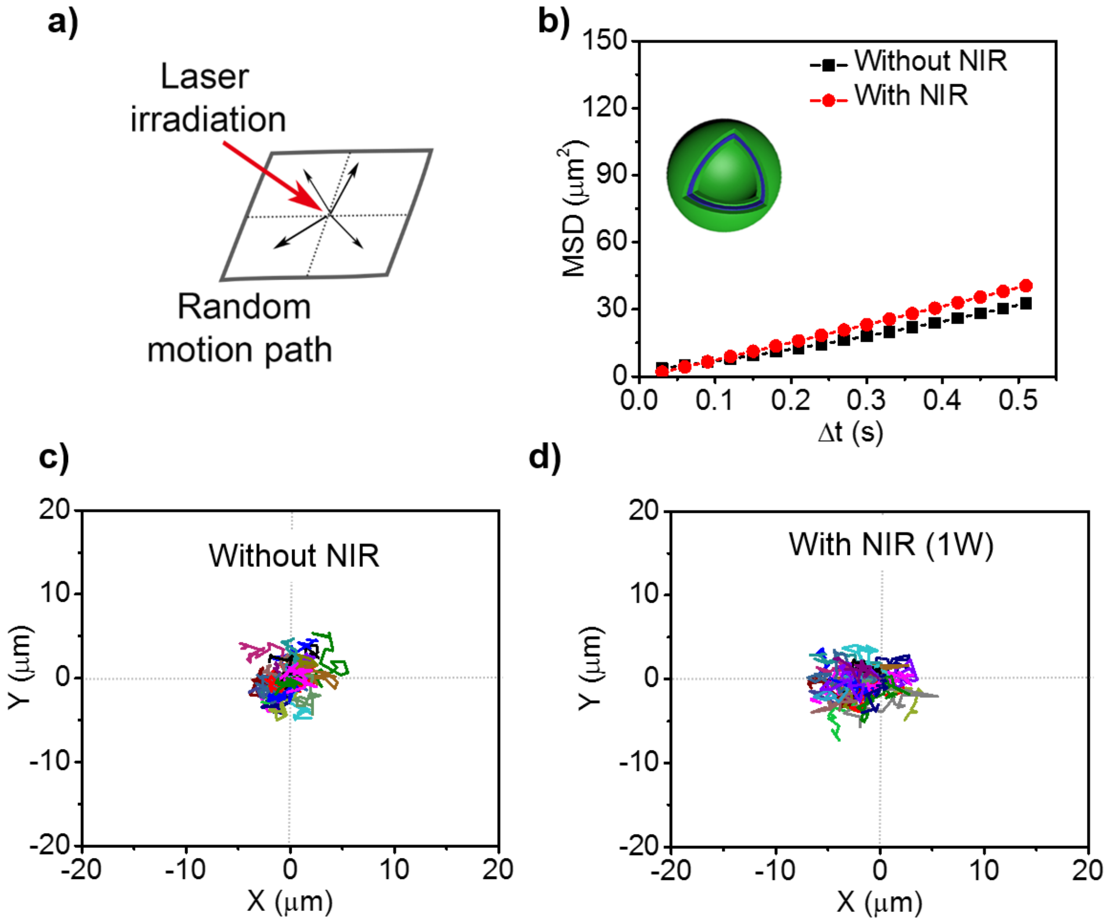

Supplementary Figure 51. Internalization of PEG_{44}-$P(AIE)_{14}$ AIE-polymersomes in HeLa cells upon incubation for different periods (2 h, 6 h and 24 h).

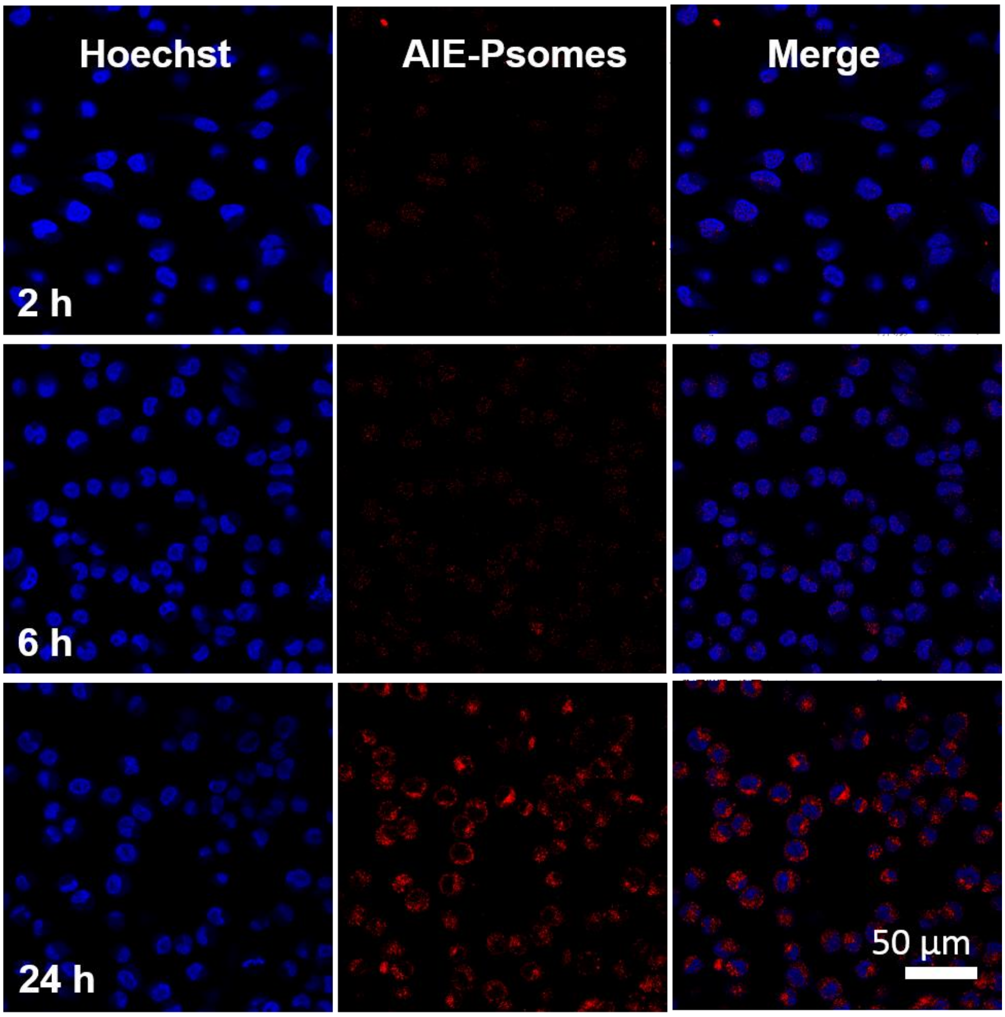

Supplementary Figure 52. Viability of HeLa cells treated with PEG_{44}-$P(AIE)_{14}$ AIE-polymersomes or AIE/Au nanomotors for 24 h, as determined by MTT assay. Error bars = Standard Deviation (n=5).

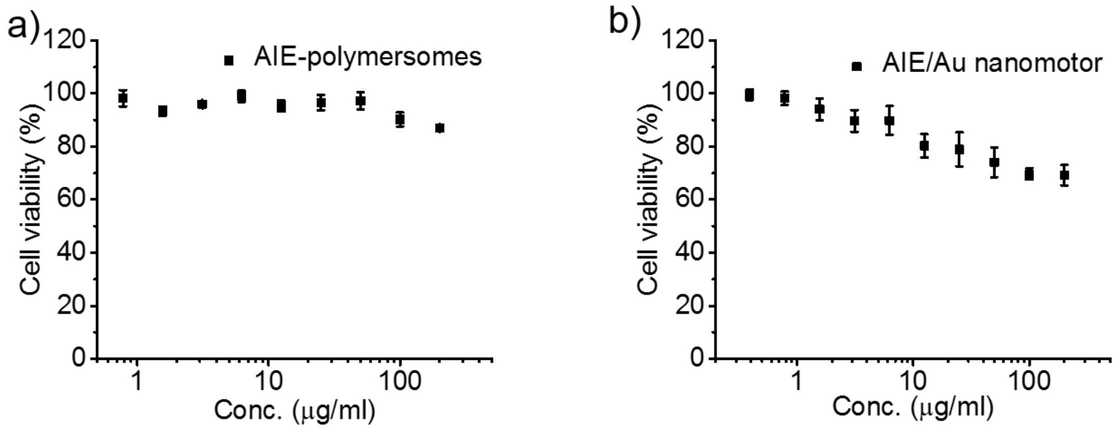

Supplementary Figure 53. Viability of HeLa cells treated with PBS blank as a function of confocal TP-NIR laser irradiation time (Output power: 0.4 J cm^{-2}, TP-NIR wavelength was set at 760 nm). After 200 s continuous confocal TP-NIR laser irradiation (each scan represents 4 s TP-NIR laser irradiation, in total 200 s NIR laser irradiation time), there is negligible fluorescence change of PI (Propidium iodide), indicating that NIR laser irradiation did not impact the viability of HeLa cells. Blue color indicates the nucleus staining dye Hoechst; Green color indicates calcein staining (live cells); red color (not visible) represents PI staining (dead cells).

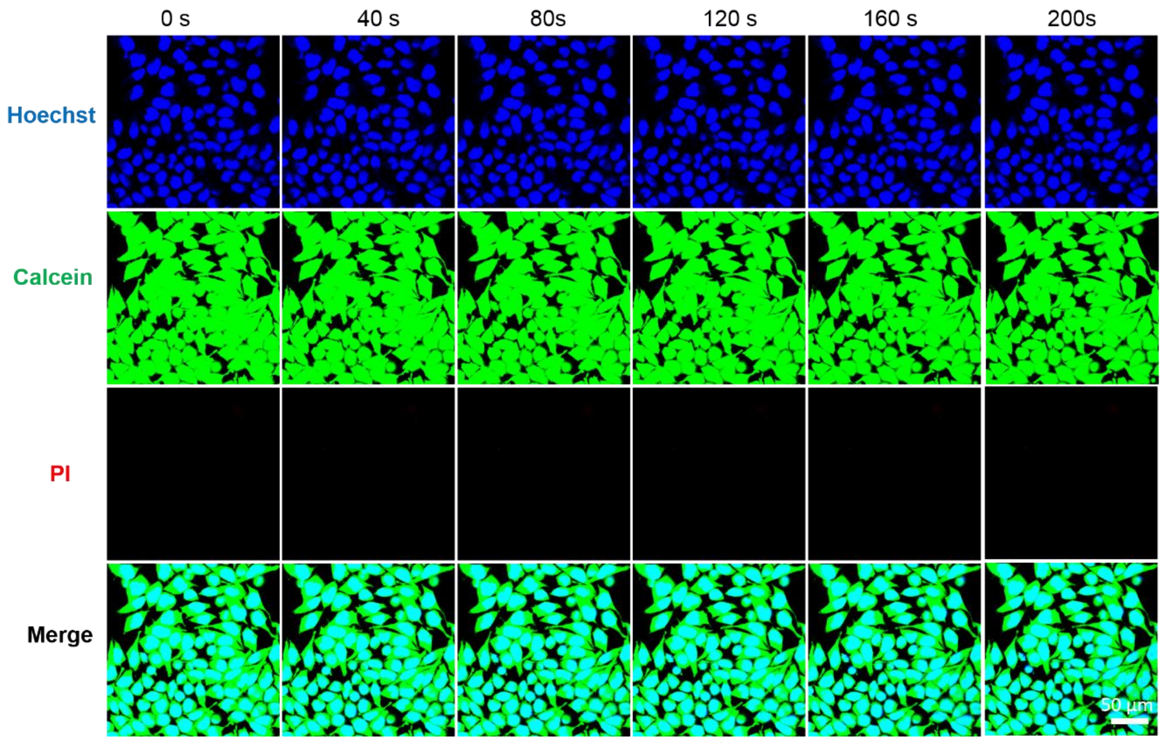

Supplementary Figure 54. Viability of HeLa cells treated with PEG_{44}-$P(AIE)_{14}$ AIE-polymersomes (25 μg ml^{-1}) as a function of confocal NIR laser irradiation time (Output power: 0.4 J cm^{-2}, TP-NIR wavelength was set at 760 nm). After 200 s confocal NIR laser irradiation (each scan represents 4 s TP-NIR laser irradiation, in total 200 s NIR laser irradiation time), there is negligible fluorescence change of PI which indicates AIE-polymersomes did not affect the viability of HeLa cells with TP-NIR irradiation. Blue indicates the nucleus staining dye Hoechst, green indicates calcein staining (live cells), red stain represents PI staining (dead cells).

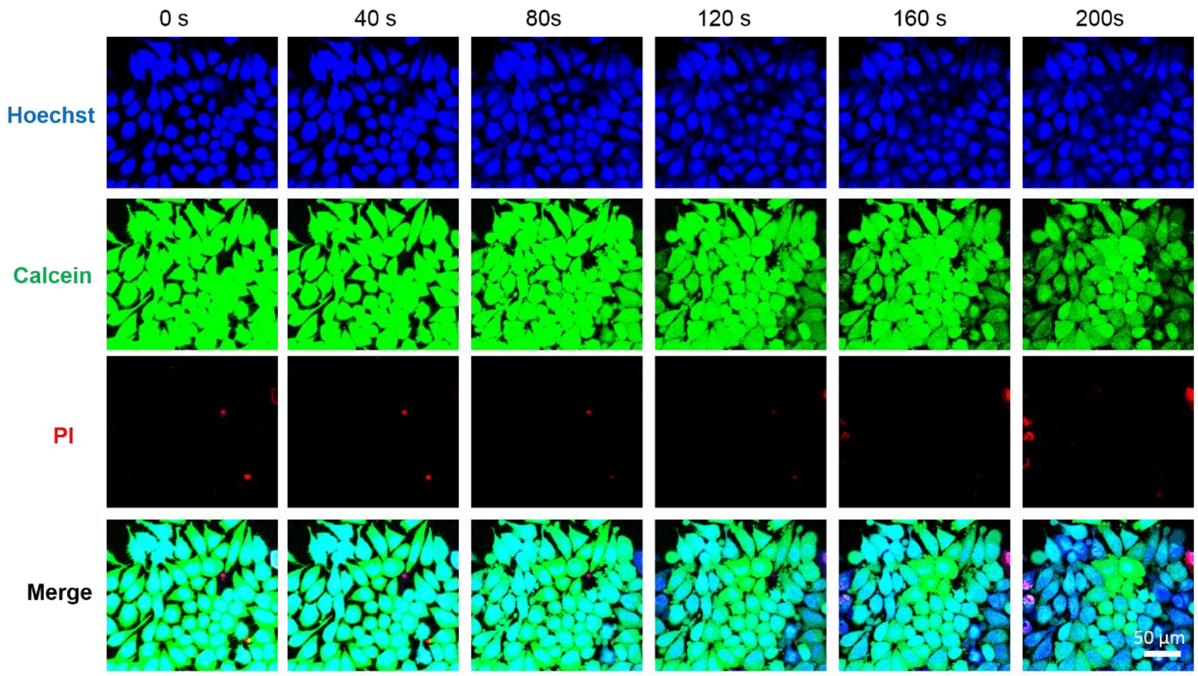

Supplementary Figure 55. Viability of HeLa cells treated with AIE/Au nanomotors (25 μg ml^{-1}) without confocal TP-NIR laser irradiation (Output power: 0 J cm^{-2}, TP-NIR wavelength was set at 760 nm). After 200 s confocal TP-NIR laser irradiation (each scan represents 4 s TP-NIR laser irradiation, in total 200 s TP-NIR laser irradiation time), there is no big fluorescence change of calcein or PI which indicates AIE/Au nanomotors did not impact the viability of HeLa cells without TP-NIR irradiation. Blue indicates the nucleus staining dye Hoechst, green indicates calcein staining (live cells), red stain represents PI staining (dead cells).

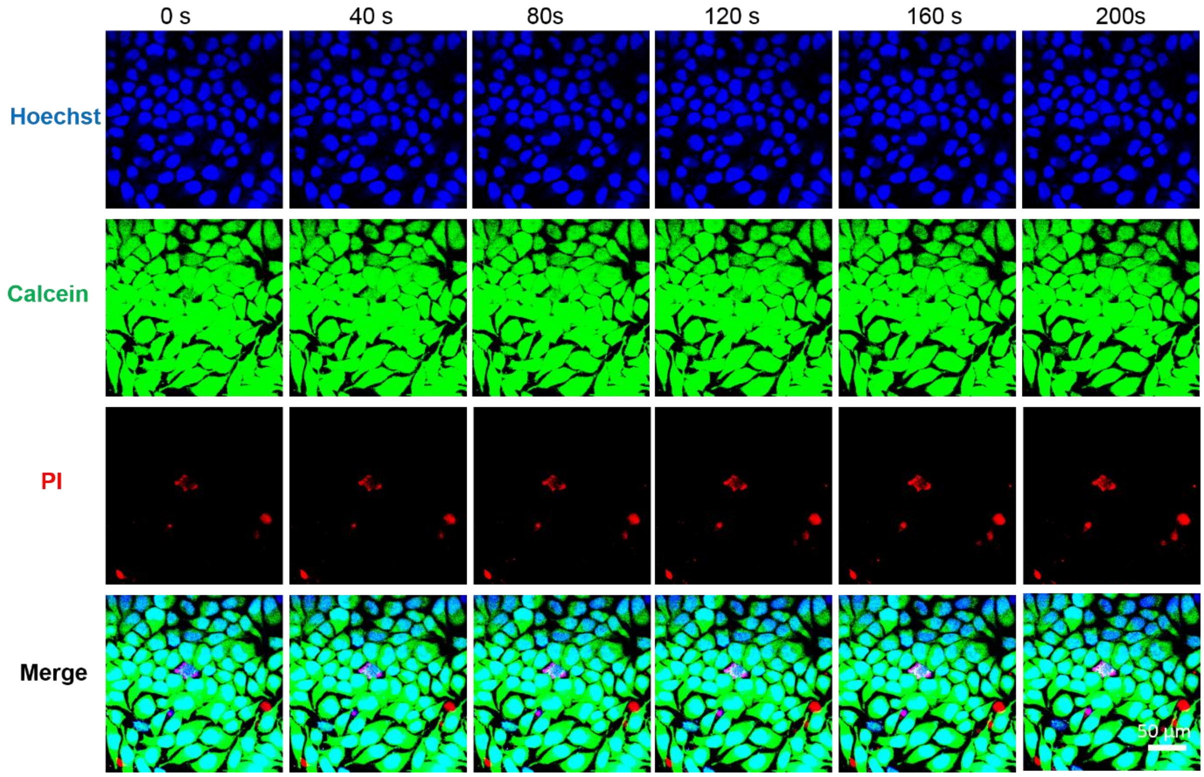

Supplementary Figure 56. Membrane destabilization of HeLa cells treated with AIE/Au nanomotors as a function of confocal TP-NIR laser illumination time (Output power: 0.4 J cm^{-2}, TP-NIR wavelength was set at 760 nm). Green emission represents cells membrane, which were stained by wheat germ agglutinin (WGA) Alexa fluor-TM 488 conjugate, red emission originated from AIE/Au nanomotors (with λ_{ex}=405 nm and λ_{em}=650~700 nm). The white arrow indicates the membrane destruction induced by NIR laser irradiation, which is a result of NIR light induced photo-thermal effect and active movement of the AIE/Au nanomotors.

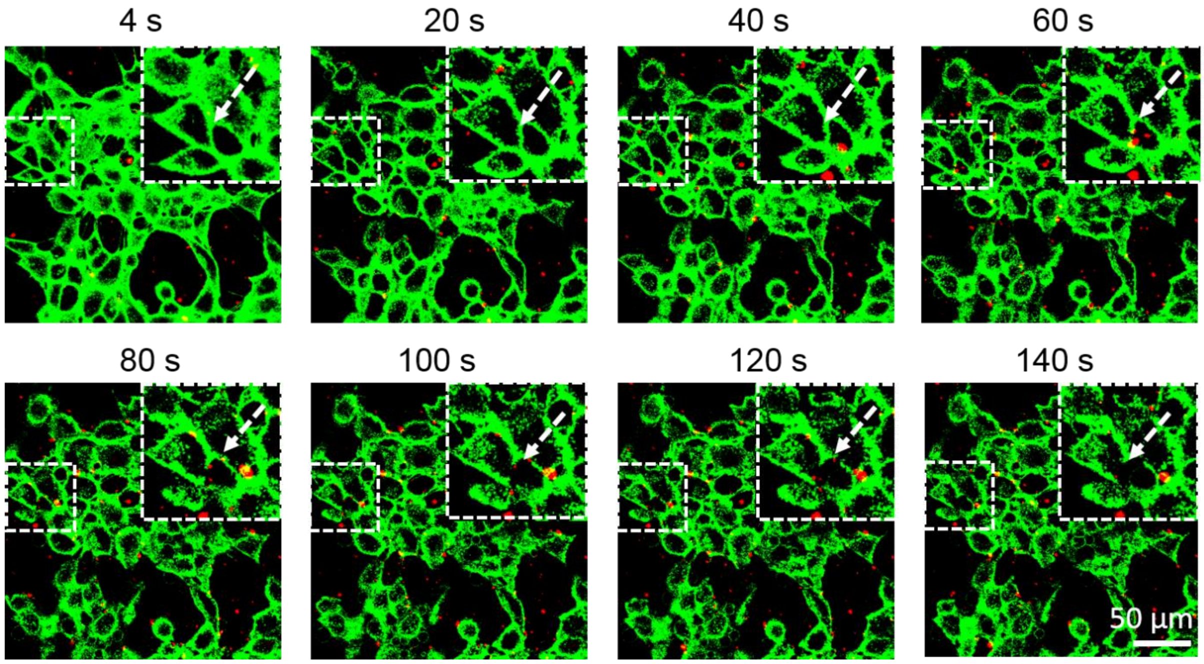

Supplementary Figure 57. ROS production of PEG_{44}-$P(AIE)_{14}$ AIE-polymersomes (200 µg ml^{-1}) within HeLa cells. After 24 h of incubation, cells were washed and imaged using time-dependent confocal TP-NIR laser illumination (output power: 0.4 J cm^{-2}, TP-NIR wavelength was set at 760 nm). The confocal analysis proved that the AIE-polymersomes internalized by HeLa cells are able to produce ROS with TP-NIR laser irradiation. Green emission indicates ROS product from DCFH-DA, blue emission indicates nucleus stained by Hoechst 33342.

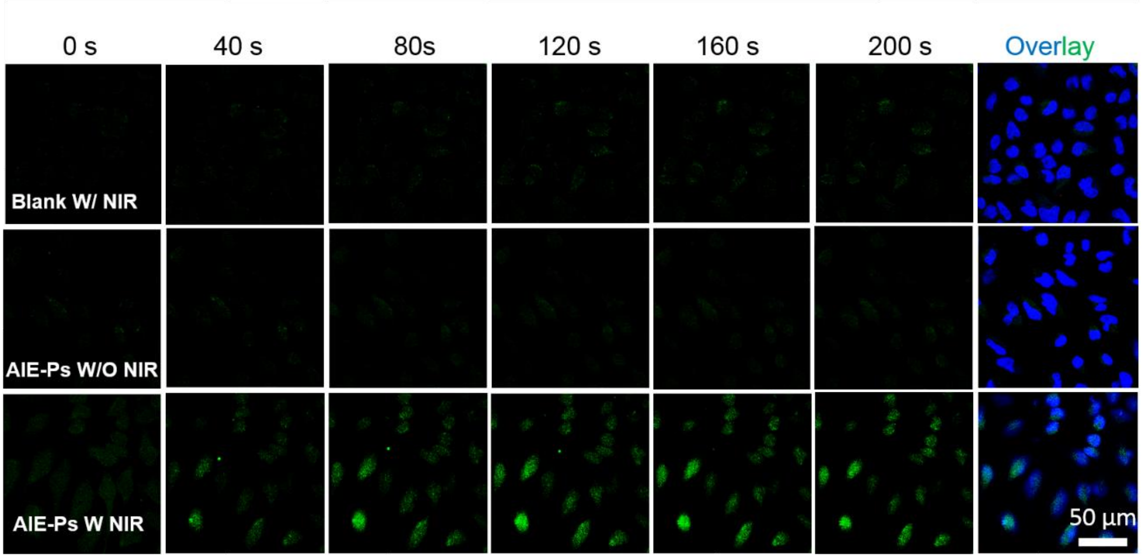

Supplementary Figure 58. Necrosis induced by continuous confocal TP-NIR laser irradiation (output power: 0.4 J cm^{-2}, TP-NIR wavelength was set at 760 nm) in HeLa cells after incubation with PEG_{44}-$P(AIE)_{14}$ AIE-polymersomes (200 µg ml^{-1}) for 24 h. Even after prolonged laser irradiation (280 s total TP-NIR irradiation time, 70 scans in total, per scan 4 s laser irradiation), there is only a minor part of the cells stained by PI (dead staining). In combination with the uptake results in Supplementary Figure 43, these results indicated that AIE-polymersomes internalized into cancer cells only resulted in inefficient photo-dynamic therapy and necrosis upon TP-NIR laser irradiation, which could be ascribed to the low uptake efficiency. Blue emission indicates nuclear stain, green emission represents cell membrane stain by wheat germ agglutinin (WGA)-Alexa fluor-TM 488 conjugate, red emission indicates propidium iodide (PI) staining,

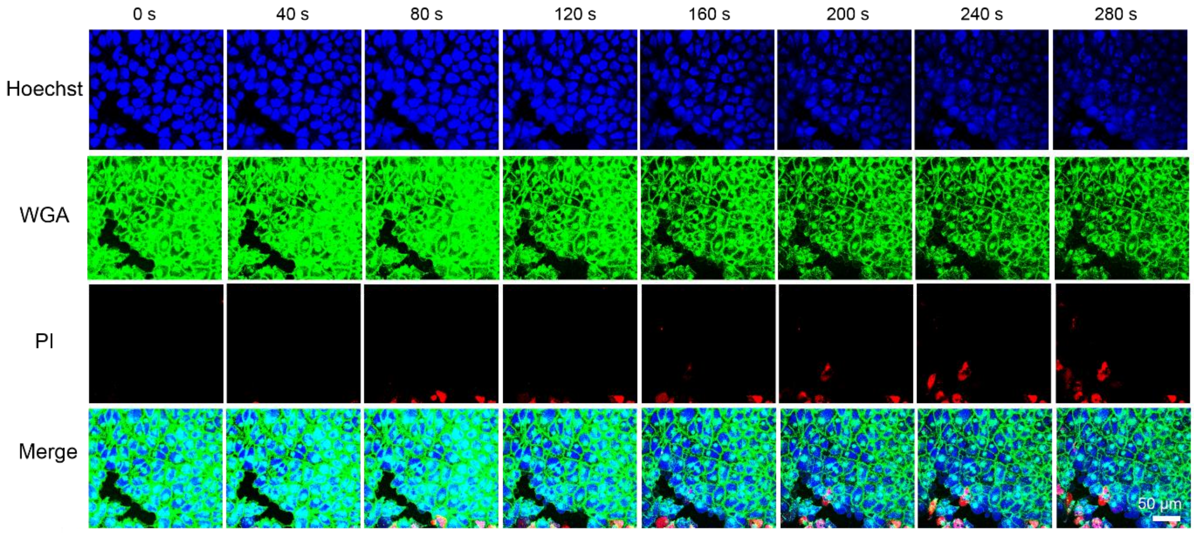

Supplementary Figure 59. ROS production in HeLa cells in a time-dependent manner under continuous confocal TP-NIR laser illumination (Output power: 0.4 J cm^{-2}, TP-NIR wavelength was set at 760 nm). HeLa cells were treated with PBS (blank), PEG_{44}-$P(AIE)_{14}$ AIE-polymersomes (25 μg ml^{-1}), AIE/Au nanomotors (25 μg ml^{-1}) in the absence or presence of confocal NIR laser illumination. Green color indicates DCFH-DA emission, blue color indicates nucleus stained with Hoechst 33342.

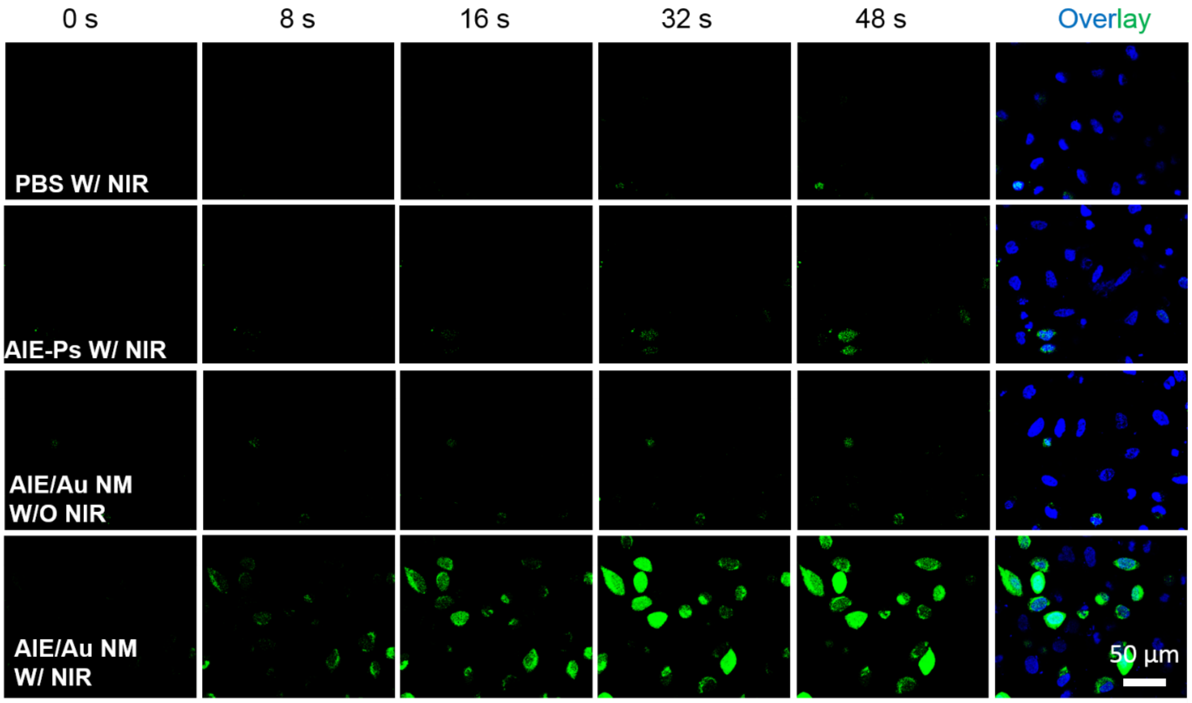

Supplementary Figure 60. (a) TP-NIR light irradiation (output power: 0.4 J cm^{-2}, TP-NIR wavelength was set at 760 nm) induced enhanced accumulation of PEG_{44}-$P(AIE)_{14}$ AIE/Au nanomotors on the surface of cancer cells and diffusion into cancer cells, resulting from TP-NIR-activated propulsion, in a time-dependent manner. The enhanced diffusion of AIE/Au nanomotors into cancer cells was likely caused by the membrane destabilization effect induced by the photo-thermal effect of the gold-shell AIE/Au nanomotors and active motion of AIE/Au nanomotors under TP-NIR light illumination. (b) Representative 3D reconstruction images of HeLa cells after 200 s of TP-NIR light illumination. The green fluorescence represents membrane staining by WGA Alexa Fluor 488 WGA dye, red emission indicates AIE/Au nanomotor location.

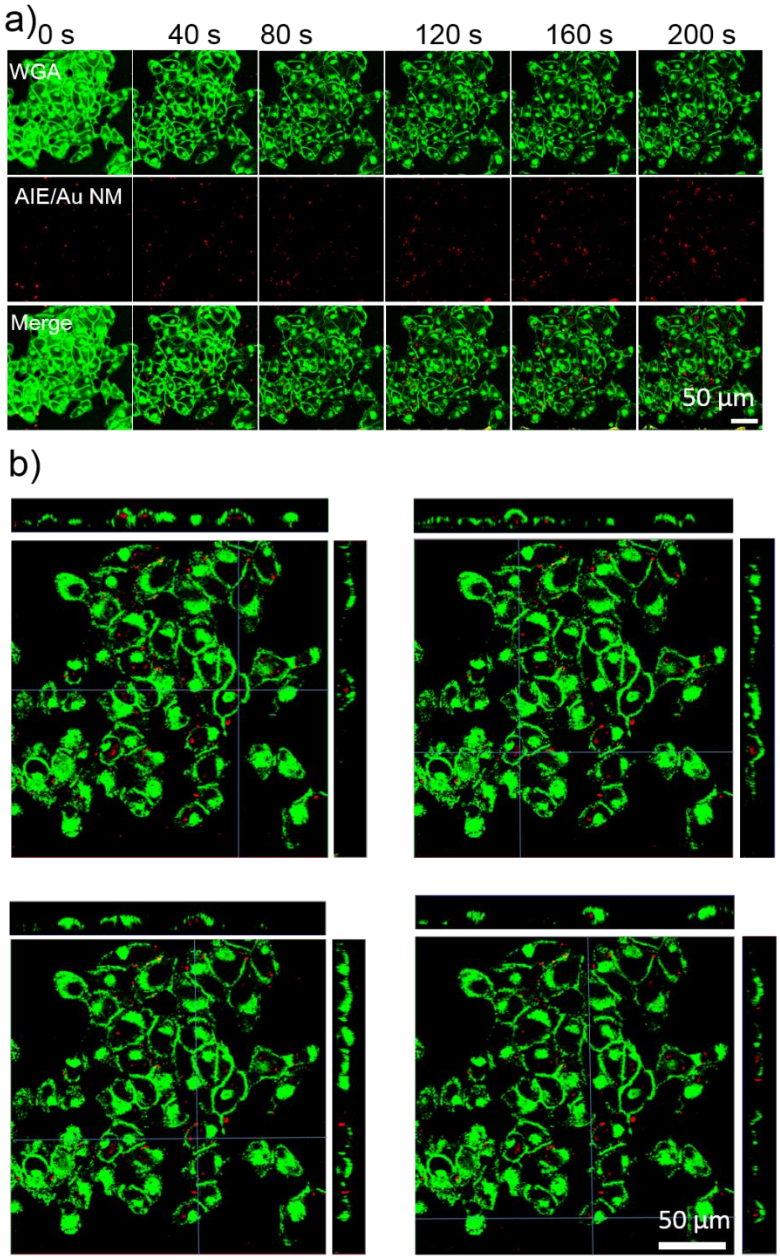

Supplementary Figure 61. Enhanced permeability of HeLa cell membranes after being treated with AIE/Au nanomotors (25 μg ml^{-1}) and TP-NIR irradiation. Images were obtained as a function of confocal TP-NIR laser irradiation time (output power: 0.4 J cm^{-2}, TP-NIR wavelength was set at 760 nm). Before TP-NIR treatment, most of the cells were non-permeable to propidium iodide (PI), indicating membrane integrity. Upon continuous TP-NIR laser irradiation, PI could stain the nucleus of HeLa cells in a time-dependent manner, indicating that AIE/Au nanomotors with TP-NIR irradiation destabilized the cell membrane and enhanced its permeability. Blue is the nucleus stained by Hoechst, red is the propidium iodide staining.

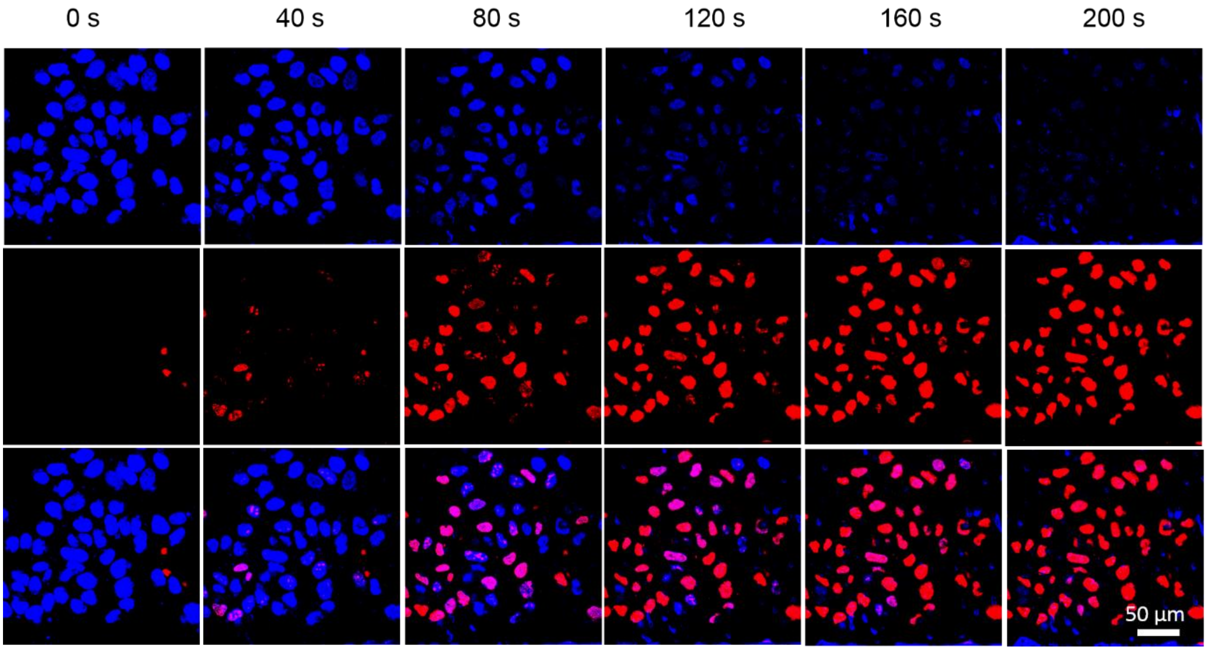

Supplementary Figure 62. Permeability of HeLa cell membranes after treatment with PBS, AIE-polymersomes, and AIE/Au nanomotors in the absence or presence of TP-NIR light irradiation. After 200 s confocal TP-NIR irradiation (50 scans in total, 4 s per TP-NIR irradiation, output power: 0.4 J cm^{-2}, TP-NIR wavelength was set at 760 nm), enhanced cell permeability was indicated by PI staining in the group of AIE/Au nanomotors with TP-NIR irradiation. Also, from the confocal analysis, there was negligible increase of PI signal in the control groups (PBS with TP-NIR irradiation, AIE-polymersomes with TP-NIR irradiation, and AIE/Au nanomotors without TP-NIR irradiation), indicating membrane integrity. This further highlights the key role of the combination of AIE/Au nanomotors with TP-NIR laser irradiation in cell membrane destruction. Blue indicates nucleus staining by Hoechst 33342, green indicates WGA Alexa Fluor 488 dye stained membrane, and red color indicates PI staining.

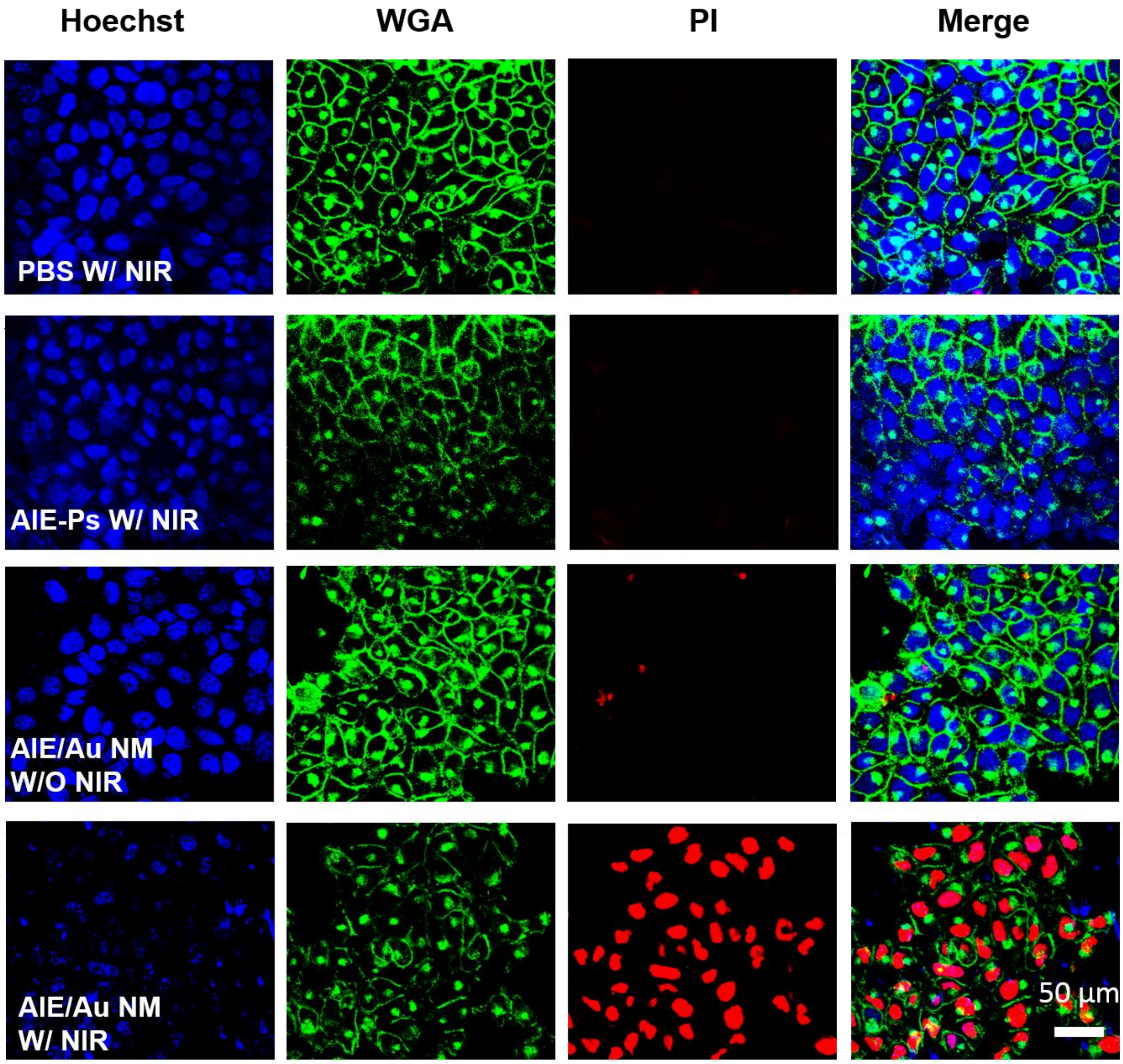

Supplementary Figure 63. Colocalization studies of AIE/Au nanomotors and lysosomes (stained with Lysol-tracker) in HeLa cells. HeLa cells are pre-stained with Lysol-tracker and treated with AIE/Au nanomotors, then irradiated with a continuous confocal NIR laser irradiation (Output power: 0.4 J cm^{-2}, TP-NIR wavelength was set at 760 nm). After 200 s of TP-NIR irradiation (50 scans in total, per TP-NIR irradiation is 4 s), there is little to no overlap of Lysol-tracker and AIE/Au nanomotors, indicating that the intracellular delivery of AIE/Au nanomotors under the executed NIR laser irradiation protocol is not dependent on the cellular endocytosis process.

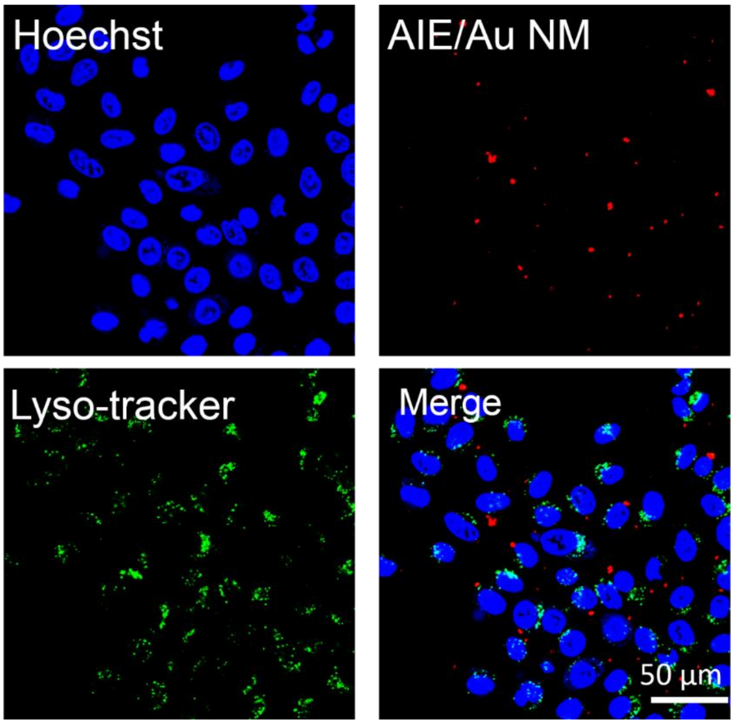

Supplementary Figure 64. Viability of HeLa cells treated with AIE/Au nanomotors as a function of confocal TP-NIR laser irradiation time (200 s TP-NIR irradiation in total, each TP-NIR irradiation scan is 4 s, output power: 0.4 J cm^{-2}, TP-NIR wavelength was set at 760 nm). PI gradually stained the nucleus of HeLa cells under continuous confocal NIR laser irradiation, which indicated AIE/Au nanomotors under TP-NIR laser irradiation caused fast necrosis. Blue color indicates the nucleus staining dye Hoechst; green indicates calcein staining (live cells); red color represents PI (dead cells).

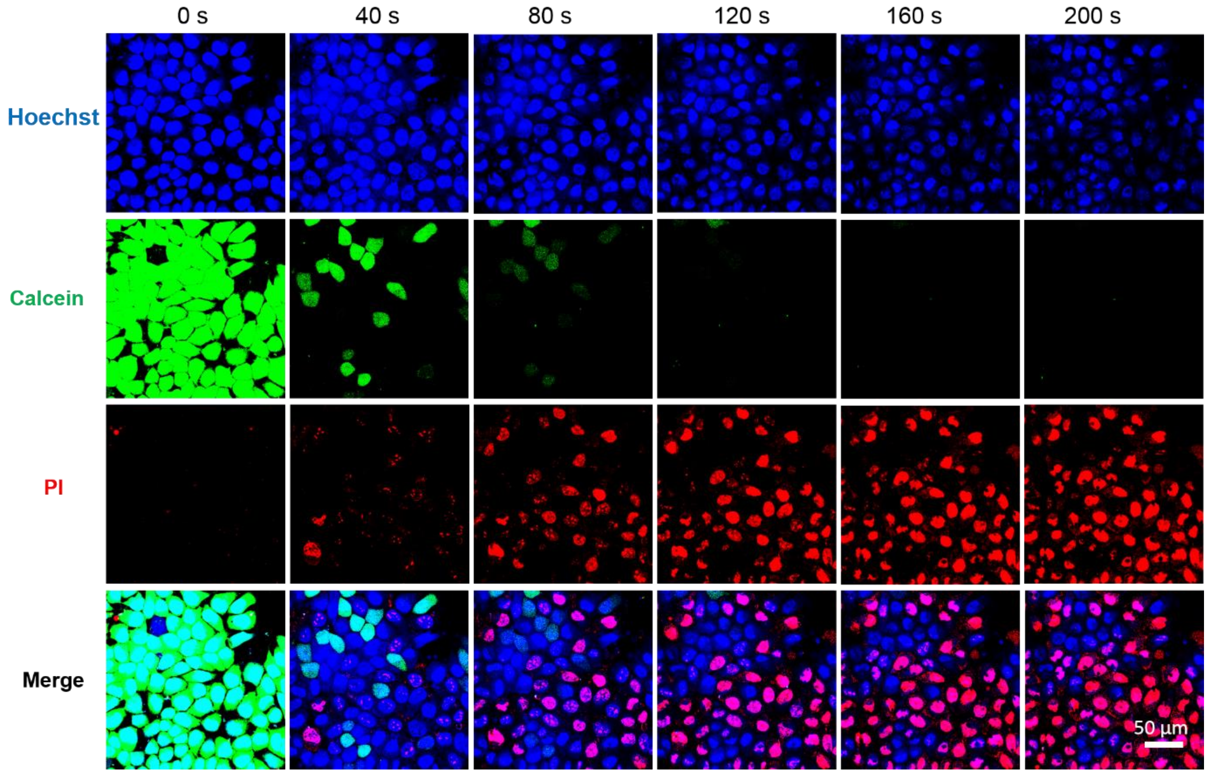

Supplementary Figure 65. Spatially controlled necrosis of HeLa cells with TP-NIR laser irradiation. HeLa cells were incubated with AIE/Au nanomotors and irradiated with a confocal TP-NIR laser (200 s irradiation in total, each TP-NIR scan is 4 s, Output power: 0.4 J cm^{-2}, TP-NIR wavelength was set at 760 nm). (a) Before confocal TP-NIR laser irradiation, calcein stain indicated that all cells are alive. (b) After confocal TP-NIR laser irradiation, the cells in the area of irradiation underwent a fast necrosis process and most of the irradiated cells were dead (indicated by PI staining). (c) Zoom out of the irradiated area highlights the viability of HeLa cells, in the absence of TP-NIR laser irradiation; only cells exposed to TP-NIR irradiation are dead. This further proves that TP-NIR laser irradiation allows controlled spatial treatment. Blue color indicates the nucleus staining dye Hoechst; Green color indicates calcein staining (live cells); red color represents PI (dead cells).

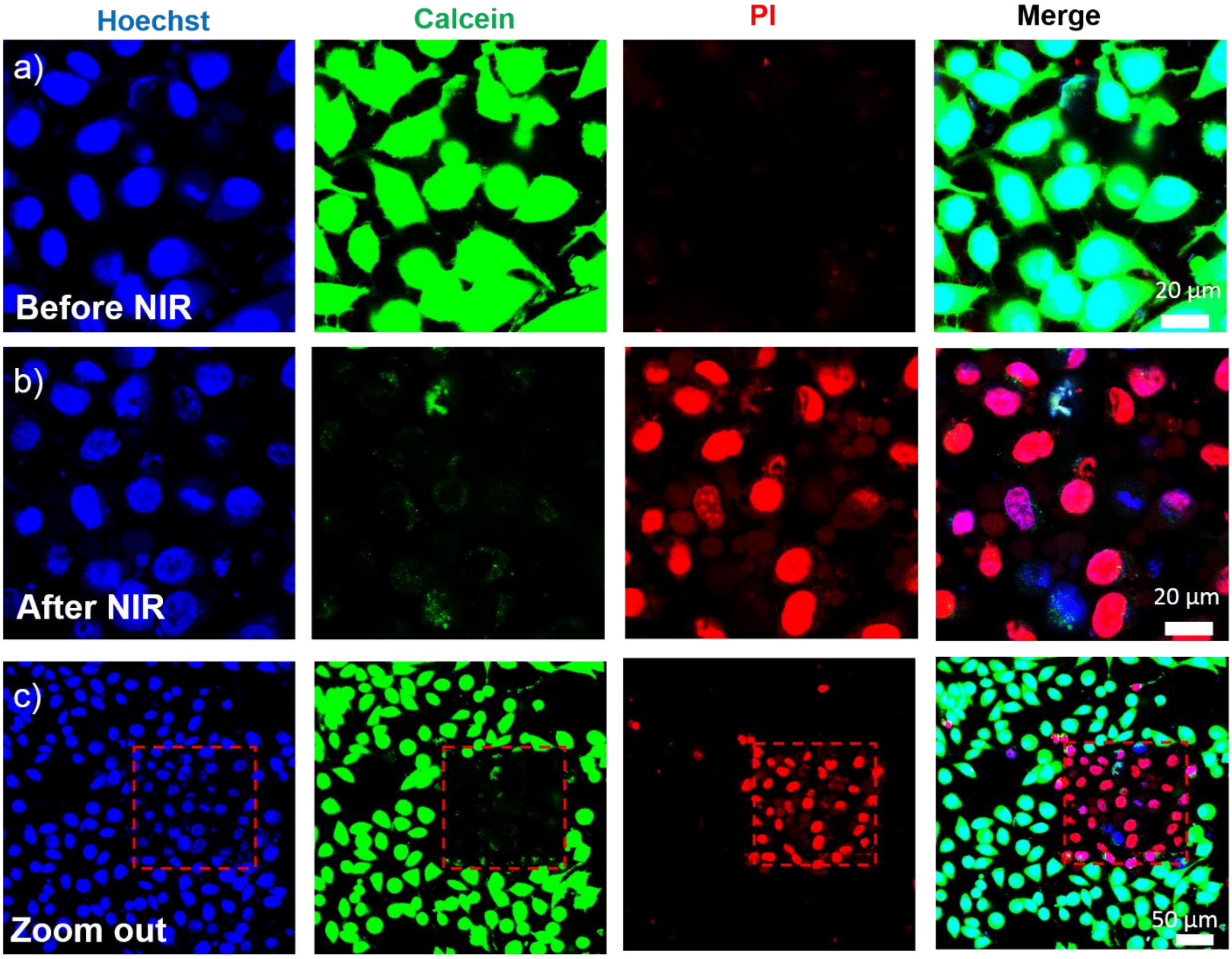

Supplementary Figure 66. Dose-dependence of the therapeutic effect of AIE/Au nanomotors on HeLa cells treated with PBS (blank) (a), 100 times diluted AIE/Au nanomotors (b, 0.25 μg ml^{-1}), 50 times diluted AIE/Au nanomotors (c, 0.5 μg ml^{-1}), 10 times diluted AIE/Au nanomotors (d, 2.5 μg ml^{-1}), and undiluted AIE/Au nanomotors (e, 25 μg ml^{-1}). After 200 s TP-NIR irradiation (each TP-NIR irradiation scan is 4 s, output power: 0.4 J cm^{-2}, TP-NIR wavelength was set at 760 nm), cells were imaged using confocal microscopy. Blue color indicates the nucleus staining dye Hoechst; Green color indicates calcein staining (live cells); red color represents AIE/Au nanomotor emission; Cyan stain represents PI staining (dead cells)

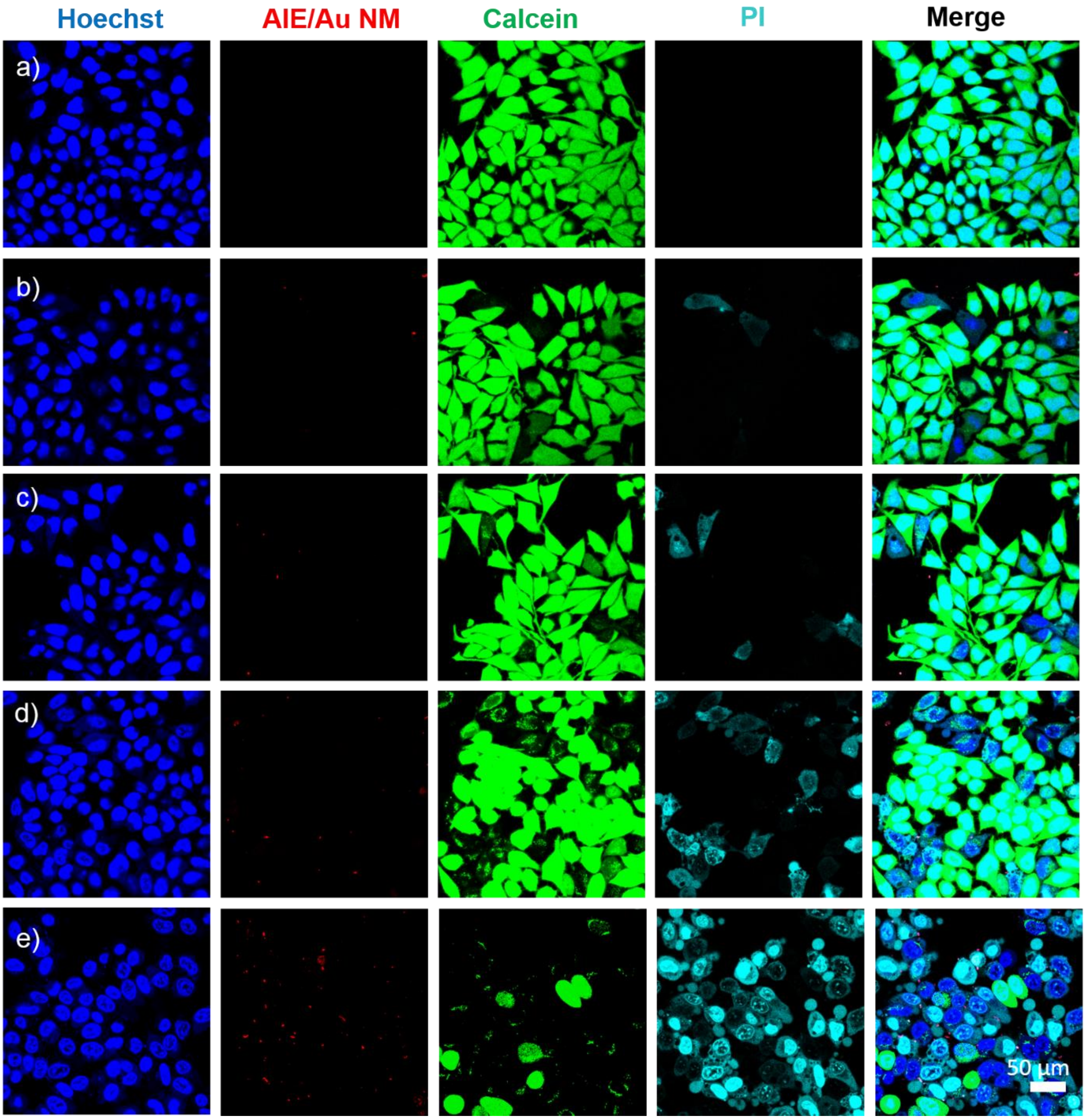

Supplementary Figure 67. Time-dependent therapeutic effect of HeLa cells treated with PBS (blank), 100 times diluted AIE/Au nanomotors (0.25 μg ml^{-1}), 50 times diluted AIE/Au nanomotors (0.5 μg ml^{-1}), 10 times diluted AIE/Au nanomotors (2.5 μg ml^{-1}), and undiluted AIE/Au nanomotors (25 μg ml^{-1}). Cells were irradiated and imaged using confocal TP-NIR (4 s per irradiation, output power: 0.4 J cm^{-2}, TP-NIR wavelength was set at 760 nm). Cyan represents PI (dead) stain.

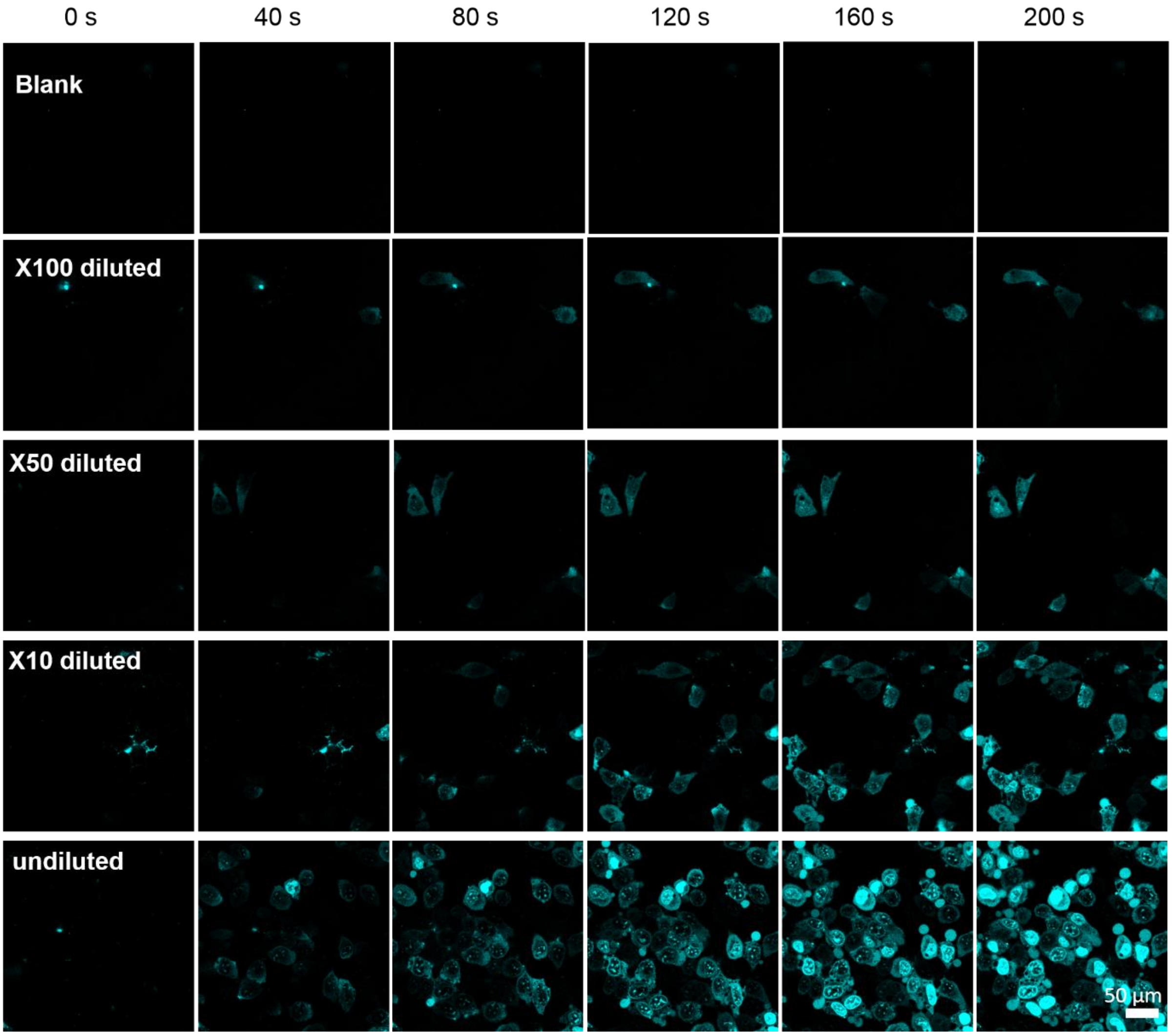

Supplementary Figure 68. ROS production of HeLa cells treated with PBS (blank) and non-AIE nanomotors (from PEG-PDLLA polymersomes, 25 μg ml^{-1}) after 80 s continuous confocal TP-NIR laser illumination (4 s per irradiation, output power: 0.4 J cm^{-2}, TP-NIR wavelength was set at 760 nm). From the confocal analysis, it can be concluded that even under prolonged TP-NIR irradiation (80 s, 4 s per TP-NIR irradiation), there is negligible increase of ROS signal, indicating negligible ROS production. Blue color indicates cell nucleus stain. Green color represents ROS product of DCFH-DA.

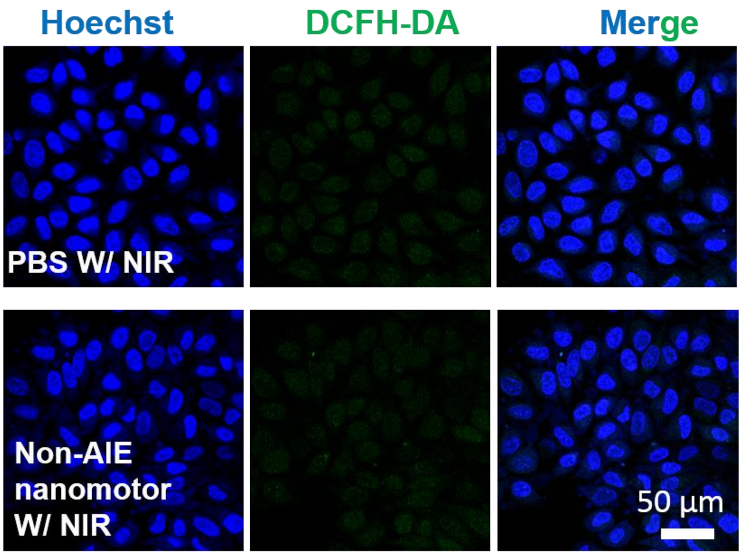

Supplementary Figure 69. ROS production of HeLa cells treated with wash-free non-AIE nanomotors (from PEG-PDLLA polymersomes, 25 μg ml^{-1}) under confocal TP-NIR laser illumination (4 s per TP-NIR irradaition, output power: 0.4 J cm^{-2}, TP-NIR wavelength was set at 760 nm). From the confocal analysis, it can be concluded that even under prolonged TP-NIR irradiation (80 s, 4 s per TP-NIR irradiation), there is negligible increase of ROS signal, indicating less efficient ROS production. Blue color indicates cell nucleus stain. Green color represents ROS product of DCFH-DA.

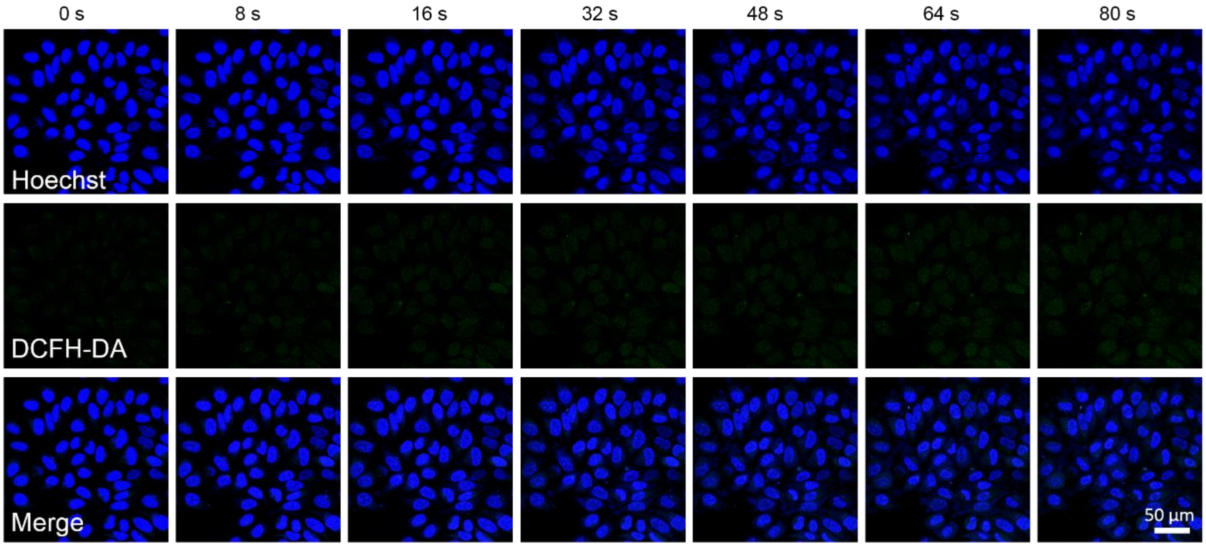

Supplementary Figure 70. Viability of HeLa cells treated with non-AIE nanomotors (from PEG-PDLLA polymersomes, 25 µg ml^{-1}) under confocal TP-NIR laser irradiation (200 s irradiation in total, 4 s per TP-NIR scan, output power: 0.4 J cm^{-2}, TP-NIR wavelength was set at 760 nm). PI stained a minor part of the cells at the end of TP-NIR irradiation. This indicates that non-AIE nanomotors, under TP-NIR laser irradiation, produce a photo-thermal effect which leads to necrosis. It is however clear that this process is less efficient when compared to AIE/Au nanomotors (that combine both photo-dynamic therapy and photo-thermal therapy in a single nanoparticle). Blue color indicates the nucleus staining dye Hoechst; Green color indicates calcein staining (live cells); red color represents PI (dead cells).

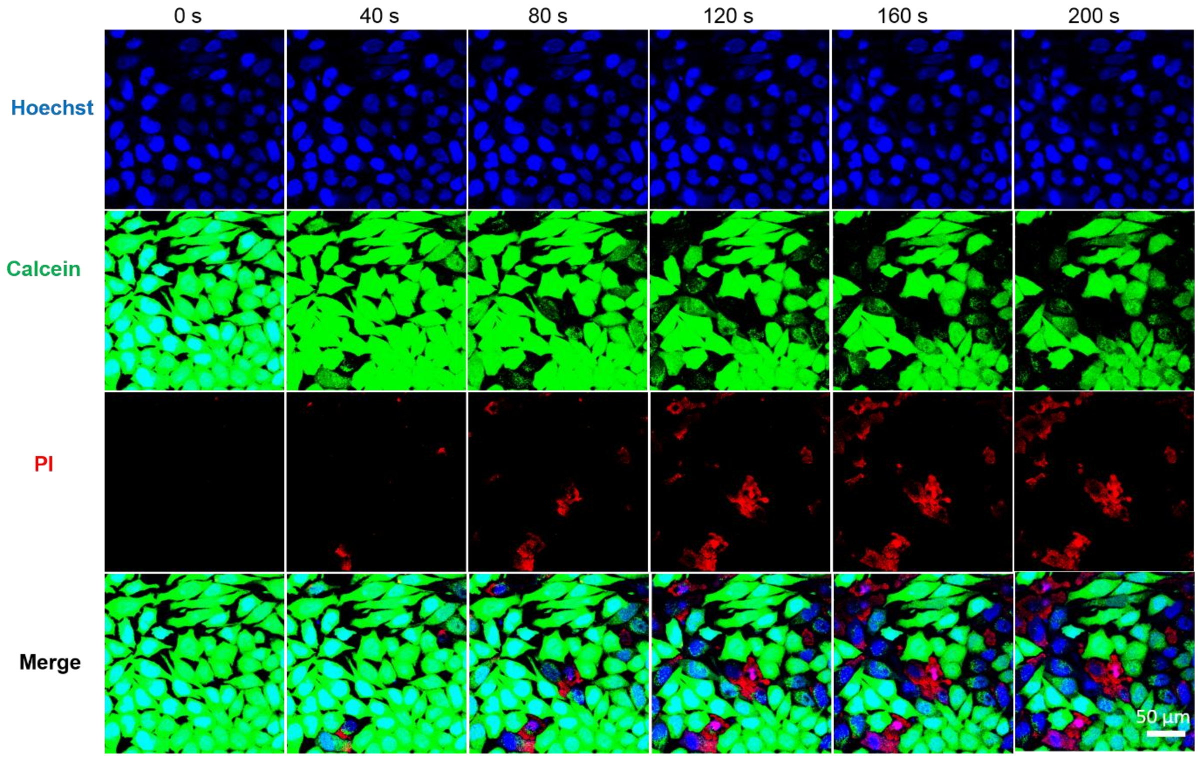

Supplementary Figure 71. Viability of 3D multicellular spheroids composed of HeLa cells after being treated with AIE/Au nanomotors (50 µg ml^{-1}). After 200 s confocal laser irradiation (in total 50 scans, per NIR irradiation is 4 s, output power: 0.4 J cm^{-2}, TP-NIR wavelength was set at 760 nm), cells were imaged using confocal microscopy. From the confocal analysis, PI gradually stained the nucleus of HeLa cells in the peripheral area of the spheroids, which indicated that the combined motion, photo-dynamic therapy and photo-thermal therapy led to necrosis in a 3D tumor mimicking model. Green color indicates calcein staining (live cells), red stain represents PI (dead cells).

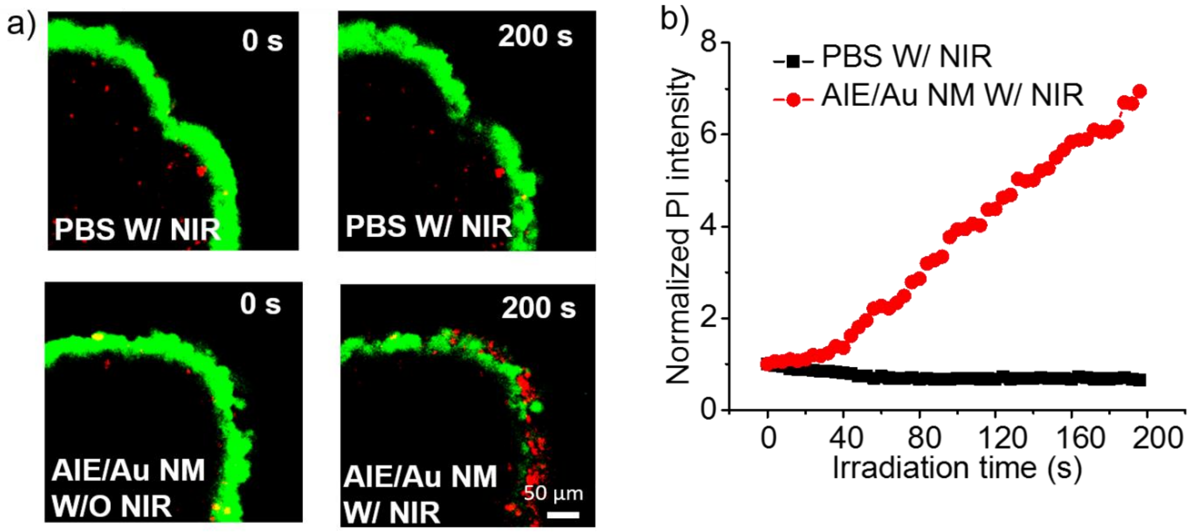

Reference:

1. Camelio, A. M., Wright, R. J., Knight, N. T. & Jazdzewski, B. A. Ir-catalyzed c-h amidation and borylation of anthraquinones. *J. Org. Chem.* **84**, 4940-4947 (2019).
2. Du, X. B. et al. Efficient non-doped near infrared organic light-emitting devices based on fluorophores with aggregation-induced emission enhancement. *Chem. Mater.* **24**, 2178-2185 (2012).
3. Gu, B. et al. Precise two-photon photodynamic therapy using an efficient photosensitizer with aggregation-induced emission characteristics. *Adv. Mater.* **29**, 1701076 (2017).
4. Ren, Y. et al. Pressure-induced neutral-to-ionic transition in an amorphous organic material. *Chem. Mater.* **28**, 6446-6449 (2016).
5. Sanders, D. P. et al. A simple and efficient synthesis of functionalized cyclic carbonate monomers using a versatile pentafluorophenyl ester intermediate. *J. Am. Chem. Soc.* **132**, 14724-14726 (2010).
6. Cao, S. P. et al. Ph-induced transformation of biodegradable multilamellar nanovectors for enhanced tumor penetration. *ACS Macro Lett.* **7**, 1394-1399 (2018).
7. Shao, J. X. et al. Erythrocyte membrane modified janus polymeric motors for thrombus therapy. *ACS Nano* **12**, 4877-4885 (2018).

8. Pijpers, I. A. B., Abdelmohsen, L. K. E. A., Williams, D. S. & van Hest, J. C. M. Morphology under control: Engineering biodegradable stomatocytes. *ACS Macro Lett.* **6**, 1217-1222 (2017).
9. Ma, X., Hahn, K. & Sanchez, S. Catalytic mesoporous janus nanomotors for active cargo delivery. *J. Am. Chem. Soc.* **137**, 4976-4979 (2015).
10. Che, H. et al. Atp-mediated transient behavior of stomatocyte nanosystems. *Angew. Chem. Int. Ed.* **58**, 13113-13118 (2019).
11. Wilson, D. A., Nolte, R. J. M. & van Hest, J. C. M. Autonomous movement of platinum-loaded stomatocytes. *Nat. Chem.* **4**, 268-274 (2012).
12. Abdelmohsen, L. K. E. A. et al. Dynamic loading and unloading of proteins in polymeric stomatocytes: Formation of an enzyme-loaded supramolecular nanomotor. *ACS Nano* **10**, 2652-2660 (2016).
