# Supplementary material for: Photoactivated nanomotors via aggregation induced emission for enhanced phototherapy
Source: Nat Commun. 2021 Apr 6;12:2077. doi: 10.1038/s41467-021-22279-w (PMC8024279; doi:10.1038/s41467-021-22279-w)
Supplement: Supplementary file 3 — Description of Additional Supplementary Files [file 41467_2021_22279_MOESM3_ESM.pdf]

Description of Additional Supplementary Files

Supplementary Movie 1. Cryo-TEM tomography tilt-series of AIE/Au nanomotors

Supplementary Movie 2. Computer-generated cross sections from the AIE/Au nanomotor in Z-axis

Supplementary Movie 3. Motion behavior of AIE/Au nanomotors without TP-NIR irradiation in PBS

Supplementary Movie 4. Motion behavior of AIE/Au nanomotors with TP-NIR irradiation (0.1 J cm^{-2}) in PBS

Supplementary Movie 5. Motion behavior of AIE/Au nanomotors with TP-NIR irradiation (0.4 J cm^{-2}) in PBS

Supplementary Movie 6. Nanoparticle tracking video of AIE/Au nanomotors without NIR laser irradiation in Milli-Q water

Supplementary Movie 7. Nanoparticle tracking video of AIE/Au nanomotors with NIR laser irradiation in Milli-Q water

Supplementary Movie 8. Nanoparticle tracking video of AIE/Au nanomotors without NIR laser irradiation in pure cell medium

Supplementary Movie 9. Nanoparticle tracking video of AIE/Au nanomotors with NIR laser irradiation in pure cell medium

Supplementary Movie 10. Nanoparticle tracking video of AIE-Ps with NIR laser irradiation in Milli-Q water

Supplementary Movie 11. Nanoparticle tracking video of AIE-Ps without NIR laser irradiation in Milli-Q water

Supplementary Movie 12. Nanoparticle tracking video of gold nanoshells without NIR laser irradiation in Milli-Q water

Supplementary Movie 13. Nanoparticle tracking video of gold nanoshells with NIR laser irradiation in Milli-Q water

Supplementary Movie 14. Membrane stained (green) HeLa cells upon interaction with AIE/Au nanomotors without TP-NIR irradiation

Supplementary Movie 15. Membrane disruption of HeLa cells (green = membrane stain) by AIE/Au (red) nanomotors activated by TP-NIR irradiation

Supplementary Movie 16. AIE/Au nanomotors activated by TP-NIR irradiation induces ROS production in HeLa cells (blue = nuclear stain, green = CM-H2DCFDA, ROS indicator)

Supplementary Movie 17. HeLa cells (blue = nuclear stain, green = calcein, live stain) treated with AIE/Au nanomotors without TP-NIR irradiation shows no toxicity (red = PI, dead stain)

Supplementary Movie 18. HeLa cells (blue = nuclear stain, green = calcein, live stain) treated with AIE/Au nanomotors with TP-NIR irradiation shows significant toxicity (red = PI dead stain)
